# Global Prevalence of Oral Potentially Malignant Disorders: An Updated Systematic Review and Meta‐Analysis

**DOI:** 10.1111/jop.70146

**Published:** 2026-04-28

**Authors:** Nicole Lonni, Tulio Silva Rosa, Camilla Kammer Pereira, Gilberto Melo, Saman Warnakulasuriya, Eliete Neves Silva Guerra, Elena Riet Correa Rivero

**Affiliations:** ^1^ Postgraduate Program in Dentistry, Health Sciences Center Universidade Federal de Santa Catarina Florianópolis Brazil; ^2^ Department of Public Health, Health Sciences Center Universidade Federal de Santa Catarina Florianópolis Brazil; ^3^ Faculty of Dentistry, Oral & Craniofacial Sciences, King's College London, England, and WHO Collaborating Centre for Oral Cancer London UK; ^4^ Laboratory of Oral Histopathology, School of Health Sciences Universidade de Brasília Brasília Brazil; ^5^ Oral Pathology Department, Health Sciences Center Universidade Federal de Santa Catarina Florianópolis Brazil

**Keywords:** oral potentially malignant disorders, prevalence, systematic review

## Abstract

**Background:**

Oral potentially malignant disorders (OPMDs) represent a diverse group of mucosal disorders with varying risks of progression to oral cancer.

**Objective:**

This updated systematic review (SR) and meta‐analysis (MA) estimated the global prevalence of histopathologically confirmed OPMDs, excluding oral lichen planus and lichenoid lesions.

**Methods:**

A comprehensive literature search was conducted using predefined eligibility criteria. A total of 108 studies were included, comprising 86 additional studies and 22 from a previous SR.

**Results:**

The overall pooled prevalence of OPMDs was 4.67% (95% CI: 3.62–5.84). Prevalence of OPMDs was as follows: Asia (5.44%), followed by South America/Caribbean (5.21%), and Europe (4.89%). Considering OPMD cases, the proportion was higher among males (62.87%) and individuals aged ≥ 50 years (68.1%). Histopathological evidence of epithelial dysplasia was present in 46.21% of cases. Oral leukoplakia (OL), oral submucous fibrosis (OSMF), and actinic cheilitis (AC) emerged as the most prevalent lesions, with relevant differences considering the geographic regions. OL and OSMF were the most common OPMDs in Asia, while OL and AC predominated in South America/Caribbean, and in Europe OL remains the most frequently encountered OPMD.

**Conclusions:**

The global prevalence of OPMDs, estimated at approximately 5%, should be interpreted as a broad summary measure, given the substantial heterogeneity driven by regional variations possibly related to behavioral, biological, and/or environmental risk factors. Future preventive efforts should focus on the type of OPMD prevalent in the region/country. The heterogeneity across studies highlights the need for standardized, population‐based studies using WHO nomenclature to better define the global burden.

## 1 Introduction

Lip and oral cavity cancers are projected to reach 642 000 new cases and 316 000 deaths between 2022 and 2050 [1]. Risk factors include genetic and epigenetic alterations, environmental exposure to ultraviolet (UV) radiation (for lip cancer), and lifestyle‐related factors such as tobacco, alcohol, and areca nut consumption (for oral cancer), all of which are closely associated with its occurrence and progression [2]. Cancer treatment is complex and associated with morbidity [3], potentially altering appearance and function, and disrupting daily life [4].

An effective strategy to prevent complications is early‐stage diagnosis. Many pathological alterations have been documented in normal mucosa during oral cancer progression, defined as oral potentially malignant disorders (OPMDs) [5, 6]. OPMDs represent a group of mucosal conditions associated with an increased risk of progression to oral cancer, including oral leukoplakia (OL), proliferative verrucous leukoplakia (PVL), oral erythroplakia (OE), oral submucous fibrosis (OSMF), oral lichen planus (OLP), oral lichenoid lesions (OLL), actinic cheilitis (AC), and others [7].

Histopathological examination remains the gold standard for OPMD diagnosis, with emphasis on the identification and grading of epithelial dysplasia [7, 8]. The presence of dysplasia reflects an environment of chromosomal instability, where the risk of malignant transformation progressively increases the susceptibility to develop cancer, either at the site of the existing OPMD or anywhere in the mouth during lifetime [9, 10, 11]. Reported malignant transformation rates of OPMDs vary across studies and are influenced by both the specific clinical subtype [12, 13, 14, 15] of the disorder and the severity of epithelial dysplasia [16].

In 2018, the global prevalence of OPMDs was estimated by Mello et al. at 4.47% (95% Confidence Interval [95% CI]: 2.43–7.08) in what has remained the only comprehensive synthesis to date [17]. Therefore, the aim of this SR is to provide an updated answer to the following question: “What is the pooled global prevalence of oral potentially malignant disorders?”

## 2 Materials and Methods

### 2.1 Protocol and Registration

A SR protocol was designed following the Preferred Reporting Items for Systematic Reviews and Meta‐Analyses Protocols (PRISMA‐P) [18] and registered in the International Prospective Register of Systematic Reviews (PROSPERO: CRD42024533099) [19]. This SR adhered to the PRISMA checklist [20].

### 2.2 Eligibility Criteria

Observational studies investigating OPMD prevalence were included. Diagnosis must have been confirmed by histopathological examination based on the WHO criteria [8]. Only studies from 2017 onward were considered, as this is an update of a previous SR [17]. This update also included studies with patients under 18 years of age. To improve external validity of results, studies previously excluded solely due to this age criterion were re‐evaluated and included. Consistent with the prior review, prevalence data for OLP are not reported here.

The following exclusion criteria were applied to studies where: (1) OPMD data could not be extracted due to grouping with other conditions; (2) OPMDs were only linked to specific etiological factors (e.g., HPV, betel quid, tobacco); (3) diagnosis of OPMD was not confirmed by histopathological analysis; (4) prevalence of OPMD was not clearly reported or could not be calculated; (5) reviews, case reports, protocols, short communications, personal opinions, letters, posters, conference abstracts, thesis, dissertations, and laboratory research; (6) full texts were not available; (7) only oral lichen planus or other lichenoid lesions were included in the analysis, and (8) published in languages other than the Latin (Roman) alphabet.

### 2.3 Information Sources and Search Strategy

Search strategies were developed for five databases: MEDLINE/PubMed, Embase, Scopus, Web of Science, and Latin American and Caribbean Health Sciences (LILACS/BBO), as well as EBSCO. Additionally, a Grey literature search was conducted using Google Scholar and ProQuest Dissertations & Thesis Global. Searches were updated on March 14th, 2025 (Appendix S1). The reference lists of the included articles were also manually screened for eligible studies. Duplicate records were removed using the reference management software EndNote 20 (Clarivate Analytics, Philadelphia, PA, USA).

### 2.4 Study Selection, Data Collection, and Data Items

The selection process was conducted by three independent reviewers (N.L., T.S.R., C.K.P.) in two phases, in a blinded manner, with cross‐checking of their assessments at the end of each phase. In phase‐1, titles and abstracts of all records were screened and, in phase‐2, the full‐text articles were assessed. Subsequently, the following key data were extracted from included studies: first author, publication year, country, study design, population characteristics (sample size, sex distribution, age, and risk factors), histopathological diagnoses, OPMD type and prevalence. Discrepancies were resolved with a fourth reviewer (G.M.).

### 2.5 Methodological Quality in Individual Studies

The methodological quality (MQ) was evaluated using the Joanna Briggs Institute (JBI) Critical Appraisal Checklist for studies reporting prevalence data [21]. Three reviewers independently (N.L., T.S.R., C.K.P.) assessed MQ and cross‐checked the findings. Ratings were expressed as the percentage of “yes” responses per question, with higher values indicating better quality.

### 2.6 Effect Measures

The primary outcome was the prevalence of OPMD, reported as relative frequency with 95% CI. Secondary outcomes included the prevalence based on geographic location, demographic characteristics, clinical diagnosis, and the presence and severity of epithelial dysplasia.

### 2.7 Synthesis of Methods

A proportion meta‐analysis (MA) was performed using R version 4.4.3 (The R Foundation, Vienna, Austria). The meta package was employed, and the Freeman‐Tukey double arcsine transformation was used for the overall proportion estimates and a random‐effects model was adopted [22]. Statistical heterogeneity was assessed by Cochran's *Q* (*χ* ^2^), *I* ^2^, and the prediction interval. Subgroup analyses were performed according to clinical and histological diagnosis, geographic region, age, and gender. Sensitivity meta‐analyses were conducted by excluding studies that only considered lesions occurring in a predetermined anatomical location, such as lip, palate, tongue, or gingiva.

## 3 Results

### 3.1 Studies Selection

**FIGURE 1:**
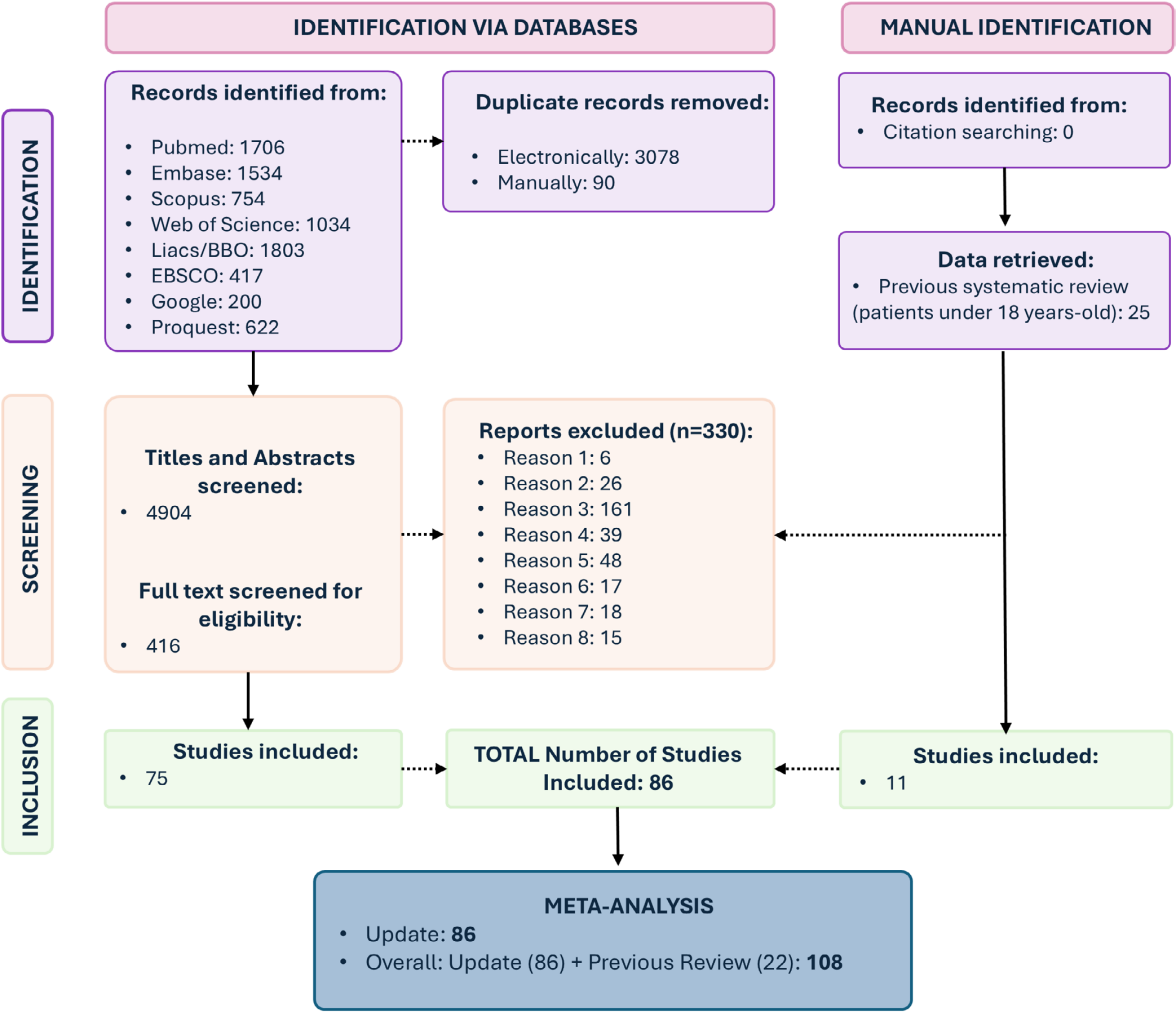
PRISMA flow diagram outlining the study selection process. Exclusion criteria (reasons) applied: (1) OPMD data could not be extracted due to grouping with other conditions; (2) OPMDs were only linked to specific etiological factors (e.g., HPV, betel quid, tobacco); (3) diagnosis of OPMD was not confirmed by histopathological analysis; (4) prevalence of OPMD was not clearly reported or could not be calculated; (5) reviews, case reports, protocols, short communications, personal opinions, letters, posters, conference abstracts, thesis, dissertations, and laboratory research; (6) full texts were not available; (7) only oral lichen planus or other lichenoid lesions were included in the analysis, and (8) published in languages other than the Latin (Roman) alphabet.

A total of 8070 records were identified. The selection process is shown in the PRISMA flow diagram (Figure 1), with exclusion reasons during phase‐2 detailed in Appendix S2. Ultimately, 75 new studies met the eligibility criteria. Additionally, 11 articles excluded from the previous SR [17] were reassessed due to removal of age restriction criteria, resulting in 86 additional studies. The 22 articles already assessed in the previous SR [17] were integrated in the synthesis of results section, totaling 108 studies reported here.

### 3.2 Study Characteristics (*n* = 86)

Study characteristics of the 22 studies included in the previous SR are available elsewhere [17]. Of the additional studies (Appendix S3), 174 265 new OPMD cases were included. Age distribution was heterogeneous, ranging from early childhood to over 90 years. Notably, 68 (79%) studies did not report age‐specific data. Concerning geographical location, studies were conducted in South American/Caribbean countries (*n* = 31), Asia (*n* = 34), Europe (*n* = 12), Middle East (*n* = 6), and North America (*n* = 3). The study samples were sourced from pathology laboratory services (*n* = 34), hospitals (*n* = 14), universities or medical schools (*n* = 19), or from the general population (*n* = 19). Moreover, most studies reported separate data considering clinical diagnoses, however, different OPMD were grouped together in 8 studies.

### 3.3 Methodological Quality Within Studies (*n* = 86)

MQ of the 22 studies included in the previous SR are available elsewhere [17]. Of additional studies (Appendix S4), MQ ratings were lower considering representativeness of the target population and the adequacy of the sample size. These concerns stemmed from the use of non‐random sampling methods and the absence of population‐based screening strategies. Since histopathological confirmation was an inclusion criterion, all studies presented higher MQ ratings for condition identification methods. However, standardization and reliability of methods presented lower MQ ratings in studies that presented an inadequate clinical definition of OPMDs. Data analysis coverage and response rate management were considered not applicable in most cases since most studies relied on convenience samples. As such, participant attrition and response rates were not considered relevant factors. Finally, with respect to statistical analysis, only studies that reported prevalence estimates with 95% CI were given higher MQ.

### 3.4 Results of Individual Studies (*n* = 86)

Prevalence of OPMD per study basis of the 22 studies included in the previous SR are available elsewhere [17]. Considering the additional studies, prevalence of OPMD in individual studies showed marked variability, ranging from 0.03% to 42.86%. When stratified by geographical region, prevalence ranges per study basis were: Asia (0.06%–42.86%), South America/Caribbean (0.41%–37.50%), North America (0.03%–36.71%), Europe (0.35%–13.43%), and Middle East (0.23%–7.81%). Data on exposure to risk factors were reported or could be extracted in only 20 studies. Tobacco use, both smoked and smokeless, was the predominant risk factor, accounting for 75.2% of all reported exposures. This was followed by combined exposures (17.3%), most frequently involving the concurrent use of tobacco and alcohol.

### 3.5 Synthesis of Results

**FIGURE 2:**
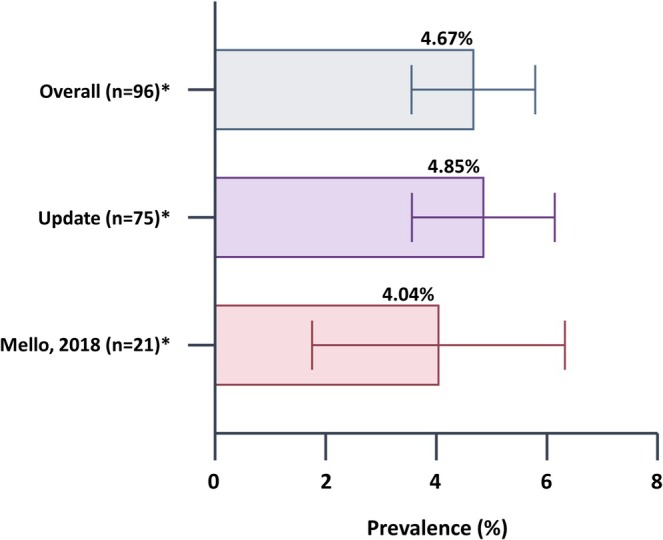
Prevalence of the OPMDs. The pooled prevalence of OPMDs was estimated 4.67% (*n* = 96). Considering only the additional updated studies (*n* = 75), prevalence was 4.85%, which was considered comparable to previous findings (4.04%). *The reported number of studies (*n*) reflects inclusion based on sensitivity analysis.

This section describes the pooled results of studies included in MA. Considering only the 22 studies from the previous SR [17], pooled prevalence was estimated at 4.42% (95% CI: 2.33–7.13). Considering all 108 studies, the overall prevalence of OPMDs was 5.17% (95% CI: 4.07–6.40; Appendix S5). Sensitivity analysis affected overall prevalence; the exclusion of 12 studies lowered the overall prevalence to 4.67 (95% CI: 3.62–5.84; Appendix S6; Figure 2). Therefore, all subsequent prevalence analyses were performed excluding studies focusing on predefined anatomical locations.

**FIGURE 3:**
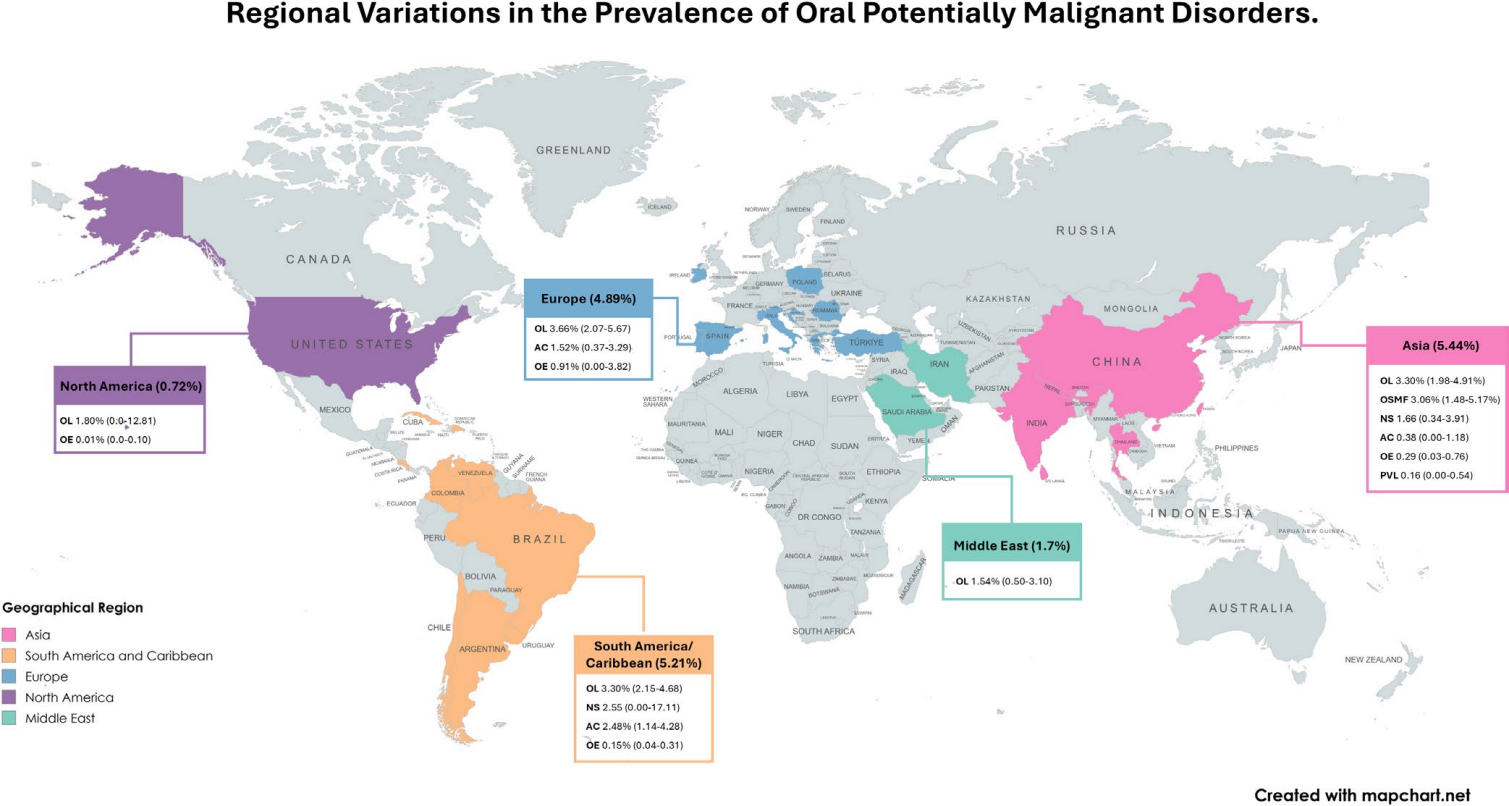
Pooled subgroup prevalence of OPMD according to clinicopathological diagnosis and geographic region, with corresponding 95% confidence intervals (%). The map displays only lesion subgroups supported by data from more than two studies in the meta‐analysis. AC, actinic cheilitis; OE, oral erythroplakia; OL, oral leukoplakia; OSMF, oral submucous fibrosis; PVL, proliferative verrucous leukoplakia; NS, non‐specified oral potentially malignant disorder.

Pooled prevalence by clinical diagnosis (Figure 3 and Appendix [Link], [Link]) was analyzed according to geographical region, given that variation in exposure to specific risk factors may influence the distribution of clinical subtypes. Of the 108 studies, 84 reported data on OL, of which 13 further classified it into homogeneous (HOL) and/or non‐homogeneous (NHOL) subtypes, with the latter including leukoerythroplakia and erythroleukoplakia. The most prevalent lesion across all regions was OL: Asia (3.30%; 95% CI: 1.98%–4.91%), South America and the Caribbean (3.30%; 95% CI: 2.15%–4.68%), Europe (3.66%; 95% CI: 2.07%–5.67%), and Middle East (OL 1.54%; 95% CI: 0.50%–3.10%). The second most prevalent lesion in Asia was OSMF (3.06%; 95% CI: 1.48%–5.17%), whereas in South America and Europe, the second most prevalent lesion was AC (2.48%; 95% CI: 1.14%–4.28% and 1.52%; 95% CI: 0.37%–3.29%, respectively).

The pooled prevalence considering sampling sources (Appendix S13) was higher in hospitals records (7.31%; 95% CI: 3.09%–13.06%). In contrast, lower prevalence estimates were observed in pathology services (5.09%; 95% CI: 3.62%–6.79%), population‐based studies (3.74%; 95% CI: 2.03%–5.92%), and samples from universities (3.52%; 95% CI: 1.74%–5.86%). The pooled prevalence per geographical location (Appendix S14) were as follow: Asia (5.44%, 95% CI: 3.37%–7.96%), South America/Caribbean (5.21%, 95% CI: 3.56%–7.13%), Europe (4.89%, 95% CI: 2.85%–7.42%), Middle East (1.7%, 95% CI: 0.73%–3.02%), and North America (0.72%, 95% CI: 0.00%–3.76%). An overview of pooled prevalence estimates stratified by country is available in Appendix S15.

Additionally, analyses were conducted to examine the distribution of characteristics among OPMD cases. It is important to note that the following estimates are based on subgroup data rather than the overall sample. Therefore, they should be interpreted as proportions within the subgroup, not as prevalence in the general population. The number of studies and OPMD lesions for each analysis are described in the appendices. For age groups (Appendix S16), individuals over 50 years (68.10%; 95% CI: 54.90%–80.01%) were proportionally more affected compared to under 50 years (36.94%; 95% CI: 24.53%–50.28%). Regarding sex (Appendix S17), males (62.87%; 95% CI: 57.30–68.28) were proportionally more affected than females (37.03%; 95% CI: 31.77%–42.43%). Considering histopathological diagnosis (Appendix S18), dysplastic lesions (46.21%; 95% CI: 31.73%–61.00%) were proportionally more observed than non‐dysplastic lesions (36.75%; 95% CI: 22.68%–51.97%).

### 3.6 Reporting Bias Assessment

Detailed information was lacking considering histopathological data, patient demographics, etiological factors, and clinical OPMD subtypes. Considering studies from both the previous SR [17] and the present update, data on risk factors could be extracted from only 28 studies. Notably, only 48 studies provided explicit information on the presence or absence of epithelial dysplasia. Moreover, the grouped reporting of multiple OPMD resulted in the exclusion of study data in several analyses.

## 4 Discussion

Various terms have been employed to describe what are collectively referred as OPMD, including “precancer”, “epithelial precursor lesions”, “premalignant”, “precancerous” [7]. These terminologies were discouraged in the latest WHO collaborating centre's consensus paper; however, inconsistency in classification remains a major challenge [23]. Considering that oral lichen planus and lichenoid lesions can be classified as either OPMD or immune‐mediated diseases, and were not included in the previous SR; for these reasons, these lesions were excluded from the present update to maintain methodological consistency.

The pooled prevalence of OPMD was 4.67%, which is comparable to previously reported estimates (4,47%) [17]. However, heterogeneity was considered high, with a prediction interval of 0.00%–21.11%. A wide prediction interval indicates that single prevalence estimates from any given future study might fall within a broad range of values. This can be a result of methodological variability of included studies, exposure to different risk factors, and even cultural differences across global populations. Furthermore, the wide range of OPMD prevalence reported across individual studies (0.03% to 42.86%) highlights the influence of methodological heterogeneity, sampling source, and possible clinical misclassification. In this context, studies relying on pathology department records may be biased toward lesions that are more likely to be biopsied, thereby distorting prevalence estimates.

The highest prevalence rates of OPMDs were reported in Asia (5,4%), likely reflecting cultural, behavioral, and socioeconomic influences [24, 25]. In Asia, OL and OSMF were the most frequently reported OPMDs, consistent with regional lifestyle patterns and exposure to specific risk factors. The previous SR estimated a 10% prevalence of OPMDs in Asia based on only five studies conducted in Taiwan, Sri Lanka, India, and Thailand [17]. In contrast, the current analysis includes 38 studies from Asia, which adds data from China, Taiwan, Sri Lanka, India, and Thailand. Most studies originated from India, where cultural habits and exposures differ from other Asian countries, contributing to observed heterogeneity [26]. In South and Southeast Asia, the habitual use of areca nut and betel quid [27], often in combination with tobacco, remains deeply embedded in cultural and social practices, despite their known carcinogenicity [26]. This may explain the high occurrence of OL and OSMF in these regions. Also, OL is a clinical diagnosis of exclusion, and its identification can be challenging due to the presence of other oral white lesions with similar clinical appearances [7]. Consequently, these factors may lead to an overestimation of OL prevalences.

In South America, OL and AC showed the highest pooled prevalences. Notably, most studies reporting higher AC prevalence were conducted in tropical regions, particularly in Brazil. In this country, year‐round high ultraviolet radiation represents a major environmental risk factor for lip lesions [28]. This pattern likely explains the geographic clustering of AC cases across countries with intense sun exposure, affecting white skinned populations. In addition to UV‐related factors, the widespread use of tobacco and alcohol, often compounded by limited access to preventive health programs and adequate healthcare infrastructure, further contributes to the burden of OPMDs across this region [5]. Also, AC prevalence appears to be overestimated in studies focusing exclusively on lip lesions [29, 30, 31, 32, 33]. To provide more accurate AC estimates, these studies were excluded in sensitivity analyses performed in this SR.

The prevalence of OPMDs in parts of Europe aligns with recent increases in oral cancer incidence, suggesting a concerning trend in high‐income countries [25, 34]. OL remains the most frequent OPMD across Europe. While tobacco use has declined among younger populations, evidence indicates that it has increased among adults over the age of 45 [35]. This pattern may reflect the re‐emergence of risk behaviors, such as smoking and alcohol use, that were previously well‐controlled through public health measures [35, 36]. Additionally, disposable vapes have gained popularity due to their high nicotine concentrations, low cost, discreet design, and ease of use [35]. Also, alcohol consumption levels in Europe rank among the highest per capita worldwide [37]. Furthermore, in many southern European countries, susceptibility increases to UV‐induced lip damage, which may partly explain cases of AC observed in this region. These contextual and behavioral factors collectively sustain high levels of exposure and explain the elevated burden of OPMDs, while also signaling the need for renewed prevention strategies in high‐income settings.

The age and sex distribution observed in this SR aligns with the well‐established demographic profile of OPMD patients: predominantly male and over 50 years of age [6, 38]. This SR found greater exposure among men to lifestyle risk factors, with tobacco as the most frequent habit, with or without concurrent alcohol consumption. Prolonged and combined exposures exert a synergistic carcinogenic effect [38], driving “field cancerization” through genomic instability and the accumulation of genetic alterations [39]. This trajectory parallels the typical age at oral cancer diagnosis, underscoring OPMDs as early precursors in oral carcinogenesis.

This SR remains the only updated synthesis of OPMD prevalence based solely on histopathologically confirmed diagnoses, thereby ensuring greater diagnostic accuracy. In this SR, the presence of epithelial dysplasia was consistently higher than non‐dysplastic lesions. Given that studies have reported a malignant transformation rate of approximately 10% among patients with any oral epithelial dysplasia [40, 41], these findings emphasize the role of accurate histopathological characterization in identifying high‐risk lesions and guiding clinical management. Although histopathological confirmation was performed in all studies, only 35 of the 108 studies reported detailed characterization or grading, representing a limitation that may lead to an overestimation of the prevalence of dysplastic lesions. This pattern could also reflect publication bias, as studies with dysplastic or higher‐risk lesions may be more likely to be published than those reporting non‐dysplastic lesions. Typically, a predominance of non‐dysplastic lesions would be expected, especially considering the higher prevalence of OL, which is frequently non‐dysplastic [12].

A notable issue identified in this SR was the frequent absence of clinical information in cases of epithelial dysplasia, as well as its misreporting as a clinical rather than a histopathological diagnosis. This mislabelling reflects a broader challenge in clinical practice, where the critical distinction between clinical presentation and histopathological features is often neglected [42]. The absence of detailed clinical information may hinder diagnostic precision and clinical management, especially considering that OE and PVL are associated with a higher risk of malignant transformation compared to other OPMD [43]. This reinforces the need for strict adherence to standardized reporting protocols [7, 8].

It is well established that the most reliable prevalence estimates are derived from population‐based studies; however, such studies remain scarce for OPMDs, particularly those including histopathological confirmation. Moreover, the lack of data from regions such as Africa and Oceania underscores important geographical gaps, limiting the global representativeness of current estimates. Consequently, the data presented in this SR should be interpreted with caution, as they may not fully reflect the true burden of OPMDs in the general population.

## 5 Conclusion

The global prevalence of OPMD, estimated at approximately 5%, should be interpreted as a broad summary estimate. Although OL was the most prevalent lesion, there was substantial variation by geographic region, especially for AC and OSMF, possibly related to behavioral, biological, and/or environmental risk factors. Accurately estimating global OPMD prevalence was hindered by inconsistent diagnostic criteria, variable screening practices, and limited access to specialized oral pathology services, particularly in low/middle‐income countries. Nonetheless, global efforts must incorporate measures to define populations with the highest attributable burden to guide preventive strategies worldwide.

## Author Contributions

**Elena Riet Correa Rivero, Eliete Neves Silva Guerra,** and **Saman Warnakulasuriya:** conceptualization. **Nicole Lonni, Tulio Silva Rosa,** and **Camilla Kammer Pereira:** methodology. **Nicole Lonni, Tulio Silva Rosa,** and **Gilberto de Souza Melo:** formal analysis and investigation. **Nicole Lonni:** writing – original draft preparation. **Elena Riet Correa Rivero:** funding acquisition and resources. **Elena Riet Correa Rivero** and **Eliete Neves Silva Guerra:** supervision. All authors contributed to the study conception and design, writing – review and editing.

## Funding

This study was funded by Conselho Nacional de Desenvolvimento Científico e Tecnológico (CNPq—403444/2023‐3; Research fellows E.R.C.R.: 305891/2024‐3 and E.N.S.G.: 310587/2023‐9); Coordenação de Aperfeiçoamento de Pessoal de Nível Superior—Brasil (CAPES—Finance Code 001: N.L. and T.S.R.). Fundação de Amparo à Pesquisa e Inovação do Estado de Santa Catarina (FAPESC, 984/2025: C.K.P.).

## Conflicts of Interest

The authors declare no conflicts of interest.

## Supporting information


**Appendix S1:** Database search strategy.

**Appendix S2:** Reasons for exclusion of studies following full‐text screening.

**Appendix S3:** Extracted data from included studies.

**Appendix S4:** Methodological quality of individual studies.

**Appendix S5:** Meta‐analysis of the global prevalence of oral potentially malignant disorders.

**Appendix S6:** Sensitivity meta‐analysis of the global prevalence of oral potentially malignant disorders.

**Appendix S7:** Sensitivity meta‐analysis of pooled prevalence by diagnosis in Asia.

**Appendix S8:** Sensitivity meta‐analysis of pooled prevalence by diagnosis in South America and the Caribbean.

**Appendix S9:** Sensitivity meta‐analysis of pooled prevalence by diagnosis in Europe.

**Appendix S10:** Sensitivity meta‐analysis of pooled prevalence by diagnosis in North America.

**Appendix S11:** Sensitivity meta‐analysis of pooled prevalence by diagnosis in the Middle East.

**Appendix S12:** Summary of clinical diagnosis by region.

**Appendix S13:** Sensitivity meta‐analysis of pooled prevalence by sample source.

**Appendix S14:** Sensitivity meta‐analysis of prevalence stratified by geographical region.

**Appendix S15:** Sensitivity meta‐analysis of pooled prevalence by country.

**Appendix S16:** Meta‐analysis of pooled proportion by patient age.

**Appendix S17:** Meta‐analysis of pooled proportion by patient sex.

**Appendix S18:** Meta‐analysis of pooled proportion by epithelial dysplasia.

**Data S1:** Supporting Information.

**Data S2:** PRISMA 2020 checklist.

## Data Availability

The data that supports the findings of this study are available in the Supporting Information of this article.
