# Supplementary material for: Global Prevalence of Oral Potentially Malignant Disorders: An Updated Systematic Review and Meta‐Analysis
Source: J Oral Pathol Med. 2026 Apr 28;55(7):747–54. doi: 10.1111/jop.70146 (PMC13429371; doi:10.1111/jop.70146)
Supplement: Supplementary file 1 — Appendix S1: Database search strategy. [file JOP-55-747-s019.docx]

**Appendix S1** - Database search strategy

| Database | Last update: March 14^th^, 2025 | Results | |
| --- | --- | --- | --- |
| Pubmed | ("Incidence"[Mesh] OR "Epidemiology"[Subheading] OR "Epidemiology"[Mesh] OR "Prevalence"[Mesh] OR "prevalence" OR "prevalences" OR "frequency" OR "frequencies" OR "epidemiology" OR "epidemiologic" OR "epidemiological" OR "occurrence" OR "occurrences" OR "incidence" OR "incidences") AND ("Erythroplasia"[Mesh] OR "Carcinoma in Situ"[Mesh] OR "Oral Submucous Fibrosis"[Mesh] OR "Cheilitis"[Mesh] OR "Leukoplakia"[Mesh] OR "precancerous" OR "pre-cancer" OR "precancer" OR "precursor lesion" OR "precursor lesions" OR premalignan* OR pre malignan* OR "leukoplakia" OR "leukoplakias" OR "leukoplakic" OR "keratosis" OR "keratoses" OR "leukokeratosis" OR "leukokeratosis" OR "erythroplasia" OR "erythroplasias" OR "erythroplakia" OR "erythroplakias" OR "Carcinoma in Situ" OR "in situ carcinoma" OR "carcinomas in situ" OR "in situ carcinomas" OR "preinvasive carcinoma" OR "preinvasive carcinomas" OR "pre-invasive carcinoma" OR "pre-invasive carcinomas" OR "intraepithelial carcinoma" OR "intraepithelial carcinomas" OR "intraepithelial neoplasm" OR "intraepithelial neoplasms" OR "intraepithelial neoplasia" OR "intraepithelial neoplasias" OR "intraepithelial câncer" OR "oral submucous fibrosis" OR "cheilitis" OR "actinic cheilosis" OR "potentially malignant") AND ("Mouth"[Mesh] OR "Lip"[Mesh] OR "Tongue"[Mesh] OR "Mouth Mucosa"[Mesh] OR "Palate"[Mesh] OR "Mouth Floor"[Mesh] OR "Gingiva"[Mesh] OR "mouth" OR "mouths" OR "oral" OR "orals" OR "lip" OR "lips" OR "tongue" OR "buccal" OR "palate" OR "palates" OR "cheek mucosa" OR "alveolar" OR "gingiva" OR "gengivas" OR "gum" OR "gums" OR "interdental papilla" OR "interdental papillae" OR "commissure" OR "maxillary tuberosity" OR "uvula" OR "uvular") + filtro 2017 | | **1706** |
| EMBASE | ('prevalence':ti,ab,kw OR 'prevalences':ti,ab,kw OR 'frequency':ti,ab,kw OR 'frequencies':ti,ab,kw OR 'epidemiology':ti,ab,kw OR 'epidemiologic':ti,ab,kw OR 'epidemiological':ti,ab,kw OR 'occurrence':ti,ab,kw OR 'occurrences':ti,ab,kw OR 'incidence':ti,ab,kw OR 'incidences':ti,ab,kw) AND ('precancerous':ti,ab,kw OR 'pre-cancer':ti,ab,kw OR 'precancer':ti,ab,kw OR 'precursor lesion':ti,ab,kw OR 'precursor lesions':ti,ab,kw OR premalignan*:ti,ab,kw OR 'pre malignan*':ti,ab,kw OR 'leukoplakia':ti,ab,kw OR 'leukoplakias':ti,ab,kw OR 'leukoplakic':ti,ab,kw OR 'keratosis':ti,ab,kw OR 'keratoses':ti,ab,kw OR 'leukokeratosis':ti,ab,kw OR 'erythroplasia':ti,ab,kw OR 'erythroplasias':ti,ab,kw OR 'erythroplakia':ti,ab,kw OR 'erythroplakias':ti,ab,kw OR 'carcinoma in situ':ti,ab,kw OR 'in situ carcinoma':ti,ab,kw OR 'carcinomas in situ':ti,ab,kw OR 'in situ carcinomas':ti,ab,kw OR 'preinvasive carcinoma':ti,ab,kw OR 'preinvasive carcinomas':ti,ab,kw OR 'pre-invasive carcinoma':ti,ab,kw OR 'pre-invasive carcinomas':ti,ab,kw OR 'intraepithelial carcinoma':ti,ab,kw OR 'intraepithelial carcinomas':ti,ab,kw OR 'intraepithelial neoplasm':ti,ab,kw OR 'intraepithelial neoplasms':ti,ab,kw OR 'intraepithelial neoplasia':ti,ab,kw OR 'intraepithelial neoplasias':ti,ab,kw OR 'intraepithelial câncer':ti,ab,kw OR 'oral submucous fibrosis':ti,ab,kw OR 'cheilitis':ti,ab,kw OR 'actinic cheilosis':ti,ab,kw OR 'potentially malignant':ti,ab,kw) AND ('mouth':ti,ab,kw OR 'mouths':ti,ab,kw OR 'oral':ti,ab,kw OR 'orals':ti,ab,kw OR 'lip':ti,ab,kw OR 'lips':ti,ab,kw OR 'tongue':ti,ab,kw OR 'buccal':ti,ab,kw OR 'palate':ti,ab,kw OR 'palates':ti,ab,kw OR 'cheek mucosa':ti,ab,kw OR 'alveolar':ti,ab,kw OR 'gingiva':ti,ab,kw OR 'gengivas':ti,ab,kw OR 'gum':ti,ab,kw OR 'gums':ti,ab,kw OR 'interdental papilla':ti,ab,kw OR 'interdental papillae':ti,ab,kw OR 'commissure':ti,ab,kw OR 'maxillary tuberosity':ti,ab,kw OR 'uvula':ti,ab,kw OR 'uvular':ti,ab,kw) AND [2017-2024]/py | | **1534** |
| Scopus | ("prevalence" OR "prevalences" OR "frequency" OR "frequencies" OR "epidemiology" OR "epidemiologic" OR "epidemiological" OR "occurrence" OR "occurrences" OR "incidence" OR "incidences") AND ("precancerous" OR "pre-cancer" OR "precancer" OR "precursor lesion" OR "precursor lesions" OR premalignan* OR pre malignan* OR "leukoplakia" OR "leukoplakias" OR "leukoplakic" OR "keratosis" OR "keratoses" OR "leukokeratosis" OR "leukokeratosis" OR "erythroplasia" OR "erythroplasias" OR "erythroplakia" OR "erythroplakias" OR "Carcinoma in Situ" OR "in situ carcinoma" OR "carcinomas in situ" OR "in situ carcinomas" OR "preinvasive carcinoma" OR "preinvasive carcinomas" OR "pre-invasive carcinoma" OR "pre-invasive carcinomas" OR "intraepithelial carcinoma" OR "intraepithelial carcinomas" OR "intraepithelial neoplasm" OR "intraepithelial neoplasms" OR "intraepithelial neoplasia" OR "intraepithelial neoplasias" OR "intraepithelial câncer" OR "oral submucous fibrosis" OR "cheilitis" OR "actinic cheilosis" OR "potentially malignant") AND ("mouth" OR "mouths" OR "oral" OR "orals" OR "lip" OR "lips" OR "tongue" OR "buccal" OR "palate" OR "palates" OR "cheek mucosa" OR "alveolar" OR "gingiva" OR "gengivas" OR "gum" OR "gums" OR "interdental papilla" OR "interdental papillae" OR "commissure" OR "maxillary tuberosity" OR "uvula" OR "uvular") | | **754** |
| Web of Science | ("prevalence" OR "prevalences" OR "frequency" OR "frequencies" OR "epidemiology" OR "epidemiologic" OR "epidemiological" OR "occurrence" OR "occurrences" OR "incidence" OR "incidences") AND ("precancerous" OR "pre-cancer" OR "precancer" OR "precursor lesion" OR "precursor lesions" OR premalignan* OR pre malignan* OR "leukoplakia" OR "leukoplakias" OR "leukoplakic" OR "keratosis" OR "keratoses" OR "leukokeratosis" OR "leukokeratosis" OR "erythroplasia" OR "erythroplasias" OR "erythroplakia" OR "erythroplakias" OR "Carcinoma in Situ" OR "in situ carcinoma" OR "carcinomas in situ" OR "in situ carcinomas" OR "preinvasive carcinoma" OR "preinvasive carcinomas" OR "pre-invasive carcinoma" OR "pre-invasive carcinomas" OR "intraepithelial carcinoma" OR "intraepithelial carcinomas" OR "intraepithelial neoplasm" OR "intraepithelial neoplasms" OR "intraepithelial neoplasia" OR "intraepithelial neoplasias" OR "intraepithelial câncer" OR "oral submucous fibrosis" OR "cheilitis" OR "actinic cheilosis" OR "potentially malignant") AND ("mouth" OR "mouths" OR "oral" OR "orals" OR "lip" OR "lips" OR "tongue" OR "buccal" OR "palate" OR "palates" OR "cheek mucosa" OR "alveolar" OR "gingiva" OR "gengivas" OR "gum" OR "gums" OR "interdental papilla" OR "interdental papillae" OR "commissure" OR "maxillary tuberosity" OR "uvula" OR "uvular") filtro 2017 | | **1034** |
| LILACS/BBO | ("prevalence" OR "prevalences" OR "frequency" OR "frequencies" OR "epidemiology" OR "epidemiologic" OR "epidemiological" OR "occurrence" OR "occurrences" OR "incidence" OR "incidences" OR "prevalência" OR "prevalências" OR "frequência" OR "frequências" OR "epidemiologia" OR "epidemiológica" OR "epidemiológico" OR "ocorrência" OR "ocorrências" OR "incidência" OR "incidências" OR "frecuencia" OR "frecuencias" OR "ocurrencia" OR "ocurrencias") AND ("precancerous" OR "pre-cancer" OR "precancer" OR "precursor lesion" OR "precursor lesions" OR premalignan* OR pre malignan* OR "leukoplakia" OR "leukoplakias" OR "leukoplakic" OR "keratosis" OR "keratoses" OR "leukokeratosis" OR "leukokeratosis" OR "erythroplasia" OR "erythroplasias" OR "erythroplakia" OR "erythroplakias" OR "Carcinoma in Situ" OR "in situ carcinoma" OR "carcinomas in situ" OR "in situ carcinomas" OR "preinvasive carcinoma" OR "preinvasive carcinomas" OR "pre-invasive carcinoma" OR "pre-invasive carcinomas" OR "intraepithelial carcinoma" OR "intraepithelial carcinomas" OR "intraepithelial neoplasm" OR "intraepithelial neoplasms" OR "intraepithelial neoplasia" OR "intraepithelial neoplasias" OR "intraepithelial câncer" OR "oral submucous fibrosis" OR "cheilitis" OR "actinic cheilosis" OR "potentially malignant" OR "pré-canceroso" OR "lesão precursora" OR "lesões precursoras" OR pré-maligno* OR pré maligno* OR "pré-malignidade" OR "pré-malignidades" OR "leucoplasia" OR "leucoplasias" OR "queratose" OR "queratoses" OR "leucoqueratose" OR "leucoqueratoses" OR "eritroplasia" OR "eritroplasias" OR "carcinoma pré-invasivo" OR "carcinomas pré-invasivos" OR "carcinoma intraepitelial" OR "carcinomas intraepiteliais" OR "neoplasia intraepitelial" OR "neoplasias intraepiteliais" OR "câncer intraepitelial" OR "fibrose submucosa oral" OR "queilite" OR "queilose actínica" OR "potencialmente malignas" OR "precanceroso" OR "lesiones precursoras" OR premaligno* OR "premalignidad" OR "premalignidades" OR "queratosis" OR "leucoqueratosis" OR "eritroplaquia" OR "carcinoma preinvasivo" OR "carcinomas preinvasivos" OR "carcinoma preinvasivo" OR "carcinomas preinvasivos" OR "carcinoma intraepitelial" OR "carcinomas intraepiteliales" OR "neoplasia intraepitelial" OR "neoplasias intraepiteliales" OR "cáncer intraepitelial" OR "fibrosis submucosa oral" OR "queilitis" OR "queilosis actínica" OR "potencialmente malignas") AND ("mouth" OR "mouths" OR "oral" OR "orals" OR "lip" OR "lips" OR "tongue" OR "buccal" OR "palate" OR "palates" OR "cheek mucosa" OR "alveolar" OR "gingiva" OR "gengivas" OR "gum" OR "gums" OR "interdental papilla" OR "interdental papillae" OR "commissure" OR "maxillary tuberosity" OR "uvula" OR "uvular" OR "Boca" OR "Bocas" OR "Orais" OR "Lábio" OR "Lábios" OR "Língua" OR "bucal" OR "palato" OR "palatos" OR "mucosa da bochecha" OR "gengiva" OR "gengivas" OR "papila interdental" OR "papilas interdentais" OR "comissura" OR "tuberosidade maxilar" OR "lengua" OR "paladar" OR "paladares" OR "mucosa de la mejilla" OR "alveolar" OR "encía" OR "encías" OR "papilas interdentales" OR "comisura" OR "tuberosidad maxilar") | | **1803** |
| EBSCO | ("prevalence" OR "prevalences" OR "frequency" OR "frequencies" OR "epidemiology" OR "epidemiologic" OR "epidemiological" OR "occurrence" OR "occurrences" OR "incidence" OR "incidences") AND ("precancerous" OR "pre-cancer" OR "precancer" OR "precursor lesion" OR "precursor lesions" OR premalignan* OR pre malignan* OR "leukoplakia" OR "leukoplakias" OR "leukoplakic" OR "keratosis" OR "keratoses" OR "leukokeratosis" OR "leukokeratosis" OR "erythroplasia" OR "erythroplasias" OR "erythroplakia" OR "erythroplakias" OR "Carcinoma in Situ" OR "in situ carcinoma" OR "carcinomas in situ" OR "in situ carcinomas" OR "preinvasive carcinoma" OR "preinvasive carcinomas" OR "pre-invasive carcinoma" OR "pre-invasive carcinomas" OR "intraepithelial carcinoma" OR "intraepithelial carcinomas" OR "intraepithelial neoplasm" OR "intraepithelial neoplasms" OR "intraepithelial neoplasia" OR "intraepithelial neoplasias" OR "intraepithelial câncer" OR "oral submucous fibrosis" OR "cheilitis" OR "actinic cheilosis" OR "potentially malignant") AND ("mouth" OR "mouths" OR "oral" OR "orals" OR "lip" OR "lips" OR "tongue" OR "buccal" OR "palate" OR "palates" OR "cheek mucosa" OR "alveolar" OR "gingiva" OR "gengivas" OR "gum" OR "gums" OR "interdental papilla" OR "interdental papillae" OR "commissure" OR "maxillary tuberosity" OR "uvula" OR "uvular") | | **417** |
| Google Scholar | ("prevalence" OR "epidemiology" OR "incidence" OR "prevalência" OR "epidemiologia" OR "incidência") AND ("precancerous" OR "pre-cancer" OR "precancer" OR "precursor lesion" OR "precursor lesions" OR premalignan* OR pre malignan* OR "Carcinoma in Situ" OR "potentially malignant" OR "pré-canceroso" OR "lesão precursora" OR "lesões precursoras" OR pré-maligno* OR pré maligno* OR "pré-malignidade" OR "pré-malignidades" OR "potencialmente malignas" OR "precanceroso" OR "lesiones precursoras" OR premaligno* OR "premalignidad" OR "premalignidades" OR "potencialmente malignas") AND ("mouth" OR "mouths" OR "oral" OR "orals" OR "lip" OR "lips" OR "tongue" OR "buccal" OR "palate" OR "palates" OR "cheek mucosa" OR "alveolar" OR "gingiva" OR "gengivas" OR "gum" OR "gums" OR "interdental papilla" OR "interdental papillae" OR "commissure" OR "maxillary tuberosity" OR "uvula" OR "uvular" OR "Boca" OR "Bocas" OR "Orais" OR "Lábio" OR "Lábios" OR "Língua" OR "bucal" OR "palato" OR "palatos" OR "mucosa da bochecha" OR "gengiva" OR "gengivas" OR "papila interdental" OR "papilas interdentais" OR "comissura" OR "tuberosidade maxilar" OR "lengua" OR "paladar" OR "paladares" OR "mucosa de la mejilla" OR "alveolar" OR "encía" OR "encías" OR "papilas interdentales" OR "comisura" OR "tuberosidad maxilar") | | **only the first 200 papers were screened** |
| Proquest | *Advanced Search – comand line*  ABSTRACT("prevalence" OR "prevalences" OR "frequency" OR "frequencies" OR "epidemiology" OR "epidemiologic" OR "epidemiological" OR "occurrence" OR "occurrences" OR "incidence" OR "incidences") AND ABSTRACT("precancerous" OR "pre-cancer" OR "precancer" OR "precursor lesion" OR "precursor lesions" OR "leukoplakia" OR "leukoplakias" OR "leukoplakic" OR "keratosis" OR "keratoses" OR "leukokeratosis" OR "leukokeratosis" OR "erythroplasia" OR "erythroplasias" OR "erythroplakia" OR "erythroplakias" OR "Carcinoma in Situ" OR "in situ carcinoma" OR "carcinomas in situ" OR "in situ carcinomas" OR "preinvasive carcinoma" OR "preinvasive carcinomas" OR "pre-invasive carcinoma" OR "pre-invasive carcinomas" OR "intraepithelial carcinoma" OR "intraepithelial carcinomas" OR "intraepithelial neoplasm" OR "intraepithelial neoplasms" OR "intraepithelial neoplasia" OR "intraepithelial neoplasias" OR "intraepithelial câncer" OR "oral submucous fibrosis" OR "cheilitis" OR "actinic cheilosis" OR "potentially malignant") AND ABSTRACT("mouth" OR "mouths" OR "oral" OR "orals" OR "lip" OR "lips" OR "tongue" OR "buccal" OR "palate" OR "palates" OR "cheek mucosa" OR "alveolar" OR "gingiva" OR "gengivas" OR "gum" OR "gums" OR "interdental papilla" OR "interdental papillae" OR "commissure" OR "maxillary tuberosity" OR "uvula" OR "uvular") | | **622** |
| TOTAL | *Without duplicate removal* | | **8,070** |
|  | *After duplicate removal (automatic n=3078 + manual n=88)* | | **4,904** |

*All searches were conducted with a filter set to the timeframe 2017-2025, taking into account that this is an update from previous work.
