# Supplementary material for: Global Prevalence of Oral Potentially Malignant Disorders: An Updated Systematic Review and Meta‐Analysis
Source: J Oral Pathol Med. 2026 Apr 28;55(7):747–54. doi: 10.1111/jop.70146 (PMC13429371; doi:10.1111/jop.70146)
Supplement: Supplementary file 2 — Appendix S2: Reasons for exclusion of studies following full‐text screening. [file JOP-55-747-s001.docx]

| **Appendix S2.** Reasons for exclusion of studies following full-text screening (n=330). | | |
| --- | --- | --- |
|  | **Excluded Studies Following Full-Text Screening (n=330)** | **Criteria** |
|  | A clinical study of incidence and risk factors associated with oral premalignant lesions | **4** |
|  | A clinico-demographic evaluation of patients with oral submucous fibrosis: a cross-sectional study | **4** |
|  | A novel mobile health approach to early diagnosis of oral cancer | **3** |
|  | A prospective study on diagnostic accuracy of technology-enabled early detection of oral cancer and epidemiology of tobacco and other substances use in rural India | **2** |
|  | A simple screening program for oral cancer in a defined geographic area in southern India: a community-based cross-sectional study | **2** |
|  | A study of precancerous lesions for oral cancer in jamnagar city | **3** |
|  | A study of risk factors associated with the presence of oral potentially malignant disorders: a community-based study from northeastern Thailand | **3** |
|  | A study of the natural history of oral preleukoplakia | **3** |
|  | A study on oral mucosal changes among tobacco users | **2** |
|  | A longitudinal study on oral potentially malignant and malignant lesions in a tertiary care teaching hospital | **3** |
|  | Actinic cheilitis in rural workers: prevalence and associated factors | **8** |
|  | Actinic cheilitis prevalence and risk factors: a cross-sectional, multicentre study in a population aged 45 years and over in north-west spain | **3** |
|  | Análisis de los resultados nacionales obtenidos por grupos estatales de investigación del el cáncer ruso. Programa de cribado de enfermedades de la mucosa oral y borde bermellón | **3** |
|  | Analysis of false-negatives in exfoliative cytology in oral potentially malignant disorders: a retrospective cohort study | **8** |
|  | Analysis of the results obtained by national research groups with the russian state cancer program for the screening of diseases of the oral mucosa and the vermillion border | **3** |
|  | Analyzing the frequency of premalignant lesions and oral malignancy in indian subjects attending outpatient department from the low socioeconomic group | **3** |
|  | Assessment of betel quid habits and risk of precancerous oral lesions among paniya tribes of wayanad, india-a cross-sectional study | **5** |
|  | Assessment of incidence of oral submucous fibrosis patients - an institutional study | **4** |
|  | Assessment of oral health care needs among fishermen living in north chennai, india - a cross-sectional study | **3** |
|  | Assessment of oral health status and tobacco-related habits among the employees of north-west karnataka road transport corporation (nwkrtc), belagavi city, india - a | **3** |
|  | Assessment of oral health status and treatment needs among sugali tribe in telangana region - a cross-sectional study | **3** |
|  | Assessment of oral health status and treatment needs of tribal population in yelagiri hlls, vellore district, tamil nadu - a cross-sectional study | **3** |
|  | Assessment of oral lesions and its association with smoking habits in paniya tribes of India | **5** |
|  | Assessment of pattern of oral prosthetic treatment and prevalence of oral diseases in edentulous patients in north Indian population: a cross-sectional study | **3** |
|  | Assessment of periodontal status in patients with oral lesions-a retrospective study in south Indian population | **4** |
|  | Assessment of potentially malignant disorders among men and women in south bihar population | **3** |
|  | Assessment of various pre-malignant oral lesions and associated factors at nmch patna | **4** |
|  | Association between oral leukoplakia smoking and alcohol habits in patients | **3** |
|  | Association between oral mucosal lesions and crack and cocaine addiction in men: a cross-sectional study | **3** |
|  | Association between oral potentially malignant disorders with age, gender, site and habits in western part of Maharashtra population | **4** |
|  | Association of clinical variants of leukoplakia with different types of tobacco - an institution based retrospective study | **8** |
|  | Association of dsm-5 betel-quid use disorder with oral potentially malignant disorder in 6 betel-quid endemic asian populations | **3** |
|  | Association of oral premalignant lesions with the consumption of tobacco | **2** |
|  | Betel nut chewing habits and risk of precancerous oral lesions among paniya tribes of Wayanad, india: a cross-sectional study | **5** |
|  | Betel‐quid and oral submucous fibrosis: a cross‐sectional study in hunan province, china | **3** |
|  | Biópsias orais numa população portuguesa: estudo clinicopatológico dos últimos 20 anos numa clínica universitária | **5** |
|  | Burden of oral precancer and cancer among an indigenous tribal population of south India - an evaluative study | **3** |
|  | Burden of tobacco and alcohol consumption and its association with periodontal disease, potentially malignant lesions and quality of life among bus drivers in Lagos state, Nigeria | **5** |
|  | Caracterização clínica e histológica de uma população diagnosticada com líquen plano oral numa clínica universitária portuguesa nos últimos 22 anos | **7** |
|  | Caracterização das lesões de tecidos moles e duros nos pacientes da clínica dentária universitária: um estudo retrospetivo | **5** |
|  | Caracterización clínico e histopatológica de la leucoplasia bucal | **4** |
|  | Changes in prevalence of precancerous oral submucous fibrosis from 1996 to 2013 in Taiwan: a nationwide population-based retrospective study | **3** |
|  | Characterization of epithelial oral dysplasia in non-smokers: first steps towards precision medicine | **4** |
|  | Characterization of patients with epithelial dysplasia and carcinoma in situ in the oral cavity, 2000 to 2014 | **4** |
|  | Chewing and smoking habits in relation to precancer and oral cancer | **3** |
|  | Clinical analysis for oral mucosal disease in 21 972 cases | **7** |
|  | Clinical and histological prognostic factors of malignant transformation in a large series of oral potentially malignant disorders (OPMDs) | **5** |
|  | Clinical and histological prognostic factors of recurrence and malignant transformation in a large series of oral potentially malignant disorders | **4** |
|  | Clinical characteristics of oral lichen planus in patients visiting a private dental institution-a retrospective study | **6** |
|  | Clinical epidemiological evaluation of the oral premalignant lesions in the population of the Montalvo in Ambato, Ecuador | **3** |
|  | Clinical profile and prevalence of oral mucosal lesions in tobacco users-a prospective study from Jammu, India | **2** |
|  | Clinico-epidemiological study of oral potentially malignant and malignant lesions in a tertiary care centre of odisha- a five year longitudinal study | **3** |
|  | Clinicopathological evaluation of oral leukoplakia: a single-center study of 676 cases in japan | **4** |
|  | Clinicopathological evaluation of oral submucous fibrosis - a retrospective, single institute study | **4** |
|  | Clinicopathological evaluation of tobacco-related oral mucosal lesions | **2** |
|  | Community-based early detection of oral precancerous lesion, accessibility and utilization of oral healthcare services among irular tribes residing at union territory of pondicherry | **3** |
|  | Community-based oral cancer screening for women of remote & rural blocks of the chikkaballapur district, karnataka, india | **8** |
|  | Community-based screening of oral cancer in selected districts of nepal: a cross-sectional study | **3** |
|  | Comparing the use of tobacco among fishermen and non-fishermen population of pondicherry, india | **5** |
|  | Comparison of the accuracy of diagnoses of oral potentially malignant disorders with dysplasia by a general dental clinician and a specialist using the taiwanese nationwide oral mucosal screening program | **7** |
|  | Comparison of prevalence of leukoplakia in males and females among chennai population | **3** |
|  | Compatibility of clinical and histopathological diagnosis of oral lesions in iranian patients | **7** |
|  | Comportamiento del programa de detección precoz del cáncer bucal en la clínica artemio mastrapa. Holguín, 2015 | **3** |
|  | Condição de saúde bucal de pacientes internados em um hospital público brasileiro | **3** |
|  | Congruencia diagnóstica clínica-histopatológica de lesiones en cavidad oral, en la faculta de odontología, universidad de costa rica, en el período 2016-2019 | **1** |
|  | Correlation between clinical and histopathologic diagnosis of oral potentially malignant disorder and oral squamous cell carcinoma | **4** |
|  | Correlation of oral mucosal lesions with various habits and histopathological findings | **1** |
|  | Countermeasure and opportunistic screening systems for oral cancer | **7** |
|  | Cytopathological study of oral lesions in tertiary care hospital in western part of india | **3** |
|  | Demographic analysis of prevalence of oral lesions in patients with deleterious habits– an institutional based descriptive observational study | **6** |
|  | Detection of potentially malignant lesions and squamous cell carcinomas in consultation of surgical dentistry | **3** |
|  | Determinants of tobacco use and prevalence of oral precancerous lesions in cab drivers in bengaluru city, india | **3** |
|  | Determination of the prevalence of oral potentially malignant disorders among tobacco users in a subset of kolkata population | **2** |
|  | Distribution and prevalence of oral mucosal lesions in residents of old age homes in delhi, india | **3** |
|  | Early detection of oral cancer and prevalence of tobacco use among the general population in hassan district | **5** |
|  | Effect of smokeless tobacco (st) and areca nut chewing among adults in gulyana village pakistan | **3** |
|  | Epidemiological hazards of tobacco and its manifestations in oral health of a screened population in northern india | **4** |
|  | Epidemiological profile and clinical characteristics of oral potentially malignant disorders and oral squamous cell carcinoma: a pilot study in bidar and gulbarga districts, karnataka, india | **4** |
|  | Epidemiological profile of tobacco users attending a tobacco cessation centre at puducherry, india | **2** |
|  | Epidemiological situation of pre-cancer diseases of the oral mucous in ukraine | **6** |
|  | Epidemiological studies of the prevalence of the oral mucosa and the red lip border lesions | **6** |
|  | Epidemiology of quid usage and its possible association with the occurrence of oral mucosal lesions | **2** |
|  | Epidemiology profile and outcomes of oral potentially malignant disorders among different geographic regions of taiwan: a retrospective observational nationwide population database study | **7** |
|  | Estudo retrospetivo da concordância entre o diagnóstico clínico e anatomopatológico em pacientes da clínica universitária egas moniz | **5** |
|  | Evaluación clínico-epidemiológica de trastornos bucales potencialmente malignos en adultos de montalvo en ambato, ecuador | **3** |
|  | Evaluation of demographic profile, risk factors and clinical presentation of oral submucous fibrosis in patients attending ent opd | **3** |
|  | Evaluation of lip diseases in peddlers on two beaches of guarujá/brazil: an observational study | **3** |
|  | Evaluation of oral lesions and dental health in hiv-positive saudi patients | **3** |
|  | Evaluation of oral mucosal lesions in iranian smokers and non-smokers | **2** |
|  | Evaluation of oral pathologic lesions in elderly patients in isfahan, iran, 1989-2018 years | **8** |
|  | Evaluation of the prevalence of oral mucosal lesions in a population of eastern coast of south india | **3** |
|  | Exploring the prevalence and risk factors of benign and premalignant oral lesions in an adult population from northern spain: a pilot study | **6** |
|  | Factores asociados a lesiones bucales premalignas en pacientes mayores de 60 años de un consultorio médico | **3** |
|  | Factors associated with oral cancerous and precancerous lesions in an underserved community: a cross-sectional study | **3** |
|  | Factors influencing tobacco use and associated premalignant conditions among police personnel | **5** |
|  | Follow-up study of veterans with white and red oral mucosal lesions at veterans affairs dental clinics | **4** |
|  | Frecuencia de lesiones y condiciones orales en pacientes mayores de 18 años en una clínica de atención primaria en méxico | **3** |
|  | Frequency of oral mucosal lesions among tobacco chewers: a cross-sectional survey | **3** |
|  | Frequency of oral mucosal lesions and awareness of these lesions in patients attending oral and maxillofacial clinic in sari dental school, iran | **7** |
|  | Frequency of pathologic changes in the oral cavity in patients subjected to long-term pharmacologic immunosuppressive therapy after kidney, liver, and hematopoietic cell transplantation | **8** |
|  | Frequency of systemic diseases and oral lesions among the institutionalized elderly subjects in the northeast of iran | **3** |
|  | Frequency, distribution, and risk factors of oral mucosal lesions in a colombian population: cross-sectional study | **3** |
|  | From plate to palette: dietary patterns and their role in mucosal lesions among north indian communities: a cross-sectional study | **3** |
|  | High prevalence of lifestyle factors attributable for oral cancer, and of oral potentially malignant disorders in rural sri lanka | **3** |
|  | High prevalence of oral potentially malignant disorders and risk factors in a semi-urban brazilian city: a population-based cross-sectional study | **3** |
|  | Histopathological evaluation of white lesions– an institutional observational study | **4** |
|  | Histopathological findings of oral and maxillofacial biopsies from a sample of yemeni patients | **7** |
|  | Histopathological spectrum of lesions of oral cavity and oropharynx in a tertiary health centre | **1** |
|  | Histopathological study of lesions of oral cavity and oropharynx in rajarajeswari medical college & hospital | **5** |
|  | Impact of oral potentially malignant disorder subtypes on all‐cause and cause‐specific mortality in males | **4** |
|  | Impact of smoking and smokeless tobacco on oral cavity | **2** |
|  | Impact of the covid-19 pandemic on public university laboratories of oral and maxillofacial pathology: a brazilian multicenter study | **1** |
|  | Impact of tobacco chewing on oral health: a hospital-based study in eastern, india | **3** |
|  | Implication for second primary cancer from visible oral and oropharyngeal premalignant lesions in betel-nut chewing related oral cancer | **2** |
|  | Incidence of oral mucosal diseases among croatian patients at university hospital centre zagreb | **5** |
|  | Incidence of oral white lesions in patients attending a private dental hospital | **3** |
|  | Incidence of potentially malignant oral disorders in patients attending a private dental hospital | **3** |
|  | Incidence of tobacco related white lesions in patients | **3** |
|  | Incidence rates of oral cancer and natural history of oral precancerous lesions in a 10‐year follow‐up study of indian villagers | **3** |
|  | Incidencia de patologías bucomaxilares en un período de aislamiento social preventivo y obligatorio de la pandemia covid-19 | **4** |
|  | Influence of risk habits on demographic factors and its impact on oral submucous fibrosis | **3** |
|  | Influence of tobacco chewing on oral health: a hospital-based cross-sectional study in odisha | **3** |
|  | Investigating the association between tobacco use and oral health among security guards at a tertiary healthcare centre in new delhi: a cross-sectional study | **3** |
|  | Investigation of biopsied non-plaque-induced gingival lesions in a turkish population: a 5-year retrospective study | **7** |
|  | Is periodontitis independently associated with potentially malignant disorders of the oral cavity? | **3** |
|  | Is the use of tobacco products, especially electronic nicotine delivery systems (ends), associated with the incidence of oral health outcomes among us adults? | **5** |
|  | Lesiones cancerígenas y precancerosas bucales. Factores de riesgos asociados. Policlínico bernardo posse. Año 2017 | **3** |
|  | Lesiones premalignas y malignas en el complejo bucal en la palma, pinar del río | **4** |
|  | Lesões leucoplásicas nos utentes da clínica dentária universitária : um estudo retrospetivo de 17 anos | **5** |
|  | Leukoplakia and erythroplakia in youngers versus older individuals: a clinicopathological retrospective study | **4** |
|  | Malignant transformation of actinic cheilitis: a decade-long retrospective study in southern brazil | **4** |
|  | Mobile health approach for follow-up care in early oral cancer screening program | **5** |
|  | Mouth self-examination (mse) as a screening tool for oral potentially malignant disorders among a high-risk indigenous population with a low socioeconomic status | **5** |
|  | Needs for gerodontological treatment in the elderly living in lower silesia | **3** |
|  | Nonsmoked tobacco addiction playing a major role in oral mucosal changes in northern indian population over smoked and alcohol use | **5** |
|  | Occurrence of leukoplakia and some other oral white lesions among 20 333 adult swedish people | **3** |
|  | Opportunistic screening for detection and socio-epidemiological risk assessment of oral cancer patients in rural jodhpur, india | **4** |
|  | Oral and dental status of bulgarian patients- a 5-year study | **3** |
|  | Oral and maxillofacial lesions in older individuals and associated factors: a retrospective analysis of cases retrieved in two different services | **7** |
|  | Oral and oropharyngeal mucosal lesions: clinical-epidemiological study of patients attended at a reference center for infectious diseases | **3** |
|  | Oral cancer screening for high-risk individuals in the primary healthcare setting using an active approach | **2** |
|  | Oral health conditions and unmet need among chinese male adults: a national oral health survey | **3** |
|  | Oral health disparities among privileged and underprivileged tribes of south india - a study on precancerous oral lesions prevalence | **5** |
|  | Oral health inequalities among tobacco users in the tea plantation workers, nilgiri hills, tamilnadu, india | **5** |
|  | Oral health of an indigenous population in northeastern brazil: a cross-sectional study of the fulni-ô ethnic group | **3** |
|  | Oral health of older people: tracking soft tissue injuries for the prevention of oral cancer | **3** |
|  | Oral health status among lorry drivers in hyderabad city - a cross sectional study | **6** |
|  | Oral health status and behaviour among kanikkaran tribes of mundandhurai hills in tirunelveli district, tamil nadu | **3** |
|  | Oral health status and oral health risks among tribes in tamil nadu, india: an epidemiological study | **3** |
|  | Oral health status and treatment needs among adult tribal population in attappadi, kerala | **3** |
|  | Oral health status and treatment needs among multiple factory workers, tumkur city u a cross sectional study | **3** |
|  | Oral health status and treatment needs of chromium mine workers in india | **3** |
|  | Oral health status and treatment needs of santhal tribe in ponda village,bokaro district ,jharkhanda cross-sectional study | **3** |
|  | Oral lesions associated with tobacco smoking in adolescents/adults dwellers in lagos, nigeria | **5** |
|  | Oral leukoplakia and erythroplakia in young patients: a southern brazilian multicenter study | **4** |
|  | Oral leukoplakia. Ii. Results of a year-long polyclinical pilot study | **6** |
|  | Oral leukoplakia-epidemiological survey and histochemical analysis of 107 cases in brazil | **4** |
|  | Oral malignancies and tobacco related habits among aranadar tribals in kerala, india: a population based study | **5** |
|  | Oral mucosal lesions and conditions in patients with dermatologic diseases: how common is it? | **3** |
|  | Oral mucosal lesions and risk factors in elderly dental patients | **3** |
|  | Oral mucosal lesions in electronic cigarettes consumers versus former smokers | **4** |
|  | Oral mucosal lesions in patients attending dermatology outpatient department of a tertiary care center in kathmandu: a descriptive cross-sectional study | **3** |
|  | Oral pathology in a population observed within an oral cancer screening developed in portugal | **3** |
|  | Oral potentially malignant disorders among patients attending the department of oral medicine and radiology of a tertiary care dental hospital: a descriptive cross-sectional study | **3** |
|  | Oral potentially malignant disorders and oral cancer in nepalese dental patients | **5** |
|  | Oral potentially malignant disorders and squamous cell carcinoma at the tongue: clinicopathological analysis in a thai population | **8** |
|  | Oral potentially malignant disorders clinical database at the oral and maxillofacial surgery department, montreal general hospital–mcgill university health centre | **4** |
|  | Oral potentially malignant disorders: clinical-pathological study of 684 cases diagnosed in a brazilian population | **4** |
|  | Oral pre-malignant and malignant lesion detection among Indonesians: the prevalence and risk factors | **5** |
|  | Oral squamous cell carcinomas and oral potentially malignant disorders: a Latin American study | **3** |
|  | Oral submucous fibrosis: study of 1000 cases from central India | **3** |
|  | Pacientes geriátricos na clínica de medicina e cirurgia oral da fmdul | **5** |
|  | Pan masala habits and risk of oral precancer: a cross-sectional survey in 0.45 million people of north India | **3** |
|  | Pattern of distribution of biopsy confirmed oral and maxillofacial lesions in adult and geriatric age groups of central Kerala population-an institutional retrospective study of 11 years | **7** |
|  | Patterns and distribution of tobacco use and its association with oral precancerous lesions among the tribes of Odisha | **3** |
|  | Patterns of tobacco use and its relation to oral precancers and cancers among individuals visiting a tertiary hospital in south India | **2** |
|  | Perfil dos pacientes atendidos no curso de odontologia do sertão de Pernambuco: perfil dos pacientes atendidos no sertão | **3** |
|  | Perfil epidemiológico de um serviço de medicina bucal: estudo retrospectivo de 17 anos | **5** |
|  | Perfil epidemiológico e social de indivíduos com desordens orais potencialmente malignas | **3** |
|  | Pesquisaje de lesiones premalignas y malignas en la cavidad bucal | **3** |
|  | Potencial de transformación maligna de las lesiones blanquecinas bucales | **4** |
|  | Potentially malignant disorders revisited-the lichenoid lesion/proliferative verrucous leukoplakia conundrum | **4** |
|  | Predictors of dysplasia in oral submucous fibrosis: a retrospective observational study | **4** |
|  | Prevalence and biosocial determinants of potentially malignant disorders of oral soft tissue in slum population of western Uttar Pradesh | **3** |
|  | Prevalence and determinants of oral potentially malignant disorders in rural areas of south India | **3** |
|  | Prevalence and determinants of oral potentially malignant lesions using mobile health in a rural block, northeast India | **3** |
|  | Prevalence and distribution of oral mucosal lesions associated with tobacco use in patients visiting a dental school in Ajman | **3** |
|  | Prevalence and distribution of oral mucosal lesions by sex and age categories: a retrospective study of patients attending Lebanese school of dentistry | **3** |
|  | Prevalence and distribution of oral mucosal lesions in Jammu, Jammu & Kashmir: a prospective study | **3** |
|  | Prevalence and factors associated with oral potentially malignant disorders and oral squamous cell carcinoma: an institutional study | **3** |
|  | Prevalence and management of oral submucous fibrosis and its implications on prosthodontic treatment: a retrospective study | **3** |
|  | Prevalence and organization problems of medical care to patients with precanceral oral cavity mucosa diseases (on the Tomsk region example) | **7** |
|  | Prevalence and pattern of tobacco-associated oral lesion among migrant construction workers in Chennai: a cross-sectional study | **3** |
|  | Prevalence and patterns of oral mucosal lesions among geriatric patients in India: a retrospective study | **3** |
|  | Prevalence and risk factors for oral potentially malignant disorders in Indian population | **3** |
|  | Prevalence and risk factors of oral mucosal lesions: a retrospective study of patients attending oral diagnosis department of siohs Karachi, Pakistan | **3** |
|  | Prevalence and risk factors of oral potentially malignant disorders in Indonesia: a cross-sectional study undertaken in 5 provinces | **3** |
|  | Prevalence and risk factors of potentially malignant disorders of the mucosa in the general population mucosa lesions a general health problem? | **3** |
|  | Prevalence and risk indicators of oral mucosal lesions in adult population visiting primary health centers and community health centers in Kodagu district | **3** |
|  | Prevalence and treatment for cheilitis - a retrospective study | **1** |
|  | Prevalence of actinic cheilitis and lip squamous cell carcinoma among lip lesions | **5** |
|  | Prevalence of and factors associated with actinic cheilitis in extractive mining workers | **8** |
|  | Prevalence of gul use, its predictors and association with oral potentially malignant disorders and oral cancer development in the users of Noida, India: a cross-sectional study | **5** |
|  | Prevalence of habit-related oral lesions in Kolkata and the surrounding districts | **3** |
|  | Prevalence of homogenous and non-homogenous leukoplakia in a private dental hospital | **3** |
|  | Prevalence of lesions and oral mucosal normal variations in an elderly population in Costa Rica | **3** |
|  | Prevalence of leukoplakia in relation to tobacco habits in southern Orissa | **6** |
|  | Prevalence of leukoplakia, oral lichen planus and tobacco pouch keratosis -among patients visiting a private dental institution | **6** |
|  | Prevalence of lip lesions in patients visiting a dental hospital | **4** |
|  | Prevalence of most common tongue lesions among a group of UAE population: retrospective study | **8** |
|  | Prevalence of oral and maxillofacial diseases in an Italian population: retrospective study on clinical and pathological features | **5** |
|  | Prevalence of oral cancer among patients using different forms of tobacco - a retrospective study | **6** |
|  | Prevalence of oral cancer and oral epithelial dysplasia among north Indian population: a retrospective institutional study | **7** |
|  | Prevalence of oral cancer, oral potentially malignant disorders and other oral mucosal lesions in Cambodia | **8** |
|  | Prevalence of oral lesions diagnosed at a pathology institute: a four-year analysis | **7** |
|  | Prevalence of oral lesions diagnosed at the ULBRA canoas of dental diagnosis service | **7** |
|  | Prevalence of oral lesions in relation to tobacco and alcohol habits using velscope - a retrospective study | **4** |
|  | Prevalence of oral lesions in tobacco and alcohol users | **5** |
|  | Prevalence of oral leukoplakia in oral cancer prevention in the state of Paraná Brazil between 1989 and 2017 | **5** |
|  | Prevalence of oral lichen planus and assessment of factors associated with it-a retrospective study | **3** |
|  | Prevalence of oral mucosal lesion in patients with tobacco related habits among Chidambaram population – a cross-sectional study | **3** |
|  | Prevalence of oral mucosal lesions among dental patients with mixed habits in Salem district - a study | **3** |
|  | Prevalence of oral mucosal lesions among elderly population in Chennai: a survey | **3** |
|  | Prevalence of oral mucosal lesions among smokeless tobacco usage: a cross-sectional study | **2** |
|  | Prevalence of oral mucosal lesions among tobacco consumers: a cross-sectional study | **2** |
|  | Prevalence of oral mucosal lesions among tobacco consumers: cross-sectional study | **2** |
|  | Prevalence of oral mucosal lesions among tobacco users - a cross-sectional study | **6** |
|  | Prevalence of oral mucosal lesions and relation to serum cotinine levels—findings from a cross- sectional study in south Africa | **3** |
|  | Prevalence of oral mucosal lesions in geriatric patients living in lower northern Thailand: a 10 years retrospective study | **3** |
|  | Prevalence of oral mucosal lesions in geriatric population of coastal Andhra Pradesh | **3** |
|  | Prevalence of oral mucosal lesions in Punjab, India | **3** |
|  | Prevalence of oral mucosal lesions in Savitha dental college: a retrospective study | **3** |
|  | Prevalence of oral mucosal lesions in young seniors in the Wroclaw region | **3** |
|  | Prevalence of oral mucosal lesions: a prospective study | **3** |
|  | Prevalence of oral mucosal normal variations and lesions in a middle-aged population: a northern Finland birth cohort 1966 study | **3** |
|  | Prevalence of oral potentially malignant and malignant lesions and tobacco use among the older adults attending a screening clinic in Noida (India): a cross-sectional study | **5** |
|  | Prevalence of oral potentially malignant disorders (opmd) in adults of western Maharashtra, India: a cross-sectional study | **3** |
|  | Prevalence of oral potentially malignant disorders among fishermen population in and around Pondicherry, south India - a cross sectional study | **3** |
|  | Prevalence of oral potentially malignant disorders among tobacco users in Kolkata: a hospital-based study | **3** |
|  | Prevalence of oral potentially malignant disorders and oral malignant lesions: a population-based study in a municipal town of southern Kerala | **5** |
|  | Prevalence of oral potentially malignant disorders associated with habits in Puducherry-a cross-sectional study | **3** |
|  | Prevalence of oral potentially malignant lesions, tobacco use, and effect of cessation strategies among solid waste management workers in northern India: a pre-post intervention study | **3** |
|  | Prevalence of oral precancerous lesions in tobacco and areca nut habituated patients in Barpeta district, assam, India: a cross-sectional study | **3** |
|  | Prevalence of oral premalignant lesions and conditions among the population of Kanpur city, India: a cross-sectional study | **3** |
|  | Prevalence of oral premalignant lesions and its risk factors among the adult population in Udupi taluk of coastal Karnataka, india | **3** |
|  | Prevalence of oral squamous cell carcinoma and oral potentially malignant lesions diagnosed in Santa Catarina state | **5** |
|  | Prevalence of oral submucous fibrosis among tobacco users reporting to a private dental college in Chennai: a retrospective study | **6** |
|  | Prevalence of oral submucous fibrosis in construction workers | **3** |
|  | Prevalence of oral submucous fibrosis in patients visiting dental college: a cross-sectional study | **3** |
|  | Prevalence of oral submucous fibrosis in the high natural radiation belt of Kerala, south India | **3** |
|  | Prevalence of oral submucous fibrosis linking with areca nut usage among Indians | **3** |
|  | Prevalence of oral submucous fibrosis with other oral potentially malignant disorders: a clinical retrospective study | **4** |
|  | Prevalence of oral submucous fibrosis, its correlation of clinical grading to various habit factors among patients of Bihar: a cross sectional study | **3** |
|  | Prevalence of oromucosal lesions in relation to tobacco habit among a western Maharashtra population | **3** |
|  | Prevalence of OSMF cases in various age groups correlating with habits and treatment outcomes in Saveetha dental college: a prevalence study | **3** |
|  | Prevalence of potentially malignant disorders in tobacco consuming population: a cross-sectional analysis | **3** |
|  | Prevalence of potentially malignant oral disorders among migrant labourers | **6** |
|  | Prevalence of precancerous lesions in an adult population | **3** |
|  | Prevalence of premalignant conditions and their transformation into oral cancers: a clinical study | **4** |
|  | Prevalence of premalignant lesions and oral cancer among tobacco-using tea plantation workers of nigiri hills, Tamil Nadu, India | **5** |
|  | Prevalence of recurrent aphthous stomatitis, oral submucosal fibrosis and oral leukoplakia in doctor/nurse and police officer population | **3** |
|  | Prevalence of smokeless tobacco use and oral pre-malignant lesions among heavy load truck drivers and general male population in Mumbai, India | **5** |
|  | Prevalence of soft-tissue lesions among women in sex work in the red light area of Pune, India: a cross-sectional survey | **3** |
|  | Prevalence of tobacco and areca-nut use among patients attending dental teaching hospital in the central province of Sri Lanka and its association with oral mucosal lesions; a cross sectional study | **3** |
|  | Prevalence of tobacco associated oral mucosal lesions in a private hospital-a retrospective study | **3** |
|  | Prevalence of tobacco associated oral mucosal lesions in the population of Mahbubnagar district of Telangana state: a cross-sectional study | **2** |
|  | Prevalence of tobacco habit and associated oral lesions | **3** |
|  | Prevalence of tobacco usage in oral leukoplakia: a retrospective study | **3** |
|  | Prevalence of tobacco use and oral malignant precancerous lesions among auto rickshaw drivers in Chennai city, Tamil Nadu, India | **5** |
|  | Prevalence of tobacco use and oral mucosal lesions among Nicobarese tribal population in Andaman and Nicobar islands | **3** |
|  | Prevalence of tobacco use and oral precancerous lesions, and their associated factors among adult females in rural areas of a primary health centre in Bangalore urban district | **3** |
|  | Prevalence of tobacco use, nicotine dependence and associated oral premalignant lesion in rural area of Nanded district of Maharashtra, India | **2** |
|  | Prevalence of tobacco use, potentially malignant lesions and oral cancer among irula tribes, Nilgiri Hills, Tamil Nadu, India | **5** |
|  | Prevalence of tobacco-associated oral mucosal lesion in Hazaribagh population: a cross-sectional study | **3** |
|  | Prevalence of type of chewing tobacco associated with osmf in patients reported in a private dental institution: a retrospective study | **6** |
|  | Prevalência da queilite actínica em agricultores de uma região do sertão brasileiro | **3** |
|  | Prevalência das lesões cancerizáveis na cavidade oral no município de campina grande - Paraíba - Brasil | **6** |
|  | Prevalencia de lesiones bucales en tejido blando encontradas en la clínica de estomatología de la facultad de odontología de la universidad de los andes: periodo 2015-2018 | **3** |
|  | Prevalencia de lesiones de la mucosa oral en pacientes que acuden a una clínica universitaria. Estudio transversal retrospectivo a 5 años | **6** |
|  | Prevalencia de lesiones en mucosa bucal y su relación con condición sistémica, edad y sexo | **4** |
|  | Prevalência de lesões da mucosa oral numa população idosa da região norte de portugal | **5** |
|  | Prevalência e caracterização de lesões orais brancas na população idosa institucionalizada | **3** |
|  | Prevention of upper aerodigestive tract cancer through active search strategies and use of equipped propaedeutics | **8** |
|  | Profile of patients with white lesions of the oral mucosa treated at a dental school in southern brazil | **3** |
|  | Queilitis actinica en pescadores artesanales de la quinta region: prevalencia y aspectos histopatologicos associados. | **3** |
|  | Relación entre los comportamientos de riesgo para la salud, los desórdenes orales potencialmente malignos y el estado de salud oral | **5** |
|  | Repercussions of smokeless tobacco on buccal mucosa: a community based observational study at a tertiary care centre in western Rajasthan | **3** |
|  | Retrospective study of oral lichen planus and oral lichenoid lesions: clinical profile and malignant transformation | **3** |
|  | Risk assessment of smokeless tobacco among oral precancer and cancer patients in eastern developmental region of Nepal | **7** |
|  | Role of tobacco consumption habits in the causation of precancerous lesions: a cross sectional study | **3** |
|  | Screening for oral cancer utilising risk-factor analysis is ineffective in high-risk populations | **3** |
|  | Screening of oral cancer pathology and risk factors at primary care level in jodhpur, india | **5** |
|  | Site specific prevalence of oral cancer and potentially malignant disorders among Japanese cigarette smokers | **8** |
|  | Site-specific incidence of benign and precancerous leukoplakias and cancers of the oral cavity | **6** |
|  | Smokeless tobacco use and oral neoplasia among urban Indian women | **2** |
|  | Social and behavioural associated factors of actinic cheilitis in rural workers | **8** |
|  | Spectrum of lip lesions in a tertiary care hospital: an epidemiological study of 3009 indian patients | **3** |
|  | Stomatological disorders in older people: an epidemiological study in the Brazil southern | **8** |
|  | Study of assessment of prevalence and patterns of tobacco use and tobacco induced oral lesions in rural population of western Maharashtra: an epidemiological study | **3** |
|  | Study of association of premalignant lesion of oral cavity with the use of areca nut in the state of assam, India | **5** |
|  | Study on clinicopathological spectrum of oral lesions at our tertiary care hospital | **7** |
|  | The activity report of the oral cancer screening in Tokyo dental college: -mass screening of the oral cancer screening in all area and individual screening in Chiba-city | **7** |
|  | The characteristics of patients with oral lichen planus and malignant transformation-a retrospective study of 271 patients | **4** |
|  | The clinical prevalence of actinic cheilitis among community health agents from the regional v subprefecture of Fortaleza | **3** |
|  | The detection of oral cancer and potentially malignant disorders in Ireland: an observational study of 100 cases | **4** |
|  | The effects of tobacco use in oral cavity and periodontal health in patients of Burdwan-an observational study | **3** |
|  | The impact of a pandemic on a military oral and maxillofacial pathology biopsy service | **1** |
|  | The predominance of tobacco propensities and tobacco-related oral lesions in textile mill workers of Bhopal: a cross-sectional study | **3** |
|  | The relationship of tobacco, alcohol, and betel quid with the formation of oral potentially malignant disorders: a community-based study from northeastern Thailand | **3** |
|  | The role of immunohistochemistry for primary oral diagnosis in a Brazilian oral pathology service | **5** |
|  | To assess the awareness of pre-malignant conditions in diagnosed cancer patients in private university hospital | **8** |
|  | To evaluate the level of awareness of patients about existing oral precancerous lesions: a longitudinal study | **4** |
|  | To the question of the prevalence of oral mucosal lesions in central Kazakhstan | **5** |
|  | Tobacco - the silent slayer for oral premalignant lesions/conditions among beedi rolling workers of Durg city, Chhattisgarh, India: a cross-sectional study | **3** |
|  | Tobacco abuse and associated oral lesions among interstate migrant construction workers | **3** |
|  | Tobacco addiction and it's consequences on oral health of indigenous and rural people - a cross sectional study from central India | **5** |
|  | Tobacco and betel nut chewing behavior and its association with potentially malignant disorders in Chennai | **3** |
|  | Tobacco chewing habits and risk of precancerous oral lesions among Paniya tribes of Wayanad, India - a cross sectional study | **5** |
|  | Tobacco related oral lesions in south Indian industrial workers | **2** |
|  | Tobacco use and clinical leukoplakia lesions among south Indian tribes | **2** |
|  | Tobacco use and incidence of adverse oral health outcomes among us adults in the population assessment of tobacco and health study | **2** |
|  | Tobacco use and oral premalignant lesions among auto-rickshaw drivers in Belagavi, north Karnataka | **3** |
|  | Tobacco use and prevalence of oral premalignant lesions, among Malayali tribes, Yelagiri hills, Tamil Nadu, India | **5** |
|  | Tobacco use, awareness and oral health among Kanchipuram silk weavers, Tamil Nadu, India | **5** |
|  | Tobacco use, body mass index, potentially malignant disorder and attitude towards passive smoking in a primary health centre – a cross‑sectional study | **2** |
|  | Tongue lesions and anomalies in a sample of Yemeni dental patients: a cross-sectional study | **3** |
|  | Type of tobacco used and its associated risk for premalignant lesions in young adolescents visiting a private dental hospital in Chennai | **2** |
|  | Uma análise retrospectiva de lesões malignas e desordens potencialmente malignas em sete anos | **3** |
|  | White oral mucosal lesions among the Yemeni population and their relation to local oral habits | **3** |
|  | Workplace based potentially malignant oral lesions screening among tobacco consuming migrant construction site workers in Chennai, south India: a pilot study | **2** |

**^Legend:^** ^Exclusion criteria applied:^ **^(1)^** ^OPMD data could not be extracted due to grouping with other conditions;^ **^(2)^** ^OPMDs were only linked to specific etiological factors (e.g., HPV, betel quid, tobacco);^ **^(3)^** ^diagnosis of OPMD was not confirmed by histopathological analysis;^ **^(4)^** ^prevalence of OPMD was not clearly reported or could not be calculated;^ **^(5)^** ^reviews, case reports, protocols, short communications, personal opinions, letters, posters, conference abstracts, thesis, dissertations, and laboratory research;^ **^(6)^** ^full texts were not available;^ **^(7)^** ^only oral lichen planus or other lichenoid lesions were included in the analysis, and^ **^(8)^** ^published in languages other than the Latin (Roman) alphabet.^

**References:**

1. Afridi NM, Khan RTU, Khan Z, et al. A clinical study of incidence and risk factors associated with oral premalignant lesions. Medical Forum Monthly. 2017;28:3-6.
2. Memon AB, Rahman AAU, Channar KA, et al. A Clinico-Demographic Evaluation of Patients with Oral Submucous Fibrosis: a Cross Sectional Study. J Pharmaceutic Research International. 2021;33:22-29. https://doi.org/10.9734/JPRI/2021/v33i1331262
3. Mishra S, Ifterkhar S. A Longitudinal Study on Oral Potentially Malignant and Malignant Lesions in a Tertiary Care Teaching Hospital. Indian Journal of Public Health Research and Development. 2023;14:347-353. https://doi.org/10.37506/ijphrd.v14i3.19428
4. Birur NP, Patrick S, Bajaj S, et al. A Novel Mobile Health Approach to Early Diagnosis of Oral Cancer. The journal of contemporary dental practice. 2018;19:1122-1128. https://doi.org/10.5005/jp-journals-10024-2392
5. Khanna D, Mishra A, Birur P, et al. A prospective study on diagnostic accuracy of technology-enabled early detection of oral cancer and epidemiology of tobacco and other substances use in rural India. Cancer. 2025;131: e35702. https://doi.org/10.1002/cncr.35702
6. Madankumar PD, Iyer K, Soni S, et al. A simple screening program for oral cancer in a defined geographic area in southern India: A community-based cross-sectional study. Cancer Research, Statistics, and Treatment. 2022;5:226-231. https://doi.org/10.4103/crst.crst_92_22
7. Jha R, V Parmar D, Kailasam S. A Study of Precancerous Lesions for Oral Cancer in Jamnagar City. JIAOMR. 2011;23: 333-335. https://doi.org/10.5005/jp-journals-10011-1162
8. Chiewwit P, Khovidhunkit SP, Tantipoj C, et al. A study of risk factors associated with the presence of oral potentially malignant disorders: a community-based study from Northeastern Thailand. BMC Oral Health. 2024;24:932. https://doi.org/10.1186/s12903-024-04554-6
9. Daftary DK, Pitkar VK, Gupta PC, et al. A study of the natural history of oral preleukoplakia. Acta Odontologica Scandinavica. 1978;36:327-331. https://doi.org/10.3109/00016357809029083
10. Ramasamy J, Sivapathasundharam B. A study on oral mucosal changes among tobacco users. J Oral Maxillofac Pathol. 2021;25:470-477. https://doi.org/10.4103/jomfp.jomfp_105_21
11. Faria MHD, Silva LMAC, Mafra RP, et al. Actinic cheilitis in rural workers: prevalence and associated factors. Einstein (Sao Paulo, Brazil). 2022;20:eAO6862. https://doi.org/10.31744/einstein_journal/2022AO6862
12. Rodríguez-Blanco I, Flórez A, Paredes-Suárez C, et al. Actinic Cheilitis Prevalence and Risk Factors: A Cross-sectional, Multicentre Study in a Population Aged 45 Years and Over in North-west Spain. Acta Dermato-Venereologica. 2018;98:970-974. https://doi.org/10.2340/00015555-3014
13. Maksimovskaya LN, Abramova MY, Erk AA. Análisis de los Resultados nacionales obtenidos por grupos estatales de investigación del el cáncer ruso. Programa de Cribado de Enfermedades de la Mucosa Oral y Borde Bermellón. Prensa méd argent. 2020;106:32-37.
14. Ishii S, Sakaguchi W, Sugai M, et al. Analysis of false-negatives in exfoliative cytology in oral potentially malignant disorders: A retrospective cohort study. J Stomatol Oral Maxillofac Surg 2022;123: 390-395. https://doi.org/10.1016/j.jormas.2022.02.001
15. Maksimovskaya LN, Abramova MY, Erk AA. Analysis of the results obtained by national research groups with the Russian state cancer program for the screening of diseases of the oral mucosa and the vermillion border. J Oral Maxillofac Pathol. 2020;24:582. https://doi.org/10.4103/jomfp.JOMFP_289_20
16. Taruna T, Singh YP, Waghmare R, et al. Analyzing the Frequency of Premalignant Lesions and Oral Malignancy in Indian Subjects Attending Outpatient Department From the Low Socioeconomic Group. Cureus. 2023;15:e42035. https://doi.org/10.7759/cureus.42035
17. Palliyal S. Assessment of betel quid habits and risk of precancerous oral lesions among paniya tribes of wayanad, india-a cross-sectional study. Journal of Global Oncology. 2018;4:12s. https://doi.org/10.1200/jgo.18.16700
18. Akshayak, Senthil Murugan P. Assessment of incidence of oral submucous fibrosis patients - an institutional study. International Journal of Dentistry and Oral Science. 2021;8:1939-1945.
19. Nithya VR, Krithika C, Sridhar C, et al. Assessment of Oral Health Care Needs among Fishermen Living in North Chennai, India - A Cross Sectional Study. Journal Of Pharmaceutical Research International. 2021;33:379-385. https://doi.org/10.9734/JPRI/2021/v33i58B34214
20. Choudhury AR, Ankola AV, Roopali S, et al. Assessment of oral health status and tobacco-related habits among the employees of North-West Karnataka Road Transport Corporation (NWKRTC), Belagavi City, India - A Cross-Sectional Study. International Journal of Occupational Safety and Health. 2022;12:299-306. https://doi.org/10.3126/ijosh.v12i4.43885
21. Rohini C, Vijayakumar N. Assessment of Oral Health Status and Treatment Needs Among Sugali Tribe in Telangana Region - A Cross-Sectional Study. Rajiv Gandhi University of Health Sciences (India); 2017. https://www.proquest.com/dissertations-theses/assessment-oral-health-status-treatment-needs/docview/2866357135/se-2?accountid=26642
22. Malar I, Jayakumar HL. Assessment of Oral Health Status and Treatment Needs of Tribal Population in Yelagiri Hlls, Vellore District, Tamil Nadu - A Cross-Sectional Study. Rajiv Gandhi University of Health Sciences (India); 2019. https://www.proquest.com/dissertations-theses/assessment-oral-health-status-treatment-needs/docview/2866356141/se-2?accountid=26642
23. Palliyal S. Assessment of oral lesions and its association with smoking habits in paniya tribes of india. Journal of Global Oncology. 2018;4:25s. https://doi.org/10.1200/jgo.18.38800
24. Kumar A, Saini RS, Sharma V, et al. Assessment of Pattern of Oral Prosthetic Treatment and Prevalence of Oral Diseases in Edentulous Patients in North Indian Population: A Cross-sectional Study. Journal Of Pharmacy And Bioallied Sciences. 2021;13:187-189. https://doi.org/10.4103/jpbs.JPBS_648_20
25. Soundarajan S, Gajendran PL. Assessment of periodontal status in patients with oral lesions-a retrospective study in South Indian population. Indian Journal of Forensic Medicine and Toxicology. 2020;14:5395-5405. https://doi.org/10.37506/ijfmt.v14i4.12466
26. Kumar C, Priyankesh, Pandey V, et al. Assessment of Potentially Malignant Disorders among Men and Women in South Bihar Population. Journal Of Pharmacy And Bioallied Sciences. 2024;16:S456-S458. https://doi.org/10.4103/jpbs.jpbs_713_23
27. Sumit, Shekhar P, Azmi N, et al. Assessment of Various Pre-Malignant Oral Lesions and Associated Factors at NMCH Patna. International Journal of Current Pharmaceutical Review and Research. 2024;16:261-265.
28. Babu PS, Rajendran D, Mani G. Association between oral leukoplakia smoking and alcohol habits in patients. European Journal of Molecular and Clinical Medicine. 2020;7:1211-1220.
29. Cury PR, Araujo NS, Oliveira MDA, et al. Association between oral mucosal lesions and crack and cocaine addiction in men: a cross-sectional study. Environmental Science And Pollution Research. 2018;25:19801-19807. https://doi.org/10.1007/s11356-018-2120-1
30. Ashwinirani SR, Girish S. Association between oral potentially malignant disorders with age, gender, site and habits in Western part of Maharashtra population. JK Science. 2020;22:202-206.
31. Karthikeyan G, Hannah R, Ramamurthy J. Association of clinical variants of leukoplakia with different types of tobacco - an institution based retrospective study. European Journal of Molecular and Clinical Medicine. 2020;7:1440-1448.
32. Lee CH, Ko AMS, Yang FM, et al. Association of DSM-5 betel-quid use disorder with oral potentially malignant disorder in 6 betel-quid endemic asian populations. JAMA Psychiatry. 2018;75:261-269. https://doi.org/10.1001/jamapsychiatry.2017.4307
33. Jani CT, Joshi A, Jain K, et al. Association of oral premalignant lesions with the consumption of tobacco. JNCCN Journal of the National Comprehensive Cancer Network. 2019;17:3625-3629. https://doi.org/10.6004/jnccn.2018.7261
34. Palliyal S. Betel nut chewing habits and risk of precancerous oral lesions among Paniya tribes of Wayanad, India: A cross sectional study. Annals of Oncology. 2017;28: 105-106. https://doi.org/10.1093/annonc/mdx665.020
35. Zhang S, Li W, Gao Y, et al. Betel‐quid and oral submucous fibrosis: a cross‐sectional study in Hunan province, China. J Oral Pathology Medicine. 2012;41:748-754. https://doi.org/10.1111/j.1600-0714.2012.01166.x
36. André CSGA, Freitas FMF, Caramês JMM. Biópsias Orais Numa População Portuguesa: Estudo Clinicopatológico dos Últimos 20 Anos numa Clínica Universitária. Universidade de Lisboa (Portugal); 2021. https://www.proquest.com/dissertations-theses/biópsias-orais-numa-população-portuguesa-estudo/docview/2658177552/se-2?accountid=26642 http://link.periodicos.capes.gov.br/sfxlcl41?url_ver=Z39.88-2004&rft_val_fmt=info:ofi/fmt:kev:mtx:dissertation&genre=dissertations&sid=ProQ:ProQuest+Dissertations+%26+Theses+Global&atitle=&title=Bi%C3%B3psias+Orais+Numa+Popula%C3%A7%C3%A3o+Portuguesa%3A+Estudo+Clinicopatol%C3%B3gico+dos+%C3%9Altimos+20+Anos+numa+Cl%C3%ADnica+Universit%C3%A1ria&issn=&date=2021-01-01&volume=&issue=&spage=&au=de+Almeida+Andr%C3%A9%2C+Cl%C3%A1udia+Sofia+Garcia&isbn=979-8-209-83679-7&jtitle=&btitle=&rft_id=info:eric/&rft_id=info:doi/
37. Muthanandam S, Babu BV, Muthu J, et al. Burden of oral precancer and cancer among an indigenous tribal population of South India - An evaluative study. Indian journal of dental research : official publication of Indian Society for Dental Research. 2022;33:253-257. https://doi.org/10.4103/ijdr.ijdr_552_21
38. Oyapero A. Burden of tobacco and alcohol consumption and its association with periodontal disease, potentially malignant lesions and quality of life among bus drivers in Lagos State, Nigeria. Tobacco Induced Diseases. 2019;17. https://doi.org/10.18332/tid/112091
39. Fonseca MSSAR, Freitas FMF, Caramês JMM. Caracterização Clínica e Histológica de uma População Diagnosticada com Líquen Plano Oral numa Clínica Universitária Portuguesa nos Últimos 22 Anos. Universidade de Lisboa (Portugal); 2020. https://www.proquest.com/dissertations-theses/caracterização-clínica-e-histológica-de-uma/docview/2637957971/se-2?accountid=26642
40. Trimboli C de F, Marques TMS, da Silva RMM, Couto PSS. Caracterização das Lesões de Tecidos Moles e Duros nos Pacientes da Clínica Dentária Universitária : Um Estudo Retrospetivo. Universidade Catolica Portuguesa (Portugal); 2022. https://www.proquest.com/dissertations-theses/caracterização-das-lesões-de-tecidos-moles-e/docview/2956851853/se-2?accountid=26642
41. Cabarcos YT, Sori BS, López AM, et al. Caracterización clínico e histopatológica de la leucoplasia bucal. Arch méd Camaguey. 2018;22:432-451.
42. Yang SF, Wang YH, Su NY, et al. Changes in prevalence of precancerous oral submucous fibrosis from 1996 to 2013 in Taiwan: A nationwide population-based retrospective study. Journal Of The Formosan Medical Association. 2018;117:147-152. https://doi.org/10.1016/j.jfma.2017.01.012
43. Rock LD, Rosin MP, Zhang L, et al. Characterization of epithelial oral dysplasia in non-smokers: First steps towards precision medicine. Oral Oncology. 2018;78:119-125. https://doi.org/10.1016/j.oraloncology.2018.01.028
44. Martínez C, Olivares P, Martínez B, et al. Characterization of patients with epithelial dysplasia and carcinoma in situ in the oral cavity, 2000 to 2014. Journal of Oral Research. 2020;9:109-113. https://doi.org/10.17126/joralres.2020.015
45. Mehta FS, Gupta PC, Pindborg JJ. Chewing and smoking habits in relation to precancer and oral cancer. J Cancer Res Clin Oncol. 1981;99:35-39. https://doi.org/10.1007/BF00412440
46. Wang H, He F, Xu C, et al. Clinical analysis for oral mucosal disease in 21 972 cases. Zhong nan da xue xue bao Yi xue ban = Journal of Central South University Medical sciences. 2018;43:779-783. https://doi.org/10.11817/j.issn.1672-7347.2018.07.013
47. Lorini L, Magri C, Gurizzan C, et al. Clinical and histological prognostic factors of malignant transformation in a large series of oral potentially malignant disorders (OPMDs). Tumori. 2021;107:138. https://doi.org/10.1177/03008916211041664
48. Lorini L, Tomasoni M, Gurizzan C, et al. Clinical and Histological Prognostic Factors of Recurrence and Malignant Transformation in a Large Series of Oral Potentially Malignant Disorders. Frontiers in oncology. 2022;12. https://doi.org/10.3389/fonc.2022.886404
49. Nivethitha R, Santhanam A, Dinesh SPS. Clinical characteristics of oral lichen planus in patients visiting a private dental institution-a retrospective study. International Journal of Pharmaceutical Research. 2020;13:1466-1473. https://doi.org/10.31838/ijpr/2021.13.01.220
50. Castro ZB, Aguilar VG, Barceló MLCG, et al. Clinical epidemiological evaluation of the oral premalignant lesions in the population of the Montalvo in Ambato, Ecuador. Revista Cubana de Estomatologia. 2019;56: e1561.
51. Abbas Y, Kanotra S, Majeed F, et al. Clinical Profile and Prevalence of Oral Mucosal Lesions in Tobacco Users-A Prospective Study from Jammu, India. Indian J Otolaryngol Head Neck Surg. 2024;76:2373-2380. https://doi.org/10.1007/s12070-023-04433-6
52. Debta FM, Debta P, Mishra E, et al. Clinico-epidemiological study of oral potentially malignant and malignant lesions in a tertiary care centre of odisha- A five year longitudinal study. Indian Journal of Public Health Research and Development. 2018;9:2127-2137. https://doi.org/10.5958/0976-5506.2018.01765.5
53. Kokubun K, Nakajima K, Akashi Y, et al. Clinicopathological evaluation of oral leukoplakia: a single-center study of 676 cases in Japan. Oral Surgery Oral Medicine Oral Pathology Oral Radiology. 2024;137:529-536. https://doi.org/10.1016/j.oooo.2024.02.022
54. Desai KM, Kale AD, Angadi PV, et al. Clinicopathological Evaluation Of Oral Submucous Fibrosis - A Retrospective, Single Institute Study. Annals Of Dental Specialty. 2021;9:27-33.
55. Gabhane MH, Hemagiriyappa MS, Sharma VJ, et al. Clinicopathological Evaluation of Tobacco-related Oral Mucosal Lesions. Journal of Contemporary Dental Practice. 2022;23:399-404. https://doi.org/10.5005/jp-journals-10024-3274
56. Raghav DD, Shobana G, Senthilnathan S, et al. Community-based early detection of oral precancerous lesion, accessibility and utilization of oral healthcare services among Irular tribes residing at union territory of Pondicherry. Journal of Family Medicine & Primary Care. 2024;13:1511-1516. https://doi.org/10.4103/jfmpc.jfmpc_1334_23
57. Oswal K, Chhabra M, Sarvepalli BK, et al. Community-based oral cancer screening for women of remote & rural blocks of the Chikkaballapur district, Karnataka, India. Clinical Epidemiology and Global Health. 2023;21. https://doi.org/10.1016/j.cegh.2023.101284
58. Shrestha G, Gautam DK, Siwakoti B, et al. Community-based Screening of Oral Cancer in Selected Districts of Nepal: A Cross-Sectional Study. Asian Pacific Journal of Cancer Prevention. 2023;24:4111-4115. https://doi.org/10.31557/APJCP.2023.24.12.4111
59. Shivashankar K, Divvi A, Kengadaran S, et al. Comparing the use of tobacco among fishermen and non-fishermen population of Pondicherry, India. Tobacco Induced Diseases, suppl APACT 2021. 2021;19. https://doi.org/10.18332/tid/140907
60. Akash N, Arivarasu L. Comparison of Prevalence of Leukoplakia IN Males and Females among Chennai Population. Journal Of Research In Medical And Dental Science. 2021;9:87-91.
61. Chiang TE, Lin YC, Wu CT, et al. Comparison of the accuracy of diagnoses of oral potentially malignant disorders with dysplasia by a general dental clinician and a specialist using the Taiwanese Nationwide Oral Mucosal Screening Program. Plos One. 2021;16: e0244740. https://doi.org/10.1371/journal.pone.0244740
62. Emamverdizadeh P, Arta SA, Ghanizadeh M, et al. Compatibility of Clinical and Histopathological Diagnosis of Oral Lesions in Iranian Patients. Pesqui bras odontopediatria clín integr. 2019;19:4344-4344. https://doi.org/10.4034/PBOCI.2019.191.01
63. Pupo OLZ, Infante ML, Santiesteban YC, et al. Comportamiento del Programa de Detección Precoz del Cáncer Bucal en la Clínica Artemio Mastrapa. Holguín, 2015. CCH, Correo cient Holguín. 2017;21:786-797.
64. Santana R dos S, Vita W dos S. Condição de saúde bucal de pacientes internados em um hospital público brasileiro. Rev Baiana Saúde Pública. 2024;48:169-184. https://doi.org/10.22278/2318-2660.2024.v48.n1.a4072
65. Navas-Aparicio M del C. Congruencia Diagnóstica Clínica-Histopatológica de Lesiones en Cavidad Oral, en la Faculta de Odontología, Universidad de Costa Rica, en el Período 2016-2019. Int j odontostomatol (Print). 2024;18:41-50.
66. Torabi M, Afshar MK, Afshar HM, et al. Correlation Between Clinical and Histopathologic Diagnosis of Oral Potentially Malignant Disorder and Oral Squamous Cell Carcinoma. Pesquisa Brasileira Em Odontopediatria E Clinica Integrada. 2021;21:e0143. https://doi.org/10.1590/pboci.2021.068
67. Rani K, Kishore B. Correlation of Oral Mucosal Lesions with Various Habits and Histopathological Findings. International Journal of Pharmaceutical and Clinical Research. 2024;16:797-801.
68. Morikawa T, Shibahara T, Takano M, et al. Countermeasure and opportunistic screening systems for oral cancer. Oral Oncology. 2021;112: 105047. https://doi.org/10.1016/j.oraloncology.2020.105047
69. Parmar JKS, Babariya MJ, Doshi C, et al. Cytopathological Study of Oral Lesions in Tertiary Care Hospital in Western Part of India. International Journal of Toxicological and Pharmacological Research. 2023;13:77-81.
70. Ravi P, Gopal SK, Lankupalli AS. Demographic analysis of prevalence of oral lesions in patients with deleterious habits– an institutional based descriptive observational study. International Journal of Pharmaceutical Research. 2021;13:1709-1715. https://doi.org/10.31838/ijpr/2021.13.03.211
71. Haitami S, El Mokhlis K, Hamza M, Ben Yahya I. Detection of potentially malignant lesions and squamous cell carcinomas in consultation of surgical denstitry. Tunis Med. 2016;94:29-33.
72. Shetty P, Khargekar NC, Debnath A, et al. Determinants of tobacco use and prevalence of oral precancerous lesions in cab drivers in Bengaluru city, India. International Journal of Preventive Medicine. 2017;8:100. https://doi.org/10.4103/ijpvm.IJPVM_225_17
73. Pandya D, Banerjee A, Dutta K, et al. Determination of the Prevalence of Oral Potentially Malignant Disorders among Tobacco users in a Subset of Kolkata Population. Journal of Clinical and Diagnostic Research. 2024;18:18.
74. Yadav NR, Jain M, Sharma A, et al. Distribution and prevalence of oral mucosal lesions in residents of old age homes in Delhi, India. Nepal Journal of Epidemiology. 2018;8:727-734. https://doi.org/10.3126/nje.v8i2.18708
75. Francis MS, Sundar M, Khot PB. Early detection of oral cancer and prevalence of tobacco use among the general population in Hassan District. Indian Journal of Cancer. 2021;58:53-54.
76. Hussain T, Riaz H, Bajwa SJ, et al. Effect of Smokeless Tobacco (ST) and areca nut chewing among adults in Gulyana Village Pakistan. Pakistan Journal of Medical and Health Sciences. 2018;12:946-949.
77. Chaudhuri S, Dey S, Awasthi A. Epidemiological hazards of tobacco and its manifestations in oral health of a screened population in Northern India. Journal of Cancer Policy. 2017;12:1-6. https://doi.org/10.1016/j.jcpo.2016.12.006
78. Kumar GK, Abidullah M, Elbadawi L, et al. Epidemiological profile and clinical characteristics of oral potentially malignant disorders and oral squamous cell carcinoma: A pilot study in Bidar and Gulbarga Districts, Karnataka, India. J Oral Maxillofac Pathol. 2019;23:90-96. https://doi.org/10.4103/jomfp.JOMFP_116_18
79. Saravanan S, Lenin KR. Epidemiological profile of tobacco users attending a tobacco cessation centre at Puducherry, India. Journal Of Indian Association Of Public Health Dentistry. 2022;20:69-74. https://doi.org/10.4103/jiaphd.jiaphd_116_21
80. Kolenko YG, Timokhina TO, Lynovytska OV, et al. Epidemiological Situation Of Pre-Cancer Diseases Of The Oral Mucous In Ukraine. Wiadomosci lekarskie. 2022;75:1453-1458. https://doi.org/10.36740/WLek202206105
81. Yatsenko AK, Trankovskaya LV, Pervov YY, et al. Epidemiological studies of the prevalence of the oral mucosa and the red lip border lesions. Kazan Medical Journal. 2023;104:99-107. https://doi.org/10.17816/KMJ71956
82. Almalki SA, Gowdar IM, Vengal M, et al. Epidemiology of quid usage and its possible association with the occurrence of oral mucosal lesions. Frontiers in Oral Health. 2024;5:1450729. https://doi.org/10.3389/froh.2024.1450729
83. Chiu SF, Ho CH, Chen YC, et al. Epidemiology profile and outcomes of oral potentially malignant disorders among different geographic regions of Taiwan: A retrospective observational nationwide population database study. Journal of Medical Sciences (Taiwan). 2021;41:286-294. https://doi.org/10.4103/jmedsci.jmedsci_257_20
84. Ramos ACS, Marques JS. Estudo retrospetivo da concordância entre o diagnóstico clínico e anatomopatológico em pacientes da clínica universitária Egas Moniz. Egas Moniz School of Health & Science (Portugal); 2019. https://www.proquest.com/dissertations-theses/estudo-retrospetivo-da-concordância-entre-o/docview/2906742294/se-2?accountid=26642
85. Castro ZB, Aguilar VG, Barceló M de la CG, et al. Evaluación clínico-epidemiológica de trastornos bucales potencialmente malignos en adultos de Montalvo en Ambato, Ecuador. Rev cuba estomatol. 2019;56:e2121-e2121.
86. Upadhyay V, Choubey S, Wadbude H. Evaluation of demographic profile, risk factors and clinical presentation of oral submucous fibrosis in patients attending ENT OPD. International Journal of Life Sciences Biotechnology and Pharma Research. 2024;13:232-236. https://doi.org/10.69605/ijlbpr_13.8.2024.39
87. Roman-Torres CVG, Neto EG, Pimentel AC, et al. Evaluation of lip diseases in peddlers on two beaches of Guarujá/Brazil: An observational study. Braz dent j. 2024;35:e24-6044. https://doi.org/10.1590/0103-6440202406044
88. Zoman KA, Alshunaifi K, Al-Mutairi M, et al. Evaluation of oral lesions and dental health in HIV-positive Saudi patients. Saudi Dental Journal. 2024;36:1601-1605. https://doi.org/10.1016/j.sdentj.2024.11.012
89. Shoorgashti R, Moshiri A, Lesan S. Evaluation of Oral Mucosal Lesions in Iranian Smokers and Non-smokers. Niger J Clin Pract. 2024;27:467-474. https://doi.org/10.4103/njcp.njcp_702_23
90. Maleki L, Kargahi N, Hatefi SE. Evaluation of oral pathologic lesions in elderly patients in Isfahan, Iran, 1989-2018 years. Braz dent sci. 2021;24:1-5. https://doi.org/10.14295/bds.2021.v24i1.2035
91. Priya MK, Srinivas P, Devaki T. Evaluation of the prevalence of oral mucosal lesions in a population of eastern coast of South India. Journal of International Society of Preventive & Community Dentistry. 2018;8:396-401. https://doi.org/10.4103/jispcd.JISPCD_207_17
92. Suárez-Fernández C, García-Pola M. Exploring the prevalence and risk factors of benign and premalignant oral lesions in an adult population from Northern Spain: a pilot study. Quintessence international. 2024;55:412-419. https://doi.org/10.3290/j.qi.b5136857
93. García AJ, Quintero SF de la M, Martínez JO, et al. Factores asociados a lesiones bucales premalignas en pacientes mayores de 60 años de un consultorio médico. Medicentro (Villa Clara). 2022;26:44-61.
94. Badri P, Lai HL, Ganatra S, et al. Factors Associated with Oral Cancerous and Precancerous Lesions in an Underserved Community: A Cross-Sectional Study. International Journal Of Environmental Research And Public Health. 2022;19:1297. https://doi.org/10.3390/ijerph19031297
95. Bhutia TD, Lad N, Mehendale A. Factors influencing tobacco use and associated premalignant conditions among police personnel. Indian Journal of Cancer. 2021;58:S15.
96. Redman RS, Diehl SR, Jones-Richardson T, et al. Follow-up study of veterans with white and red oral mucosal lesions at Veterans Affairs Dental Clinics. Clinical And Experimental Dental Research. 2023;9:82-92. https://doi.org/10.1002/cre2.677
97. Donohue-Cornejo A, Torre-y-Morán AD la, Torre-Morán GD la, et al. Frecuencia de Lesiones y Condiciones Orales en Pacientes Mayores de 18 Años en una Clínica de Atención Primaria en México. Int j odontostomatol (Print). 2018;12:129-133. https://doi.org/10.4067/S0718-381X2018000100129
98. Yadav A, Guttal KS, Burde K. Frequency of oral mucosal lesions among tobacco chewers: A cross-sectional survey. Journal of Advanced Clinical and Research Insights. 2019;6:39-42. https://doi.org/10.15713/ins.jcri.256
99. Molania T, Nahvi A, Salehi M, et al. Frequency of oral mucosal lesions and awareness of these lesions in patients attending oral and maxillofacial clinic in Sari Dental School, Iran. Journal of Mazandaran University of Medical Sciences. 2017;26:80-87.
100. Osiak M, Szubińska-Lelonkiewicz D, Wychowański P, et al. Frequency of Pathologic Changes in the Oral Cavity in Patients Subjected to Long-term Pharmacologic Immunosuppressive Therapy After Kidney, Liver, and Hematopoietic Cell Transplantation. Transplantation Proceedings. 2018;50:2176-2178. https://doi.org/10.1016/j.transproceed.2018.04.003
101. Dalirsani Z, Ghazi A, Firouzabadi MG. Frequency of Systemic Diseases and Oral Lesions Among the Institutionalized Elderly Subjects in the Northeast of Iran. Indian Journal of Dermatology. 2023;68:723-728. https://doi.org/10.4103/ijd.ijd_246_23
102. Toro-Alzate M, Saldarriaga-Saldarriaga A, Sánchez-Muñoz LB, et al. Frequency, distribution, and risk factors of oral mucosal lesions in a Colombian population: cross-sectional study. Revista de la Facultad de Odontología Universidad de Antioquia. 2024;36:12-23. https://doi.org/10.17533/udea.rfo.v36n2a1
103. Lal KK, Lal D. From Plate to Palette: Dietary Patterns and Their Role in Mucosal Lesions Among North Indian Communities: A cross-sectional study. Rev Cient Odontol (Lima). 2024;12:e217. https://doi.org/10.21142/2523-2754-1204-2024-217
104. Amarasinghe AAHK, Usgodaarachchi US, Johnson NW, et al. High prevalence of lifestyle factors attributable for oral cancer, and of oral potentially malignant disorders in rural Sri Lanka. Asian Pacific Journal of Cancer Prevention. 2018;19:2485-2492. https://doi.org/10.22034/APJCP.2018.19.9.2485
105. Martins-de-Barros AV, Barros AMI, Silva CCG, et al. High prevalence of oral potentially malignant disorders and risk factors in a semi-urban brazilian city: a population-based cross-sectional study. Medicina Oral Patologia Oral Y Cirugia Bucal. 2021;26:E778-E785. https://doi.org/10.4317/medoral.24747
106. Madalli R, Reddy MGS, Bagul N, et al. Histopathological evaluation of white lesions– an institutional observational study. Indian Journal of Public Health Research and Development. 2018;9:70-74. https://doi.org/10.5958/0976-5506.2018.00615.0
107. Al-Wesabi MA, Al –Matari SM, Al-Jawfi KA. Histopathological Findings of Oral and Maxillofacial Biopsies from a Sample of Yemeni Patients. Journal of International Dental and Medical Research. 2021;14:235-241. https://doi.org/10.21203/rs.2.18387/v1
108. Shemawat S, Apurva S, Sharma A, et al. Histopathological Spectrum of Lesions of Oral Cavity and Oropharynx in A Tertiary Health Centre. International Journal of Toxicological and Pharmacological Research. 2023;13:28-34.
109. Gowthami MRS, Mahanthacary V. Histopathological Study of Lesions of Oral Cavity and Oropharynx in Rajarajeswari Medical College & Hospital. Rajiv Gandhi University of Health Sciences (India); 2018. https://www.proquest.com/dissertations-theses/histopathological-study-lesions-oral-cavity/docview/2866351292/se-2?accountid=26642
110. Yen AM, Wang S, Peng B, et al. Impact of oral potentially malignant disorder subtypes on all‐cause and cause‐specific mortality in males. Oral Diseases. 2019;25:750-757. https://doi.org/10.1111/odi.13028
111. Jadeja N, Manghnani P, Parikh P, et al. Impact Of Smoking And Smokeless Tobacco On Oral Cavity. Journal Of Pharmaceutical Negative Results. 2022;13:288-295. https://doi.org/10.47750/pnr.2022.13.S06.041
112. Caldeira PC, Schuch LF, Tavares TS, et al. Impact of the COVID-19 pandemic on public University laboratories of oral and maxillofacial pathology: A Brazilian multicenter study. Oral Diseases. 2022;28:2423-2431. https://doi.org/10.1111/odi.14136
113. Prachishree L, Panda J, Pattanayak D, et al. Impact of tobacco chewing on oral health: a hospital-based study in eastern, India. International Journal of Academic Medicine and Pharmacy. 2024;6:1788-1791. https://doi.org/10.47009/jamp.2024.6.1.353
114. Liu SY, Feng IJ, Wu YW, et al. Implication for second primary cancer from visible oral and oropharyngeal premalignant lesions in betel-nut chewing related oral cancer. Head And Neck-Journal For The Sciences And Specialties Of The Head And Neck. 2017;39:1428-1435. https://doi.org/10.1002/hed.24777
115. Škrinjar I. Incidence of oral mucosal diseases among croatian patients at university hospital centre zagreb. Acta Stomatologica Croatica. 2021;55:105.
116. Kumar JS, Narayan V, Arun M. Incidence of oral white lesions in patients attending a private dental hospital. International Journal of Research in Pharmaceutical Sciences. 2020;11:435-440. https://doi.org/10.26452/ijrps.v11iSPL3.2959
117. Kumaran H, Narayan V, Nivedhitha MS. Incidence of potentially malignant oral disorders in patients attending a private dental hospital. International Journal of Research in Pharmaceutical Sciences. 2020;11:1710-1714. https://doi.org/10.26452/ijrps.v11iSPL3.3499
118. Baskar K, Maragathavalli G, Dharman S. Incidence of tobacco related white lesions in patients. International Journal of Dentistry and Oral Science. 2020;7:878-880. https://doi.org/10.19070/2377-8075-20000174
119. Gupta PC, Mehta FS, Daftary DK, et al. Incidence rates of oral cancer and natural history of oral precancerous lesions in a 10‐year follow‐up study of Indian villagers. Comm Dent Oral Epid. 1980;8:287-333. https://doi.org/10.1111/j.1600-0528.1980.tb01302.x
120. Gatti PC, Galli EP, Montes de Oca H, et al. Incidencia de patologías bucomaxilares en un período de aislamiento social preventivo y obligatorio de la pandemia COVID-19. Rev Fac Odontol (BAires). 2021;36:63-69.
121. Rashid S, Manzar S, Kazmi F, et al. Influence of Risk Habits on Demographic Factors and its Impact on Oral Submucous Fibrosis. Pakistan Journal of Medical and Health Sciences. 2021;15:3058-3061. https://doi.org/10.53350/pjmhs2115113058
122. Mahapatra S, Chaly PE, Mohapatra SC, Madhumitha M. Influence of tobacco chewing on oral health: A hospital-based cross-sectional study in Odisha. Indian Journal of Public Health. 2018;62:282-286. https://doi.org/10.4103/ijph.IJPH_327_17
123. Chauhan N, Paul S, Bhadauria US, et al. Investigating the association between tobacco use and oral health among security guards at a tertiary healthcare centre in New Delhi: a cross-sectional study. Frontiers in Oral Health. Published online 2024;5:1375792. https://doi.org/10.3389/froh.2024.1375792
124. Dilsiz A, Sevinç Gü SNL. Investigation of Biopsied Non-Plaque-Induced Gingival Lesions in a Turkish Population: A 5-Year Retrospective Study. Eurasian J Med. 2023;55:100-103. https://doi.org/10.5152/eurasianjmed.2023.0088
125. Bhat M, Bhat S, Roberts-Thomson K, et al. Is periodontitis independently associated with potentially malignant disorders of the oral cavity? Asian Pacific Journal of Cancer Prevention. 2019;20:283-287. https://doi.org/10.31557/APJCP.2019.20.1.283
126. Yeung CA. Is the use of tobacco products, especially electronic nicotine delivery systems (ENDS), associated with the incidence of oral health outcomes among US adults? Evidence-based dentistry. 2023;24:161-162. https://doi.org/10.1038/s41432-023-00949-6
127. Hernández Cuétara L, Ramírez Ramírez R, Serrano Díaz B, Fernández Queija Y. Lesiones cancerígenas y precancerosas bucales. Factores de riesgos asociados. Policlínico Bernardo Posse. Año 2017. Rev medica electron. 2019;41:618-627.
128. Molina YG, Lara MG, Morales AC. Lesiones premalignas y malignas en el complejo bucal en La Palma, Pinar del Río. Rev cienc med Pinar Rio. 2018;22:61-69.
129. Aldagistani A, Marques T, Couto P. Lesões Leucoplásicas Nos Utentes da Clínica Dentária Universitária : Um Estudo Retrospetivo de 17 Anos. Universidade Catolica Portuguesa (Portugal); 2022. https://www.proquest.com/dissertations-theses/lesões-leucoplásicas-nos-utentes-da-clínica/docview/2956852833/se-2?accountid=26642
130. Porto UN, Laureano NK, Dos Santos NS, et al. Leukoplakia and erythroplakia in youngers versus older individuals: a clinicopathological retrospective study. Medicina Oral Patologia Oral y Cirugia Bucal. 2024;29:665-672. https://doi.org/10.4317/medoral.26659
131. Pierin EG, Sassi LM, Schussel JL. Malignant Transformation of Actinic Cheilitis: A Decade-long Retrospective Study in Southern Brazil. J Clin Exp Dent. 2024;16:e666-e669. https://doi.org/10.4317/jced.61590
132. Gurudath S, Desai R, Birur P, Patrick S. Mobile health approach for follow-up care in early oral cancer screening program. Head and Neck. 2017;39:E134-E135.
133. Razak IA, Ghani WMN, Doss JG, et al. Mouth self-examination (MSE) as a screening tool for oral potentially malignant disorders among a high-risk indigenous population with a low socioeconomic status. Journal of Global Oncology. 2018;4:48s. https://doi.org/10.1200/jgo.18.37700
134. Głowacka B, Konopka T. Needs for gerodontological treatment in the elderly living in lower silesia. Dental and Medical Problems. 2019;56:89-96. https://doi.org/10.17219/dmp/103094
135. Chaudhuri S, Gupta SK, Kumar N, et al. Nonsmoked tobacco addiction playing a major role in oral mucosal changes in northern indian population over smoked and alcohol use. Journal of Global Oncology. 2018;4:56s. https://doi.org/10.1200/jgo.18.79000
136. Axéll T. Occurrence of leukoplakia and some other oral white lesions among 20 333 adult Swedish people. Comm Dent Oral Epid. 1987;15:46-51. https://doi.org/10.1111/j.1600-0528.1987.tb00479.x
137. Meena JK, Verma A, Upadhyay SK. Opportunistic screening for detection and socio-epidemiological risk assessment of oral cancer patients in rural Jodhpur, India. Indian Journal of Medical and Paediatric Oncology. 2018;39:452-455. https://doi.org/10.4103/ijmpo.ijmpo_90_17
138. Madjova C, Chokanov S. Oral and dental status of bulgarian patients- a 5-year study. Journal of IMAB - Annual Proceeding (Scientific Papers). 2018;24:1891-1895. https://doi.org/10.5272/jimab.2018241.1891
139. Fonseca MF, Kato CO, Pereira MC, et al. Oral and maxillofacial lesions in older individuals and associated factors: A retrospective analysis of cases retrieved in two different services. J Clin Exp Dent. 2019;11:e921-e929. https://doi.org/10.4317/jced.56194
140. Reis CSM, Reis JGC, Conceição-Silva F, Valete CM. Oral and oropharyngeal mucosal lesions: clinical-epidemiological study of patients attended at a reference center for infectious diseases. Braz j otorhinolaryngol. 2024;90:101396-101396. https://doi.org/10.1590/S1808-86942024000300302
141. Pivovar A, Santos ZFDG, Torres‐Pereira CC. Oral cancer screening for high-risk individuals in the primary healthcare setting using an active approach. Journal of Oral Pathology & Medicine. 2017;46:786-791. https://doi.org/10.1111/jop.12552
142. Li H, Wen X, Li S, et al. Oral health conditions and unmet need among Chinese male adults: a national oral health survey. BMC Public Health. 2025;25:836. https://doi.org/10.1186/s12889-025-22070-2
143. Palliyal SA. Oral health disparities among privileged and underprivileged tribes of south India - A study on precancerous oral lesions prevalence. Annals of Oncology. 2019;30:ix104-ix105. https://doi.org/10.1093/annonc/mdz428.025
144. Francis DL. Oral Health Inequalities Among Tobacco Users in the Tea Plantation Workers, Nilgiri Hills, Tamilnadu, India. Cancer Epidemiology Biomarkers and Prevention. 2023;32:65. https://doi.org/10.1158/1538-7755.ASGCR23-Abstract-65
145. Koike BDV, Valões RMP, Cazal C, et al. Oral health of an indigenous population in northeastern Brazil: a cross-sectional Study of the Fulni-ô ethnic group. Sao Paulo Med J. 2024;142(1):e2022355. https://doi.org/10.1590/1516-3180.2022.0355.R1.10042023
146. Saintrain MV de L, Bandeira ABV, Pequeno LL, et al. Oral health of older people: tracking soft tissue injuries for the prevention of oral cancer. Rev Esc Enferm USP. 2018;52:e03380-e03380. https://doi.org/10.1590/s1980-220x2017033603380
147. Loka SR, Doshi D, Kulkarni S, Reddy LS, Baldava P, Dasari T. Oral health status among lorry drivers in Hyderabad city - A cross sectional study. Work. 2024;79:1627-1635. https://doi.org/10.3233/WOR-210849
148. Rahmath MP, Balasubramaniam A, Indiran MA, et al. Oral health status and behaviour among Kanikkaran tribes of Mundandhurai Hills in Tirunelveli district, Tamil Nadu. J Oral Biol Craniofac Res. 2024;14:245-251. https://doi.org/10.1016/j.jobcr.2024.03.006
149. Mahalakshmi K, Balasubramaniam A, Arumugham IM. Oral Health Status and Oral Health Risks Among Tribes in Tamil Nadu, India: An Epidemiological Study. Cureus. 2023;15:e48721. https://doi.org/10.7759/cureus.48721
150. Jippy RS, Ananda SR. Oral Health Status and Treatment Needs Among Adult Tribal Population in Attappadi, Kerala. Rajiv Gandhi University of Health Sciences (India); 2017. https://www.proquest.com/dissertations-theses/oral-health-status-treatment-needs-among-adult/docview/2866084968/se-2?accountid=26642
151. Halappa M. Oral health status and treatment needs among multiple factory workers, Tumkur City u A cross sectional study. Journal Of Indian Association Of Public Health Dentistry. 2020;18:232-235. https://doi.org/10.4103/jiaphd.jiaphd_10_20
152. Kumar S, Priyaranjan P, Basak D, et al. Oral health status and treatment needs of chromium mine workers in India. Indian Journal of Occupational and Environmental Medicine. 2022;26:172-177. https://doi.org/10.4103/ijoem.ijoem_223_21
153. Sachan R, Vijayakumar N. Oral Health Status and Treatment Needs of Santhal Tribe in Ponda Village,Bokaro District, Jharkhanda Cross-Sectional Study. Rajiv Gandhi University of Health Sciences (India); 2018. https://www.proquest.com/dissertations-theses/oral-health-status-treatment-needs-santhal-tribe/docview/2866081750/se-2?accountid=26642
154. Ogundana O, Uti O, Sofola O, et al. Oral Lesions Associated With Tobacco Smoking in Adolescents/Adults Dwellers in Lagos, Nigeria. Cancer Epidemiology Biomarkers and Prevention. 2023;32:66-66. https://doi.org/10.1158/1538-7755.ASGCR23-Abstract-66
155. Soares AC, Gomes APN, Calderipe CB, et al. Oral leukoplakia and erythroplakia in young patients: a southern Brazilian multicenter study. Braz oral res. 2024;38:e069-e069. https://doi.org/10.1590/1807-3107bor-2024.vol38.0069
156. Dogenski LC, Ribeiro SD, Gambin DJ, et al. Oral leukoplakia-epidemiological survey and histochemical analysis of 107 cases in Brazil. Clinical oral investigations. 2021;25:1859-1867. https://doi.org/10.1007/s00784-020-03488-x
157. Wilsch L, Hornstein OP, Brüning H, et al. Oral leukoplakia. II. Results of a year-long polyclinical pilot study. Dtsch Zahnarztl Z. 1978;33:132-142.
158. Pullishery F. Oral malignancies and tobacco related habits among Aranadar tribals in Kerala, India: a population based study. Tobacco Induced Diseases, suppl 17th World Conference on Tobacco or Health, WCTOH. 2018;16:A385. https://doi.org/10.18332/tid/84195
159. Tallada A, Ahmed J, Shenoy N, et al. Oral mucosal lesions and conditions in patients with dermatologic diseases: How common is it? Indian Journal of Public Health Research and Development. 2019;10:86-90. https://doi.org/10.5958/0976-5506.2019.02405.7
160. Bozdemir E, Yilmaz HH, Orhan H. Oral mucosal lesions and risk factors in elderly dental patients. Journal of Dental Research, Dental Clinics, Dental Prospects. 2019;13:24-30. https://doi.org/10.15171/joddd.2019.004
161. Bardellini E, Amadori F, Conti G, et al. Oral mucosal lesions in electronic cigarettes consumers versus former smokers. Acta Odontologica Scandinavica. 2018;76:226-228. https://doi.org/10.1080/00016357.2017.1406613
162. Karki A, Manandhar V, Maharjan R, et al. Oral Mucosal Lesions in Patients Attending Dermatology Outpatient Department of a Tertiary Care Center in Kathmandu: A Descriptive Cross-sectional Study. JNMA J Nepal Med Assoc. 2024;62:387-391. https://doi.org/10.31729/jnma.8618
163. Pereira D, Andrade M, Moreira A, et al. Oral pathology in a population observed within an oral cancer screening developed in Portugal. Med Oral Patol Oral Cir Bucal. 2025;30:e107-e116. https://doi.org/10.4317/medoral.26863
164. Gurung D, Joshi U, Chaudhary B, et al. Oral Potentially Malignant Disorders among Patients Attending the Department of Oral Medicine and Radiology of a Tertiary Care Dental Hospital: A Descriptive Cross-sectional Study. Journal Of Nepal Medical Association. 2022;60:453-456. https://doi.org/10.31729/jnma.7494
165. Acharya Mainali A, Acharya B. Oral potentially malignant disorders and oral cancer in nepalese dental patients. Supportive Care in Cancer. 2017;25:S157. https://doi.org/10.1007/s00520-017-3704-x
166. Aittiwarapoj A, Juengsomjit R, Kitkumthorn N, et al. Oral Potentially Malignant Disorders and Squamous Cell Carcinoma at the Tongue: Clinicopathological Analysis in a Thai Population. Eur J Dent. 2019;13:376-382. https://doi.org/10.1055/s-0039-1698368
167. Bassyoni L, Nicolau B, Makhoul N, et al. Oral Potentially Malignant Disorders Clinical Database at the Oral and Maxillofacial Surgery Department, Montreal General Hospital–McGill University Health Centre. McGill University (Canada); 2018. https://www.proquest.com/dissertations-theses/oral-potentially-malignant-disorders-clinical/docview/2501230291/se-2?accountid=26642
168. Pires FR, Barreto MEZ, Nunes JGR, et al. Oral potentially malignant disorders: clinical-pathological study of 684 cases diagnosed in a Brazilian population. Medicina Oral Patologia Oral Y Cirugia Bucal. 2020;25:E84-E88. https://doi.org/10.4317/medoral.23197
169. Sari EF, McCullough M, Cirillo N. Oral pre-malignant and malignant lesion detection among Indonesians: The prevalence and risk factors. Head and Neck. 2017;39:E58.
170. Gilligan G, Panico R, Lazos J, et al. Oral squamous cell carcinomas and oral potentially malignant disorders: A Latin American study. Oral Dis. 2024;30:2965-2984. https://doi.org/10.1111/odi.14778
171. Hazarey VK, Erlewad DM, Mundhe KA, et al. Oral submucous fibrosis: study of 1000 cases from central India. J Oral Pathology Medicine. 2007;36:12-17. https://doi.org/10.1111/j.1600-0714.2006.00485.x
172. de Freitas AISM, Louraço AC, Caramês JMM. Pacientes Geriátricos na Clínica de Medicina e Cirurgia Oral da Fmdul. Universidade de Lisboa (Portugal); 2019. https://www.proquest.com/dissertations-theses/pacientes-geriátricos-na-clínica-de-medicina-e/docview/2637955775/se-2?accountid=26642
173. Mehrotra D, Kumar S, Mishra S, et al. Pan masala habits and risk of oral precancer: A cross-sectional survey in 0.45 million people of North India. Journal of Oral Biology and Craniofacial Research. 2017;7:13-18. https://doi.org/10.1016/j.jobcr.2016.12.003
174. Indu M, Cherian LM, Menon PA, et al. Pattern of Distribution of Biopsy Confirmed Oral and Maxillofacial Lesions in Adult and Geriatric Age Groups of Central Kerala Population-An Institutional Retrospective Study of 11 Years. Oral & Maxillofacial Pathology Journal. 2023;14:159-164.
175. Badamali J, Das A, Satapathy KC, et al. Patterns and distribution of tobacco use and its association with oral precancerous lesions among the tribes of Odisha. Journal Of Substance Use. 2023. https://doi.org/10.1080/14659891.2023.2197052
176. Khan A, Ongole R, Baptist J, et al. Patterns of Tobacco Use and its Relation to Oral Precancers and Cancers among Individuals Visiting a Tertiary Hospital in South India. The journal of contemporary dental practice. 2020;21:304-309. https://doi.org/10.5005/jp-journals-10024-2791
177. Pombo SQ da R, Soares M de L, Novaes OGS, et al. Perfil dos Pacientes Atendidos no Curso de Odontologia do Sertão de Pernambuco: Perfil dos Pacientes Atendidos no Sertão. Rev cir traumatol buco-maxilo-fac. 2019;19:6-12.
178. Nunes C da S. Perfil epidemiológico de um Serviço de Medicina Bucal: estudo retrospectivo de 17 anos. Published online 2024. https://repositorio.unesp.br/server/api/core/bitstreams/5c160302-e9db-4b04-8206-3b2c4a5f2261/content
179. de Almeida IFB, Oliveira SS, Freitas KS, et al. Perfil epidemiológico e social de indivíduos com desordens orais potencialmente malignas. Interfaces Científicas-Saúde e Ambiente. 2024;9:864-878.
180. Martínez EL, Sánchez M del CR, Herrera IF, et al. Pesquisaje de lesiones premalignas y malignas en la cavidad bucal. Revista Cubana de Medicina General Integral. 1996;12:216-221.
181. Pérez YF, Aréchaga DP, García TB, et al. Potencial de transformación maligna de las lesiones blanquecinas bucales. Rev cuba med mil. 2021;50:e1071-e1071.
182. Thomson PJ, Goodson ML, Smith DR. Potentially malignant disorders revisited-The lichenoid lesion/proliferative verrucous leukoplakia conundrum. Journal of Oral Pathology & Medicine. 2018;47:557-565. https://doi.org/10.1111/jop.12716
183. Shete M, Kamble PP, Patil K, et al. Predictors of Dysplasia in Oral Submucous Fibrosis: A Retrospective Observational Study. Cureus. 2025;17:e78481. https://doi.org/10.7759/cureus.78481
184. Kumar A, Agrawal R, Misra SK, et al. Prevalence and biosocial determinants of Potentially Malignant Disorders of Oral Soft Tissue in slum population of Western Uttar Pradesh. Indian Journal of Community Health. 2017;29:376-381. https://doi.org/10.47203/IJCH.2017.v29i04.007
185. Jose C, Medappa BKA, Fareed N, et al. Prevalence and determinants of oral potentially malignant disorders in rural areas of South India. Journal of Cancer Research & Therapeutics. 2023;19:773-777. https://doi.org/10.4103/jcrt.jcrt_1151_21
186. Ramesh RM, Patrick S, Lotha Z, et al. Prevalence and determinants of oral potentially malignant lesions using mobile health in a rural block, northeast India. Trop Doct. 2022;52:53-60. https://doi.org/10.1177/00494755211049973
187. Alshayeb M, Mathew A, Varma S, et al. Prevalence and distribution of oral mucosal lesions associated with tobacco use in patients visiting a dental school in Ajman. Onkologia i Radioterapia. 2019;46(1):29-33.
188. Sami El T, Cassia A, Bouchi N, et al. Prevalence and Distribution of Oral Mucosal Lesions by Sex and Age Categories: A Retrospective Study of Patients Attending Lebanese School of Dentistry. International Journal of Dentistry. 2018;2018:4030134. https://doi.org/10.1155/2018/4030134
189. Sania, Rhythm, Mohanty S, et al. Prevalence and Distribution of Oral Mucosal Lesions in Jammu, Jammu & Kashmir: A Prospective Study. International Journal of Life Sciences Biotechnology and Pharma Research. 2024;13:658-663. https://doi.org/10.69605/ijlbpr_13.11.2024.116
190. Shah JS, Dubey J. Prevalence and factors associated with oral potentially malignant disorders and oral squamous cell carcinoma: An institutional study. Journal of Cancer Research and Therapeutics. 2023;19:S536-S444. https://doi.org/10.4103/jcrt.jcrt_759_22
191. Agarwal V, Maiti S. Prevalence and management of oral submucous fibrosis and its implications on prosthodontic treatment: A retrospective study. International Journal of Research in Pharmaceutical Sciences. 2020;11:1702-1709. https://doi.org/10.26452/ijrps.v11iSPL3.3498
192. Mikhalev DE, Baydik OD, Mukhamedov MR, et al. Prevalence and organization problems of medical care to patients with precanceral oral cavity mucosa diseases (on the Tomsk Region example). Head and Neck Tumors. 2022;12:79-85. https://doi.org/10.17650/2222-1468-2022-12-1-79-85
193. Das A, Doraikanan SS, Doraiswamy JN, et al. Prevalence and Pattern of Tobacco-associated Oral Lesion among Migrant Construction Workers in Chennai: A Cross-sectional Study. Journal of Pioneering Medical Sciences. 2024;13:151-156. https://doi.org/10.61091/jpms2024130723
194. Ankit K, Khan Y, Jaiswal A, et al. Prevalence and Patterns of Oral Mucosal Lesions among Geriatric Patients in India: A Retrospective Study. Journal of Pharmacy and Bioallied Sciences. 2024;16:S2303-S2305. https://doi.org/10.4103/jpbs.jpbs_211_24
195. Singh AK, Chauhan R, Anand K, Singh M, Das SR, Sinha AK. Prevalence and Risk Factors for Oral Potentially Malignant Disorders in Indian Population. Journal Of Pharmacy And Bioallied Sciences. 2021;13:398-401. https://doi.org/10.4103/jpbs.JPBS_751_20
196. Akhlaq H, Khan MSU, Nasir M, et al. Prevalence and Risk Factors of Oral Mucosal Lesions: A Retrospective Study of Patients Attending Oral Diagnosis Department of Siohs Karachi, Pakistan. Pakistan Journal of Medical and Health Sciences. 2021;15:3273-3278. https://doi.org/10.53350/pjmhs2115113273
197. Sari EF, Johnson NW, McCullough MJ, et al. Prevalence and risk factors of oral potentially malignant disorders in Indonesia: a cross-sectional study undertaken in 5 provinces. Scientific Reports. 2024;14:5232. https://doi.org/10.1038/s41598-024-54410-4
198. Kindler S, Samietz S, Dickel S, et al. Prevalence and risk factors of potentially malignant disorders of the mucosa in the general population Mucosa lesions a general health problem? Annals of anatomy. 2021;237 :151724. https://doi.org/10.1016/j.aanat.2021.151724
199. Kumar S, Narayanan VS, Ananda SR, et al. Prevalence and risk indicators of oral mucosal lesions in adult population visiting primary health centers and community health centers in Kodagu district. journal of family medicine and primary care. 2019;8:2337-2342. https://doi.org/10.4103/jfmpc.jfmpc_344_19
200. Hemashree J, Chaudhary M. Prevalence and treatment for cheilitis - A retrospective study. International Journal of Research in Pharmaceutical Sciences. 2020;11:428-432. https://doi.org/10.26452/ijrps.v11iSPL4.3877
201. Miranda MC, Batistella EÂ, Modolo F, et al. Prevalence of actinic cheilitis and lip squamous cell carcinoma among lip lesions. Oral Surgery, Oral Medicine, Oral Pathology & Oral Radiology. 2022;134:e199-e199. https://doi.org/10.1016/j.oooo.2022.01.610
202. Santos RF dos, Oliveira RL de, Gallottini M, et al. Prevalence of and factors associated with actinic cheilitis in extractive mining workers. Braz dent j. 2018;29:214-221. https://doi.org/10.1590/0103-6440201801605
203. Nethan ST, Kumar V, Sharma S, et al. Prevalence of gul use, its predictors and association with oral potentially malignant disorders and oral cancer development in the users of Noida, India: A cross-sectional study. Journal of Global Oncology. 2018;4:24s. https://doi.org/10.1200/jgo.18.31700
204. Bhattacharjee T, Debarshi J, Gangopadhyay S. Prevalence of habit-related oral lesions in Kolkata and the surrounding districts. Indian Journal of Multidisciplinary Dentistry. 2019;9:106-110. https://doi.org/10.4103/ijmd.ijmd_1_20
205. Shahzan MS, Chaudhary M, Marimuthu M. Prevalence of homogenous and non homogenous leukoplakia in a private dental hospital. International Journal of Dentistry and Oral Science. 2020;2:88-92.
206. Fernández-Chaves JM, Boza-Oreamuno YV, Masís-Monestel C, et al. Prevalence of Lesions and Oral Mucosal Normal Variations in an Elderly Population in Costa Rica. Odovtos. 2024;26:113-127. https://doi.org/10.15517/ijds.2023.57081
207. Vaish R, Jena D. Prevalence of leukoplakia in relation to tobacco habits in southern Orissa. Journal of the Indian Dental Association. 1982;54:419-424.
208. BintiZaaba NA, Chaudhary M, ShanthaSundari KK. Prevalence of leukoplakia, oral lichen planus and tobacco pouch keratosis -among patients visiting a private dental institution. International journal of early childhood special education. 2022;14:9569-9577. https://doi.org/10.9756/INT-JECSE/V14I3.1095
209. Haripriya R, Sherlin HJ, Nivethigaa B. Prevalence of lip lesions in patients visiting a dental hospital. International Journal of Research in Pharmaceutical Sciences. 2020;11:888-893. https://doi.org/10.26452/ijrps.v11iSPL4.4102
210. Shayeb MAL, Fathy E, Nadeem G, et al. Prevalence of most common tongue lesions among a group of uae population: Retrospective study. Onkologia i Radioterapia. 2020;14:1-5.
211. Izzetti R. Prevalence of oral and maxillofacial diseases in an Italian population: retrospective study on clinical and pathological features. Annali di Stomatologia, suppl Supplement 1. 2017;8:11-12.
212. Nandhini T, Narayan V. Prevalence of oral cancer among patients using different forms of tobacco - A retrospective study. Drug Invention Today. 2019;11:488-491.
213. Singh S, Singh J, Chandra S, et al. Prevalence of oral cancer and oral epithelial dysplasia among North Indian population: A retrospective institutional study. J Oral Maxillofac Pathol. 2020;24:87-92. https://doi.org/10.4103/jomfp.JOMFP_347_19
214. Chher T, Hak S, Kallarakkal TG, et al. Prevalence of oral cancer, oral potentially malignant disorders and other oral mucosal lesions in Cambodia. Ethnicity & health. 2018;23:1-15. https://doi.org/10.1080/13557858.2016.1246431
215. Zimmer J, Rigo Garbin R, Trapp Vogel M, Rigo L. Prevalence of Oral Lesions Diagnosed at a Pathology Institute: A Four-year Analysis. Pesquisa Brasileira em Odontopediatria e Clinica Integrada. 2024;24:1-7. https://doi.org/10.1590/pboci.2024.004
216. Martins JC, Essvein G, Vargas-Ferreira F, et al. Prevalence of oral lesions diagnosed at the ULBRA Canoas of Dental Diagnosis Service. Stomatos. 2017;23:24-32. http://www.periodicos.ulbra.br/index.php/stomatos/article/view/3052/2360
217. Ayyagari KR, Kumari NR, Bolem P, et al. Prevalence of Oral Lesions in Relation to Tobacco and Alcohol Habits Using VELscope - A Retrospective Study. Indian J Dent Res. 2024;35:412-416. https://doi.org/10.4103/ijdr.ijdr_514_24
218. Pannu SJ, Jain G, Pandi A, Narasimha VL, Varshney S. Prevalence of oral lesions in tobacco and alcohol users. Indian Journal of Cancer. 2021;58:S50.
219. Sassi LM, Dissenha JL, Guebur MI, et al. Prevalence of oral leukoplakia in oral cancer prevention in the state of Paraná-Brazil between 1989 and 2017. International Journal of Oral and Maxillofacial Surgery. 2019;48:242. https://doi.org/10.1016/j.ijom.2019.03.743
220. Devi S, Duraisamy R. Prevalence of oral lichen planus and assessment of factors associated with it-a retrospective study. Indian Journal of Forensic Medicine and Toxicology. 2020;14:5938-5946. https://doi.org/10.37506/ijfmt.v14i4.12533
221. Ravichandiran R, Venkatesh J, Felix AJW, et al. Prevalence of Oral mucosal lesion in patients with Tobacco related habits among Chidambaram population – A Cross-sectional study. Research Journal of Pharmacy and Technology. 2022;15:3377-3381. https://doi.org/10.52711/0974-360X.2022.00565
222. Karthik R, Mohan N. Prevalence of oral mucosal lesions among dental patients with mixed habits in Salem district - A study. Journal of Pharmacy and Bioallied Sciences. 2017;9:S55-S67. https://doi.org/10.4103/jpbs.JPBS_86_17
223. Rohini S, Sherlin HJ, Jayaraj G. Prevalence of oral mucosal lesions among elderly population in Chennai: a survey. Journal of Oral Medicine and Oral Surgery. 2020;26:10. https://doi.org/10.1051/mbcb/2020003
224. Hallikeri K, Naikmasur V, Guttal K, et al. Prevalence of oral mucosal lesions among smokeless tobacco usage: A cross-sectional study. Indian Journal Of Cancer. 2018;55:404-409. https://doi.org/10.4103/ijc.IJC_178_18
225. Joshi M, Pujara P, Sakaria P. Prevalence of Oral Mucosal Lesions among Tobacco Consumers: A Cross-Sectional Study. International Journal of Pharmaceutical and Clinical Research. 2024;16(3):1402-1406.
226. Singh G, Preethi B, Chaitanya KK, Navyasree M, Kumar TG, Kaushik MS. Prevalence of Oral Mucosal Lesions among Tobacco Consumers: Cross-Sectional Study. Journal Of Pharmacy And Bioallied Sciences. 2023;15:S562-S565. https://doi.org/10.4103/jpbs.jpbs_104_23
227. Sarumathi T, Mahalakshmi K, Jayesh R. Prevalence of oral mucosal lesions among tobacco users - A cross-sectional study. Drug Invention Today. 2019;11:1330-1333.
228. Pontes CC, Chikte U, Kimmie-dhansay F, et al. Prevalence of oral mucosal lesions and relation to serum cotinine levels—findings from a cross- sectional study in South Africa. International Journal of Environmental Research and Public Health. 2020;17:1065. https://doi.org/10.3390/ijerph17031065
229. Intapa C, Ayudhya CCN, Puangsombat A, et al. Prevalence of oral mucosal lesions in geriatric patients living in lower Northern Thailand: A 10 years retrospective study. Journal of International Dental and Medical Research. 2017;10:868-871.
230. Geetanjali D, Koppera N, Reddy RS, et al. Prevalence of Oral Mucosal Lesions in Geriatric Population of Coastal Andhra Pradesh. Oral & Maxillofacial Pathology Journal. 2023;14:180-184.
231. Sandhu ASU, Singh S, Sandhu KS, et al. Prevalence Of Oral Mucosal Lesions In Punjab, India. Journal of Population Therapeutics and Clinical Pharmacology. 2024;31:1400-1406. https://doi.org/10.53555/dv271r48
232. Santosh SS, Kumar J. Prevalence of oral mucosal lesions in Saveetha Dental College: A retrospective study. Research Journal of Pharmaceutical, Biological and Chemical Sciences. 2017;8:239-242.
233. Głowacka B, Konopka T. Prevalence of oral mucosal lesions in young seniors in the Wrocław region. Dental and Medical Problems. 2018;55:405-410. https://doi.org/10.17219/dmp/97310
234. Sidhu S, Katoch V, Premlata, Sharma P. Prevalence of oral mucosal lesions: A prospective study. International Journal of Dentistry and Oral Science. 2021;8:3682-3685.
235. Oivio UM, Pesonen P, Ylipalosaari M, Kullaa A, Salo T. Prevalence of oral mucosal normal variations and lesions in a middle-aged population: a Northern Finland Birth Cohort 1966 study. BMC Oral Health. 2020;20:357. https://doi.org/10.1186/s12903-020-01351-9
236. Nethan ST, Kumar V, Hariprasad R, et al. Prevalence of oral potentially malignant and malignant lesions and tobacco use among the older adults attending a screening clinic in noida (india): a cross-sectional study. Journal of Geriatric Oncology. 2019;10:S124-S125. https://doi.org/10.1016/S1879-4068(19)31326-8
237. Shivakumar KM, Raje V, Kadashetti V. Prevalence of oral potentially malignant disorders (OPMD) in adults of Western Maharashtra, India: A cross-sectional study. Journal Of Cancer Research And Therapeutics. 2022;18:S239-S243. https://doi.org/10.4103/jcrt.JCRT_1444_20
238. Sekizhar V, Ezhumalai G, Chanthrakumar C. Prevalence of Oral Potentially Malignant Disorders among Fishermen Population in and around Pondicherry, South India - A Cross Sectional Study. Indian Journal Of Occupational And Environmental Medicine. 2023;27:226-228. https://doi.org/10.4103/ijoem.ijoem_255_22
239. Pandya D, Banerjee A, Maitra A, et al. Prevalence of Oral Potentially Malignant Disorders Among Tobacco Users in Kolkata: A Hospital-Based Study. Cureus. 2024;16:e72084. https://doi.org/10.7759/cureus.72084
240. Sivakumar TT, Sam N, Joseph AP. Prevalence of oral potentially malignant disorders and oral malignant lesions: A population-based study in a municipal town of southern Kerala. J Oral Maxillofac Pathol. 2018;22:413-414. https://doi.org/10.4103/jomfp.JOMFP_202_17
241. Paulose S, Rangdhol V, Kavya L, et al. Prevalence of oral potentially malignant disorders associated with habits in Puducherry-A Cross-sectional study. Brazilian Journal of Oral Sciences. 2020;19. https://doi.org/10.20396/BJOS.V19I0.8658223
242. Khanna D, Tulika S, Tiwari M, et al. Prevalence of Oral Potentially Malignant Lesions, Tobacco use, and Effect of Cessation Strategies among Solid Waste Management workers in Northern India: a pre-post intervention study. BMC Oral Health. 2024;24:1-13. https://doi.org/10.1186/s12903-024-05087-8
243. Roy D, Datta B, Pathak B. Prevalence of Oral Precancerous Lesions in Tobacco and Areca Nut Habituated Patients in Barpeta District, Assam, India: A Cross-sectional Study. Journal Of Clinical And Diagnostic Research. 2022;16(12):ZC21-ZC26. https://doi.org/10.7860/JCDR/2022/60168.17310
244. Srivastava R, Sharma L, Pradhan D, et al O. Prevalence of oral premalignant lesions and conditions among the population of Kanpur City, India: A cross-sectional study. Journal Of Family Medicine And Primary Care. 2020;9:1080-1085. https://doi.org/10.4103/jfmpc.jfmpc_912_19
245. Pahwa V, Nair S, Shetty RS, et al. Prevalence of oral premalignant lesions and its risk factors among the adult population in Udupi taluk of coastal Karnataka, India. Asian Pacific Journal of Cancer Prevention. 2018;19:2165-2170. https://doi.org/10.22034/APJCP.2018.19.8.2165
246. Dirschnabel AJ, Pitt J, Kafer F, et al. Prevalence Of Oral Squamous Cell Carcinoma And Oral Potentially Malignant Lesions Diagnosed In Santa Catarina State. Oral Surgery, Oral Medicine, Oral Pathology & Oral Radiology. 2020;129:e179-e179. https://doi.org/10.1016/j.oooo.2019.06.759
247. Albert D, Muthusekhar MR, Selvarasu K. Prevalence of oral submucous fibrosis among tobacco users reporting to a private dental college in chennai: A retrospective study. International Journal of Pharmaceutical Research. 2021;13:1165-1171. https://doi.org/10.31838/ijpr/2021.13.01.188
248. Selvakumar S, Natarajan SK, Rajagopalan V, et al. Prevalence Of Oral Submucous Fibrosis In Construction Workers. Turkish Journal of Physiotherapy Rehabilitation. 2021;32:1324-1327.
249. Amin DS, Ahmed DR, Banday DM. Prevalence of oral submucous fibrosis in patients visiting dental college: A cross-sectional study. European Journal of Molecular and Clinical Medicine. 2022;9:5436-5444.
250. Rajendran R, Raju GK, Nair SM, et al. Prevalence of oral submucous fibrosis in the high natural radiation belt of Kerala, south India. Bull World Health Organ. 1992;70:783-789.
251. Jayaswal A, Goel S, Verma K, Jivrajani S, Makhijani B. Prevalence of oral submucous fibrosis linking with Areca Nut usage among Indians. Bioinformation. 2024;20:751-753. https://doi.org/10.6026/973206300200751
252. Sowmya S, Sangavi R. Prevalence of Oral Submucous Fibrosis With Other Oral Potentially Malignant Disorders: A Clinical Retrospective Study. Cureus. 2023;15: e49642. https://doi.org/10.7759/cureus.49642
253. Rani P, Singh RNP, Sharma S, et al. Prevalence of Oral Submucous Fibrosis, Its Correlation of Clinical Grading to Various Habit Factors among Patients of Bihar: A Cross Sectional Study. J Pharm Bioallied Sci. 2023;15:S554-s557. https://doi.org/10.4103/jpbs.jpbs_467_22
254. Kamala KA, Sankethguddad S, Nayak AG, et al. Prevalence of oromucosal lesions in relation to tobacco habit among a Western Maharashtra population. Indian J Cancer. 2019;56:15-18. https://doi.org/10.4103/ijc.IJC_231_17
255. Behera A, Abilasha R, Ramani P, et al. Prevalence of osmf cases in various age groups correlating with habits and treatment outcomes in saveetha dental college: A prevalence study. International Journal of Research in Pharmaceutical Sciences. 2020;11:1799-1804. https://doi.org/10.26452/ijrps.v11iSPL3.3519
256. Shaikh H, Bakerywala A, Razdan O, et al. Prevalence of Potentially Malignant Disorders in Tobacco Consuming Population: A Cross-Sectional Analysis. Journal of Pharmacy and Bioallied Sciences. 2024;16:S2794-S2796. https://doi.org/10.4103/jpbs.jpbs_376_24
257. George T, Varma PRP. Prevalence of potentially malignant oral disorders among migrant labourers. India Migration Report 2021. 2022:117-136. https://doi.org/10.4324/9781003287667-10
258. Benley G, Shibu S, Soman R, Mulamoottil V, Minimol J. Prevalence of precancerous lesions in an adult population. Indian Journal of Dental Research. 2019;30:500-505. https://doi.org/10.4103/ijdr.IJDR_138_18
259. Kumar BP, Narra P, Devi VV, et al. Prevalence of Premalignant Conditions and Their Transformation Into Oral Cancers: A Clinical Study. Journal of Pharmacy and Bioallied Sciences. 2024;16:S2563-S2565. https://doi.org/10.4103/jpbs.jpbs_384_24
260. Francis DL. Prevalence of premalignant lesions and oral cancer among tobacco-using tea plantation workers of Nilgiri Hills, Tamilnadu, India. Annals of Oncology. 2020;31:S1351-S1352. https://doi.org/10.1016/j.annonc.2020.10.275
261. Liu YD, He M, Yin T, et al. Prevalence of recurrent aphthous stomatitis, oral submucosal fibrosis and oral leukoplakia in doctor/nurse and police officer population. BMC Oral Health. 2022;22:353. https://doi.org/10.1186/s12903-022-02382-0
262. Neha K, Kunal O, Neha G, et al. Prevalence of smokeless tobacco use and oral pre-malignant lesions among heavy load truck drivers and general male population in Mumbai, India. Tobacco Induced Diseases. 2021;19 A100. https://doi.org/10.18332/tid/141034
263. Muralidharan S, Acharya A, Sevekari T, et al. Prevalence of Soft-Tissue Lesions among Women in Sex Work in the Red Light Area of Pune, India: A Cross-Sectional Survey. J Int Soc Prev Community Dent. 2018;8:218-223. https://doi.org/10.4103/jispcd.JISPCD_46_18
264. Sumithrarachchi S, Athukorala I, Rumy F, et al. Prevalence of tobacco and areca-nut use among patients attending dental teaching hospital in the central province of Sri Lanka and its association with oral mucosal lesions; a cross sectional study. J Oral Biol Craniofac Res. 2024;14:39-43. https://doi.org/10.1016/j.jobcr.2023.11.006
265. Divyadarshini V, Uma Maheswari TN. Prevalence Of Tobacco Associated Oral Mucosal Lesions In A Private Hospital-A Retrospective Study. Journal of Population Therapeutics and Clinical Pharmacology. 2023;30:19-27. https://doi.org/10.47750/jptcp.2023.30.06.004
266. Koothati RK, Raju DR, Prasad CLK, et al. Prevalence of tobacco associated oral mucosal lesions in the population of Mahabubnagar District of Telangana State: A cross-sectional study. Journal Of Indian Academy Of Oral Medicine And Radiology. 2020;32:149-153. https://doi.org/10.4103/jiaomr.jiaomr_36_20
267. Fernandes ME, B. Sikkerimath S. Prevalence of Tobacco Habit and Associated Oral Lesions. Rajiv Gandhi University of Health Sciences (India); 2019. https://www.proquest.com/dissertations-theses/prevalence-tobacco-habit-associated-oral-lesions/docview/2866353915/se-2?accountid=26642
268. Sowmya GS, Vadivel JK, Malaiappan S. Prevalence of tobacco usage in oral leukoplakia: A retrospective study. International Journal of Research in Pharmaceutical Sciences. 2020;11:1896-1901. https://doi.org/10.26452/ijrps.v11iSPL3.3583
269. Francis DL, Saravanan SP. Prevalence of Tobacco Use and Oral Malignant Precancerous Lesions Among Auto Rickshaw Drivers in Chennai City, Tamil Nadu, India. JCO Global Oncology. 2024;10:150. https://doi.org/10.1200/GO-24-11900
270. Rajkuwar A, Verma A, Vijayapandian H, et al. Prevalence of Tobacco Use and Oral Mucosal Lesions among Nicobarese Tribal Population in Andaman and Nicobar Islands. Journal of Contemporary Dental Practice. 2021;22:975-978. https://doi.org/10.5005/jp-journals-10024-3176
271. Kalliath JD, Agrawal T. Prevalence of Tobacco Use and Oral Precancerous Lesions, and Their Associated Factors Among Adult Females in Rural Areas of a Primary Health Centre in Bangalore Urban District. Rajiv Gandhi University of Health Sciences (India); 2018. https://www.proquest.com/dissertations-theses/prevalence-tobacco-use-oral-precancerous-lesions/docview/2866356025/se-2?accountid=26642
272. Khan B, Inamdar IF, Gattani PL, et al. Prevalence of tobacco use, nicotine dependence and associated oral premalignant lesion in rural area of Nanded District of Maharashtra, India. Journal of Cardiovascular Disease Research. 2023;14:1524-1530. https://doi.org/10.48047/jcdr.2023.14.09.163
273. Francis DL. Prevalence of tobacco use, potentially malignant lesions and oral cancer among Irula tribes, Nilgiri hills, Tamilnadu, India. Cancer Epidemiology Biomarkers and Prevention. 2023;32. https://doi.org/10.1158/1538-7755.DISP22-C128
274. Choudhary A, Kesarwani P, Chakrabarty S, et al. Prevalence of tobacco-associated oral mucosal lesion in Hazaribagh population: A cross-sectional study. Journal Of Family Medicine And Primary Care. 2022;11:4705-4710. https://doi.org/10.4103/jfmpc.jfmpc_1990_21
275. Hafeez N, Maheshwari TNU. Prevalence of type of chewing tobacco associated with osmf in patients reported in a private dental institution: a retrospective study. International journal of early childhood special education. 2022;14:9535-9544. https://doi.org/10.9756/INT-JECSE/V14I3.1092
276. Melo IGG, Vaz FFS, Sobrinho ARS, et al. Prevalência da queilite actínica em agricultores de uma região do sertão brasileiro. Rev cuba estomatol. 2021;58:e3354-e3354.
277. Alves PM, Gomes DQ de C, Pereira JV. Prevalência das lesões cancerizáveis na cavidade oral no Município de Campina Grande - Paraíba - Brasil. Rev bras ciênc saúde. 2004:247-254.
278. Tejada García A. Prevalencia de lesiones bucales en tejido blando encontradas en la Clínica de Estomatología de la Facultad de Odontología de la Universidad de los Andes: periodo 2015-2018. Rev ADM. 2020;77:11-16. https://doi.org/10.35366/OD201C
279. Signorini CV, Navarrete-Marabini N, Casañas-Gil E, et al. Prevalencia de lesiones de la mucosa oral en pacientes que acuden a una clínica universitaria. Estudio transversal retrospectivo a 5 años. Piel. 2024;39:193-200.
280. Ortiz AAC, Carrillo AT. Prevalencia de lesiones en mucosa bucal y su relación con condición sistémica, edad y sexo. Rev ADM. 2024;81:26-38. https://doi.org/10.35366/114744
281. Cardoso IL, Azul AMM, Felino ACC. Prevalência de lesões da Mucosa Oral Numa População Idosa da Região Norte de Portugal. Universidade do Porto (Portugal); 2018. https://www.proquest.com/dissertations-theses/prevalência-de-lesões-da-mucosa-oral-numa/docview/3039728844/se-2?accountid=26642
282. da Conceição PA, Lopes TT, Correia A. Prevalência e Caracterização de Lesões Orais Brancas na População Idosa Institucionalizada. Universidade Catolica Portuguesa (Portugal); 2018. https://www.proquest.com/dissertations-theses/prevalência-e-caracterização-de-lesões-orais/docview/2925086729/se-2?accountid=26642 01&volume=&issue=&spage=&au=da+Concei%C3%A7%C3%A3o%2C+Pedro+Almeida&isbn=9798381523874&jtitle=&btitle=&rft_id=info:eric/&rft_id=info:doi/
283. Santos FBG, Leonhardt FD, Abrahao M. Prevention of upper aerodigestive tract cancer through active search strategies and use of equipped propaedeutics. Brazilian Journal Of Otorhinolaryngology. 2020;86:443-449. https://doi.org/10.1016/j.bjorl.2019.01.002
284. Rampi PT, Gambin DJ, Leal LO, et al. Profile of Patients with White Lesions of the Oral Mucosa Treated at a Dental School in Southern Brazil. Journal Of Clinical And Diagnostic Research. 2019;13:ZC05-ZC09. https://doi.org/10.7860/JCDR/2019/42404.13314
285. Costa CA, Toro CG, Hanna AVL. Queilitis actinica en pescadores artesanales de la v region : su prevalencia y aspectos histopatologicos asociados. Published online 1987. https://repositoriobibliotecas.uv.cl/handle/uvscl/12549
286. Pérez González A. Relación entre los comportamientos de riesgo para la salud, los desórdenes orales potencialmente malignos y el estado de salud oral. Published online 2024.
287. Agarwal N, Shaikh MN, Banu A, et al. Repercussions of Smokeless Tobacco on Buccal Mucosa: A Community Based Observational Study at a Tertiary Care Centre in Western Rajasthan. Indian Journal of Otolaryngology and Head and Neck Surgery. 2024;76:1891-1897. https://doi.org/10.1007/s12070-023-04440-7
288. Kakoei S, Torabi M, Rad M, et al. Retrospective Study of Oral Lichen Planus and Oral Lichenoid Lesions: Clinical Profile and Malignant Transformation. Journal of Dentistry. 2022;23:452-458. https://doi.org/10.30476/DENTJODS.2021.91356.1572
289. Rimal J, Shrestha A, Maharjan IK, et al. Risk assessment of smokeless tobacco among oral precancer and cancer patients in eastern developmental region of Nepal. Asian Pacific Journal of Cancer Prevention. 2019;20:411-415. https://doi.org/10.31557/APJCP.2019.20.2.411
290. Shah N, Shah Y, Supeda D, et al. Role of Tobacco Consumption Habits in the Causation of Precancerous Lesions: A Cross Sectional Study. Medicina Moderna. 2023;30:53-56. https://doi.org/10.31689/RMM.2023.30.1.53
291. Lalli A, Aldehlawi H, Buchanan JAG, et al. Screening for oral cancer utilising risk-factor analysis is ineffective in high-risk populations. Br J Oral Maxillofac Surg. 2021;59:e17-e22. https://doi.org/10.1016/j.bjoms.2020.08.094
292. Meena J, Verma A, Upadhyay S. Screening of oral cancer pathology and risk factors at primary care level in Jodhpur, India. Supportive Care in Cancer. 2019;27:S147-S148. https://doi.org/10.1007/s00520-019-04813-1
293. Nagao T, Fukuta J, Kurita K, et al. Site specific prevalence of oral cancer and potentially malignant disorders among Japanese cigarette smokers. Head and Neck. 2017;39:E78.
294. Schell H, Schönberger A. Site-specific incidence of benign and precancerous leukoplakias and cancers of the oral cavity. Z Hautkr. 1987;62:798-804.
295. Mishra GA, Pimple SA, Gupta SD. Smokeless tobacco use and oral neoplasia among urban Indian women. Oral Diseases. 2019;25:1724-1734. https://doi.org/10.1111/odi.13166
296. Moreira P, Assaf AV, Cortellazzi KL, et al. Social and behavioural associated factors of actinic cheilitis in rural workers. Oral Dis. 2021;27:911-918. https://doi.org/10.1111/odi.13610
297. Bansal S, Shaikh S, Desai RS, et al. Spectrum of Lip Lesions in a Tertiary Care Hospital: An Epidemiological Study of 3009 Indian Patients. Indian Dermatol Online J. 2017;8:115-119. https://doi.org/10.4103/2229-5178.202280
298. Fattori E, Teixeira DS, de Figueiredo MA, et al. Stomatological disorders in older people: An epidemiological study in the Brazil southern. Med Oral Patol Oral Cir Bucal. 2019;24:e577-e582. https://doi.org/10.4317/medoral.22966
299. Munde A, Harihar L, Vadvadgi V, et al. Study of assessment of prevalence and patterns of tobacco use and tobacco induced oral lesions in rural population of Western Maharashtra: An epidemiological study. Pravara Medical Review. 2021;13:14-24. https://doi.org/10.36848/PMR/2020/25100.51005
300. Das AK, Baishya N, Jha RK, et al. Study of association of premalignant lesion of oral cavity with the use of areca nut in the state of Assam, India. Head and Neck. 2017;39:E195.
301. Kumar P, Sehgal K. Study on clinicopathological spectrum of oral lesions at our tertiary care hospital. Journal of Cardiovascular Disease Research. 2024;15:342-346. https://doi.org/10.48047/jcdr.2024.15.01.38
302. Morikawa T, Bessho H, Yakushiji T, et al. The activity report of the oral cancer screening in Tokyo dental college: -Mass screening of the oral cancer screening in all area and individual screening in Chiba-city. Japanese Journal of Head and Neck Cancer. 2018;44:353-360. https://doi.org/10.5981/JJHNC.44.353
303. Radochová V, Koberová Ivančaková R, et al. The Characteristics of Patients with Oral Lichen Planus and Malignant Transformation-A Retrospective Study of 271 Patients. Int J Environ Res Public Health. 2021;18: 6525. https://doi.org/10.3390/ijerph18126525
304. Barreto ACR. The clinical prevalence of actinic cheilitis among community health agents from the regional V subprefecture of Fortaleza. RGO. 2017;65:128-133. https://doi.org/10.1590/1981-863720170002000053148
305. Ahern J, Toner M, Van Harten M, et al. The detection of oral cancer and potentially malignant disorders in Ireland: An observational study of 100 cases. J Public Health Dent. 2020;80:333-337. https://doi.org/10.1111/jphd.12373
306. Ghosh R, Ray D, Sarangi S, et al. The Effects of Tobacco Use in Oral Cavity and Periodontal Health in Patients of Burdwan-An Observational Study. International Journal of Current Pharmaceutical Review and Research. 2025;17:14-21.
307. Miller TE, Dickens NE, Rehmel MR, Jones BE, Hamlin NJ, Robbins QC. The Impact of a Pandemic on a Military Oral and Maxillofacial Pathology Biopsy Service. Military Medicine. 2024;189:e1797-e1804. https://doi.org/10.1093/milmed/usae102
308. Kaur A, Chauhan NS, Shivakumar S. The Predominance of Tobacco Propensities and Tobacco-Related Oral Lesions in Textile Mill Workers of Bhopal: A Cross-Sectional Study. Cureus. 2023;15. https://doi.org/10.7759/cureus.41085
309. Worakhajit P, Fuangtharnthip P, Khovidhunkit SP et al. The Relationship of Tobacco, Alcohol, and Betel Quid with the Formation of Oral Potentially Malignant Disorders: A Community-Based Study from Northeastern Thailand. International Journal of Environmental Research and Public Health. 2021;18:8738. https://doi.org/10.3390/ijerph18168738
310. Araújo ALD, Fonseca JM, do Amaral-Silva GK, et al. The Role of Immunohistochemistry for Primary Oral Diagnosis in a Brazilian Oral Pathology Service. APPLIED Immunohistochemistry & Molecular Morphology. 2021;29:781-789. https://doi.org/10.1097/PAI.0000000000000960
311. Reddy PA, Wahab PUA, Jagadish V. To assess the awareness of pre-malignant conditions in diagnosed cancer patients in private university hospital. European Journal of Molecular and Clinical Medicine. 2020;7:585-590.
312. Anjum B, Singh G, Pappu S, et al. To evaluate the level of awareness of patients about existing oral precancerous lesions: A longitudinal study. Journal of Pharmacy and Bioallied Sciences. 2023;15:566-570. https://doi.org/10.4103/jpbs.jpbs_31_23
313. Nurmukhambetova BK, Tankibayeva ZG, Akhmetova SB, et al. To the question of the prevalence of oral mucosal lesions in central Kazakhstan. Minerva Medica. 2019;110:83-84.
314. Ali AKS, Mohammed A, Thomas AA, et al. Tobacco abuse and associated oral lesions among interstate migrant construction workers. Journal of Contemporary Dental Practice. 2017;18:695-699. https://doi.org/10.5005/jp-journals-10024-2109
315. Meshram N, Bhandarkar A, Meshram R, et al. Tobacco addiction and it’s consequences on oral health of indigenous and rural people - A cross sectional study from central India. Tobacco Induced Diseases. 2018;16:347. https://doi.org/10.18332/tid/84341
316. Aparnaa M, Sherlin HJ. Tobacco and betel nut chewing behaviour and its association with potentially malignant disorders in Chennai. Indian Journal of Public Health Research and Development. 2019;10:130-135. https://doi.org/10.5958/0976-5506.2019.03438.7
317. Palliyal S. Tobacco chewing habits and risk of precancerous oral lesions among Paniya tribes of Wayanad, India - a cross sectional study. Tobacco Induced Diseases, suppl 17th World Conference on Tobacco or Health, WCTOH. 2018;16: A377. https://doi.org/10.18332/tid/83963
318. Kommalapati R, Rajendra AB, Kattappagari K, et al. Tobacco Related Oral Lesions in South Indian Industrial Workers. Journal of Orofacial Sciences. 2021;13:28-32. https://doi.org/10.4103/jofs.jofs_24_21
319. Shajahan A, Mathew AC, Gangadharan VP, et al. Tobacco use and clinical leukoplakia lesions among south Indian tribes. Journal of Basic & Clinical Physiology & Pharmacology. 2023;34:647-654. https://doi.org/10.1515/jbcpp-2020-0347
320. Silveira ML, Everard CD, Sharma E, et al. Tobacco Use and Incidence of Adverse Oral Health Outcomes Among US Adults in the Population Assessment of Tobacco and Health Study. JAMA Network Open. 2022;5: e2245909. https://doi.org/10.1001/jamanetworkopen.2022.45909
321. Patil AP, Yogeshkumar S. Tobacco use and oral premalignant lesions among auto-rickshaw drivers in Belagavi, North Karnataka. Indian Journal Of Occupational And Environmental Medicine. 2023;27:79-83. https://doi.org/10.4103/ijoem.ijoem_163_22
322. Francis DL. Tobacco use and prevalence of oral premalignant lesions, among Malayali tribes, Yelagiri Hills, Tamil nadu, India. Cancer Epidemiology Biomarkers and Prevention. 2024;33:A092. https://doi.org/10.1158/1538-7755.DISP24-A092
323. Francis DL. Tobacco use, awareness and oral health among kanchipuram silk weavers, Tamil Nadu, India. Journal of Thoracic Oncology. 2017;12:S2294.
324. Madhu PP, Quazi ZS, Wankhede AN. Tobacco Use, Body Mass Index, Potentially Malignant Disorder and Attitude towards Passive Smoking in a Primary Health Centre – A Cross‑Sectional Study. Journal of Pharmacy and Bioallied Sciences. 2024;16:S3982-S3985. https://doi.org/10.4103/jpbs.jpbs_888_24
325. Yunus GY, Sahni H, Naveen N, et al. “Tobacco” - The Silent Slayer for Oral Premalignant Lesions/Conditions among Beedi Rolling Workers of Durg City, Chhattisgarh, India: A Cross-Sectional Study. Journal Of Indian Association Of Public Health Dentistry. 2019;17:119-124. https://doi.org/10.4103/jiaphd.jiaphd_215_18
326. Al-Wesabi MA, Al-Hajri M, Shamala A, et al. Tongue lesions and anomalies in a sample of Yemeni dental patients: a cross-sectional study. J oral res (Impresa). 2017;6:121-126. https://doi.org/10.17126/joralres.2017.038
327. Anirudh BVM, Sakthi S, Sudarrshiny S. Type of tobacco used and its associated risk for premalignant lesions in young adolescents visiting a private dental hospital in chennai. International journal of early childhood special education. 2022;14:2082-2090. https://doi.org/10.9756/INT-JECSE/V14I2.075
328. Coelho HJR, Macedo JC, Silami MANC. Uma análise retrospectiva de lesões malignas e desordens potencialmente malignas em sete anos. Rev nav odontol. 2019;46:5-13. https://doi.org/10.29327/25149.46.1-2
329. Al-Maweri SA, Al-Jamaei A, Saini R, et al. White oral mucosal lesions among the Yemeni population and their relation to local oral habits. Journal of investigative and clinical dentistry. 2018;9:e12305. https://doi.org/10.1111/jicd.12305
330. Tirukkovalluri SS, Luck CP, Makesh RLS, et al. Workplace based potentially malignant oral lesions screening among tobacco consuming migrant construction site workers in Chennai, South India: A pilot study. Journal Of Family Medicine And Primary Care. 2020;9:5004-5009. https://doi.org/10.4103/jfmpc.jfmpc_687_20
