# Supplementary material for: Global Prevalence of Oral Potentially Malignant Disorders: An Updated Systematic Review and Meta‐Analysis
Source: J Oral Pathol Med. 2026 Apr 28;55(7):747–54. doi: 10.1111/jop.70146 (PMC13429371; doi:10.1111/jop.70146)
Supplement: Supplementary file 3 — Appendix S3: Extracted data from included studies. [file JOP-55-747-s012.docx]

**Appendix S3.** Summary of the included studies (n=108)

| PREVIOUS SYSTEMATIC REVIEW (N=22) | | | | | | | |
| --- | --- | --- | --- | --- | --- | --- | --- |
| Author, year, Country  Study design | Type of lesion (n) | Total Sample (n) | Gender (positive cases, n) | Age (n, range, mean) in years | Etiologic factors (n) | Histopathological diagnosis (n) | Prevalence (%) |
| Amarasinghe et al., 2010, Sri Lanka. Descriptive^1^ | **OPMD (96)**  OL (71)  - HOL (66)  - NHOL (5)  OSMF (25) | 1029 | M (66)  F (30) | NR | Betel-quid chewing (97)  No chewing (4)  Alcohol drinking (62)  No alcohol drinking (39)  Smoking (35)  No smoking (66) | NR | **OPMD=9.33**  OL=6.90  - HOL=6.41  - NHOL=0.49  OSMF=2.43 |
| Campisi and Margiotta, 2001, Italy. Descriptive^2^ | **OPMD (20)**  OL (15)  AC (5) | 118 | NR | NR | Smoking only (3)  Drinking only (3)  Smoking/Drinking (14)  Solar exposure (5=AC) | AC (5)  MoED (2) | **OPMD=16.95**  OL=12.71  AC=4.24 |
| Cecotti et al., 1997,  Argentina. Descriptive^3^ | OL (8) | 267 | M (5)  F (3) | 39-80  Mean (50.8) | NR | NR | OL=3.00 |
| Femopase et al., 1997, Argentina. Descriptive^4^ | OL (418)  - HOL (392)  - NHOL (26) | 9021 | M (309)  F (109) | 18-83  Mean (45.28) | NR | NR | OL=4.63  - HOL=4.35  - NHOL=0.29 |
| García et al., 1997,  Spain. Descriptive^5^ | OL (14)  - HOL (11)  - NHOL (3) | 4000 | M (8)  F (6) | 35-70 (9)  >81 (1) | Smoking (5) | No dysplasia (8)  MiED (3)  MoED (1)  SED (2) | OL=0.35  - HOL=0.27  - NHOL=0.07 |
| Haas Junior et al., 2011, Brazil. Descriptive^6^ | OL (177) | 8635 | M (89)  F (88) | <50 (76)  ≥50 (97)  NR (4) | NR | EA (7)  HKA (40)  EA with EHP (4)  HK (39)  EHP (60)  ED (27) | OL=2.05 |
| Idris et al., 2016, Saudi Arabia, Descriptive^7^ | **OPMD (26)** | 714 | M (12)  F (14) | <50 (5)  51-60 (5)  >65 (12) | Shammah user (9)  Shammah nonuser (17) | MiED (12)  MoED (6)  SED (6)  No grading (2) | **OPMD=3.64** |
| Jahanbani, 2003,  Iran. Descriptive^8^ | OL (43) | 1167 | M (43)  F (0) | 25-58  Mean (37.6) | Smoking (33) | HK (43) | OL=3.68 |
| Kaugars et al., 1999,  USA. Descriptive^9^ | AC (150) | 66067 | M (122)  F (28)  NR (2) | Mean (61.8) | Tobacco (20)  Non-smokers (10)  Solar exposure (33) | MiED (79)  MoED (35)  SED (36) | AC=0.23 |
| Lapthanasupkul et al., 2007, Thailand. Descriptive^10^ | **OPMD (123)**  OL (117)  OE (6) | 7177 | M (74)  F (58) | OL  ≤50 (49)  >50 (70)  OE  ≤50 (3)  >50 (6) | NR | OL (123)  HK (75)  ED (13)  Others (29)  OE (9)  ED (3)  HK (1)  Others (2) | **OPMD=1.71**  OL=1.63  OE=0.08 |
| Mendez et al., 2012, Brazil. Descriptive^11^ | OL (137) | 6831 | M (70)  F (67) | 30-49 (20)  ≥50 (117) | NR | NR | OL=2.01 |
| Misra et al., 2009, India. Descriptive^12^ | **OPMD (146)**  OL (78)  OSMF (68) | 753 | NR | Mean  OL (35.49)  OSMF (35.19) | NR | NR | **OPMD=19.39**  OL=10.36  OSMF=9.03 |
| Ntomouchtsis et al., 2010, Greece. Descriptive^13^* | AC (24) | 140 | M (24) | 42-89 (15) | NR | NR | AC=17.14 |
| Pentenero et al., 2008, Italy. Descriptive^14^ | OL (47) | 4098 | M (37)  F (10) | NR | Tobacco (38)  Alcohol (27)  Tobacco+Alcohol (21) | NR | OL=1.15 |
| Queiroz et al., 2014,  Brazil. Descriptive^15^ | **OPMD (49)**  OL (41)  OE (8) | 6560 | M (24)  F (30) | 23-86 (mean 56.9) | Smoking (39)  Non-smokers (15)  Drinking (7)  NR (47) | OL  HKA/MiED (37)  MoED/SED (4)  OE  HKA/MiED (3)  MoED/SED (5) | **OPMD=0.75**  OL=0.62  OE=0.12 |
| Sánchez et al., 2007, Cuba. Descriptive^16^ | **OPMD (87)**  OL (81)  OE (6) | 527 | NR | NR | NR | NR | **OPMD=16.51**  OL=15.37  OE=1.14 |
| Shafer and Waldron, 1975, USA. Descriptive^17^ | OE (32) | 64354 | M (15)  F (17) | NR | NR | MiED/MoED (6)  SED/CIS (26) | OE=0.05 |
| Silveira et al., 2009, Brazil. Descriptive^18^ | **OPMD (205)**  OL (152)  - HOL (145)  - NHOL (7)  AC (33)  OE (20) | 7725 | M (87)  F (118) | Mean  OPMD (54.47)  OL (53.99±15.1)  NHOL (60.29±14.5)  AC (51.64±14.2)  OE (60.63±15.1) | NR | **OL (110)**  HK (48)  ED (22)  HK + ED (8)  **AC (22)**  SE + EA (10)  SE + MiED (3)  SE +MoED (2)  SE + HK (2)  Only ED (5)  **OE (7)**  MiED (2)  MoED (1)  SED (1) | **OPMD=2.65**  OL=1.97  AC=0.43  OE=0.26 |
| Silverman et al., 1976,  India. Descriptive^19^ | OL (6753) | 57518 | M (6611)  F (107) | NR | Smoking alone (2768)  Smoking and “pan”/ “supari” chewing (2264)  Smoking and tobacco chewing (302)  Other habits (1270)  No habits (114) | HK (6718)  ED (35) | OL=11.74 |
| Souza, 2014, Brazil. Descriptive^20^ | OL (115) | 3991 | M (53)  F (62) | Mean 51.6 | NR | HKA (95)  MiED (13)  MoED (5)  SED (2) | OL=2.88 |
| Starzynska et al., 2014,  Poland. Descriptive^21^ | OL (320)  - HOL (147)  - NHOL (57)  - NI (116) | 55911 | M (100)  F (104) | ≤50 (18)  >50 (86)  (mean 58.1) | Alcohol (83)  Cigarettes (103) | Dysplasia (7)  No dysplasia (313) | OL=0.57  - HOL=0.26  - NHOL=0.10 |
| Yang et al., 2010,  Taiwan. Descriptive^22^ | **OPMD (316)**  OL (224)  OSMF (89)  OE (3) | 2020 | M (131)  F (185) | NR | NR | NR | **OPMD=15.64**  OL=11.09  OSMF=4.41  OE=0.15 |
| RETRIEVED STUDIES THAT WERE PREVIOUSLY EXCLUDED FROM THE SYSTEMATIC REVIEW DUE TO AGE CRITERIA (N=11) | | | | | | | |
| Author, year, Country  Study design | Type of lesion (n) | Total Sample (n) | Gender (positive cases, n) | Age (n, range, mean) in years | Etiologic factors (n) | Histopathological diagnosis (n) | Prevalence (%) |
| Casnati et al., 2013, Uruguay. Cross-seccional^23^ | OL (2998) | 44960 | M (1917) F (1081) | NR | NR | NR | OL= 6.67 |
| Daftary et al., 1978, India.^24^ | OL (48) | 20358 | NR | NR | NR | HOK (11)  HOPK (2)  HPK (1)  NK (1)  ED (1) | OL=0.24 |
| Elango et al., 2011, India. ¥^25^ | OL (20) | 34766 | NR | NR | NR | 20 (nED) | OL=0.06 |
| Ferreira et al., 2016, Brazil. ¥^26^ | **OPMD (126)**  AC (116)  OL (9)  OE (1) | 1385 | NR | NR | NR | NR | **OPMD=9.10**  AC=8.38  OL=0.65  OE=0.07 |
| Mehrotra et al., 2008, India. Retrospective. ¥^27^ | **OPMD (286)**  OSMF (196)  OL (90) | 1151 | M (211) F (75) | NR | NR | OSMF (196)  OL (90) | **OPMD=24.85**  OSMF=17.03  OL=7.82 |
| Moret et al., 2007, Venezuela. Retrospective.^28^ | **OPMD (349)**  OL (343)  AC (5)  OE (1) | 7000 | OL  M (142) F (201) | OL  0-9 (4)  10-19 (6)  20-29 (36)  30-39 (50)  40-49 (52)  50-59 (56)  60-69 (116)  70-79 (11)  >80 (12) | NR | NR | **OPMD=4.98**  OL=4.90  AC=0.07  OE=0.01 |
| Moret et al., 2008, Venezuela. Retrospective.^29^ | OL (180) | 11250 | M (78) F (102) | 50-59 (54) | Smoking (77) | MiED (97) MoED (67) SED (18) | OL=1.60 |
| Moret, 2014, Venezuela. Retrospective.^30^ | OL (764) | 4166 | NR | NR | NR | NR | OL=18.34 |
| Pindborg et al., 1971, India.^31^* | OL (497) | 10169 | M (208) F (289) | 15-24 (13)  25-34 (60)  35-44 (144)  45-54 (141)  55-64 (104)  >65 (35) | Smoking (471)  Reverse smokers (413)  Other (39)  Bidi (19)  Mixed (23)  No habits (3) | ED (76) | OL= 4.89 |
| Tang et al., 1997, China.^32^ | OSMF (101) | 11046 | NR | NR | NR | NR | OSMF=0.91 |
| Waldron et al., 1975, USA. ¥^33^ | OL (3256) | 52145 | M (1768) F (1488) | 1-10 (4)  11-20 (31)  21-30 (127)  31-40 (390)  41-50 (720)  51-60 (846)  61-70 (678)  71-80 (273)  81-90 (62)  91-100 (3)  NR (122) | NR | MiED/MoED (413) SED (153) | OL=6.24 |
| UPDATE SYSTEMATIC REVIEW 2017–2025 (N=75) | | | | | | | |
| Author, year, Country  Study design | Type of lesion (n) | Total Sample (n) | Gender (positive cases, n) | Age (n, range, mean) in years | Etiologic factors (n) | Histopathological diagnosis (n) | Prevalence (%) |
| Agrawal et al., 2021, India.  Cross-sectional.^34^ | OSMF (40) | 500 | M (35)  F (5) | 21-30 (20)  31-40 (15)  41-50 (5) | Guthka (40) | NR | OSMF= 8.00 |
| Ahern et al., 2019, Ireland. Retrospective.^35^ | **OPMD (94)** | 700 | NR | NR | NR | ED (94) | **OPMD= 13.43** |
| AlOsaimi et al., 2024, Saudi Arabia. Case control.^36^ | OSMF (1) | 136 | NR | NR | Smoking  Yes (1) | NR | OSMF= 0.74 |
| Anwar et al., 2023, India.  Cross-sectional. ¥^37^ | **OPMD (22)**  OSMF (11)  OL (6)  - NHOL (1)  - NI (5)  PVL (5) | 2620 | OSMF M (11) | NR | Alcohol (6)  Non-alcohol (16)  Bidi (9)  Non-bidi (13)  Cigarette (10)  Non-cigarette (12)  Gutkha (18)  Non-gutkha (4)  Pan (10)  Non-pan (12) | NR | **OPMD= 0.84**  OSMF= 0.42  OL= 0.23  - NHOL= 0.04  PVL= 0.19 |
| Armelin et al., 2019, Brazil.  Cross-sectional. ¥^38^ | **OPMD (20)**  OL (13)  OE (4)  AC (3) | 1282 | NR | NR | NR | NR | **OPMD= 1.56**  OL= 1.01  OE= 0.31  AC= 0.23 |
| Azevedo et al., 2021, Brazil.^39^ | **OPMD (953)**  OL (800)  - NHOL (89)  - NI (711)  AC (145)  OE (8) | 11833 | M (419)  F (534) | Mean±SD  59.7±13.58  Range (11-100) | Tobacco (277)  Non-tobacco (207)  Alcohol (136)  No-alcohol(212) | nED (401)  MiED (319)  MoED (139)  SED (94) | **OPMD= 8.05**  OL= 6.76  - NHOL=0.75  AC= 1.23  OE= 0.07 |
| Barros et al., 2020, Brazil. Retrospective.^40^* | **OPMD (152)**  AC (136)  OL (16) | 587 | AC  M (96) F (40)  OL  M (8) F (8) | AC  20-59 (92)  >60 (44)  OL  20-59 (7)  >60 (9) | NR | NR | **OPMD= 25.90**  AC= 23.20  OL = 2.70 |
| Bastakoti et al., 2021, Nepal. Cross-sectional. ¥^41^ | **OPMD (99)** | 851 | NR | NR | NR | ED (98)  VHP (1) | **OPMD= 11.63** |
| Bhattacharjee et al., 2025, India.^42^ | **OPMD (42)**  OSMF (22)  OL (17)  OE (3) | 841 | NR | NR | NR | NR | **OPMD= 4.99**  OSMF= 2.62  OL= 2.02  OE= 0.36 |
| Blochowiak et al., 2019, Poland. Retrospective. ¥^43^ | OL (6) | 208 | NR | NR | NR | NR | OL= 2.88 |
| Cesar et al., 2021, Brazil. Retrospective. ¥^44^ | **OPMD (37)**  AC (21)  OL (16) | 640 | NR | ≥60 (37) | NR | NR | **OPMD= 5.78**  AC= 3.28  OL= 2.50 |
| Cigic et al., 2023, Croatia.  Cross-sectional. ¥^45^ | **OPMD (9)**  OL (4)  OE (3)  AC (2) | 102 | M (8)  F (1) | Mean±SD  OL (56±8.29)  AC (45±1.31)  OE (51±1) | Alcohol (6)  Non-alcohol (2)  Smoking (6)  Non-smoking (2) | HK (4)  MoED (2)  SED (1)  AC (2) | **OPMD= 8.82**  OL= 3.92  OE= 2.94  AC= 1.96 |
| Chiu et al., 2021, Taiwan. Observational. ¥^46^ | **OPMD** (149913)  OL (108869)  PVL (24000)  OE (15007)  OSMF (2037) | 3362232 | NR | NR | NR | NR | **OPMD= 4.46**  OL= 3.24  PVL= 0.71  OE= 0.45  OSMF= 0.06 |
| Collins et al., 2021, Dominican Republic. ¥^47^ | **OPMD (15)**  OL (13)  AC (2) | 248 | NR | NR | NR | NR | **OPMD= 6.05**  OL= 5.24  AC= 0.81 |
| Cordero et al., 2020, Chile.  Cross-sectional. ¥^48^ | OL (1) | 161 | NR | NR | NR | NR | OL= 0.62 |
| Cunha et al., 2023, Brazil. Retrospective. ¥^49^ | **OPMD (759)**  OL, OE, NHOL (637)  AC (122) | 7476 | M (362) F (395)  NR (2) | Mean±SD  (69.78±7.74)  60-69 (431)  70-79 (223)  80-89 (96)  >90 (9) | NR | nED (32)  MiED (258)  MoED (153)  SED (194)  AC (122) | **OPMD= 10.15**  OL, OE, NHOL= 8.52  AC= 1.63 |
| Cury et al., 2024, Brazil.  Cross-sectional.^50^ | **OPMD (16)**  AC (14)  OL (2) | 295 | M (16)  F (0) | NR | NR | NR | **OPMD= 5.42**  AC= 4.75  OL= 0.68 |
| da Silva Arruda et al., 2021, Brazil. Cross-sectional.^51^ | **OPMD (4)**  OL (2)  AC (2) | 82 | M (3)  F (1) | NR | NR | NR | **OPMD= 4.88**  OL= 2.44  AC= 2.44 |
| Dabla et al., 2022, India. Cross-sectional. ¥^52^* | **OPMD (20)** | 169 | M (12) F (8) | 11-20 (7)  21-30 (6)  31-40 (3)  41-50 (2)  51-60 (2) | NR | MiED (17)  MoED (3) | **OPMD= 11.83** |
| de Almeida et al., 2022, Portugal. Observational.^53^ | OL (140) | 1448 | M (72)  F (68) | Mean±SD  (55.81±14.87) | NR | NR | OL= 9.67 |
| de Souza et al., 2024, Brazil. Cross-sectional.^54^* | AC (124) | 1284 | M (76)  F (48) | 0-9 (1)  10-19 (2)  20-59 (65)  ≥60 (46)  NR (10) | NR | NR | AC= 9.66 |
| Dhanuthai et al., 2020, Thailand. Multicenter ^55^* | **OPMD (183)** | 2153 | M (77) F (106) | Mean±SD (54.55±14.47) | NR | ED (169) SED (14) | **OPMD= 8.50** |
| Ferreira et al., 2024, Brazil. Retrospective.^56^ | **OPMD (42)**  AC (38)  OL (4) | 150 | NR | NR | NR | NR | **OPMD= 28.00**  AC=25.33  OL =2.67 |
| Gambino et al., 2017 Italy. Retrospective.^57^* | **OPMD (40)**  PVL (28)  OL (12) | 788 | NR | NR | NR | MiED (6) | **OPMD= 5.08**  PVL= 3.55  OL= 1.52 |
| Ghosh et al., 2017, India.  Cross-sectional. ¥^58^ | **OPMD (33)**  OSMF (21)  OL (12) | 77 | NR | NR | NR | OL  SED (5) | **OPMD= 42.85**  OSMF= 27.27  OL= 15.58 |
| Gómez et al., 2024, Cuba.  Cross-sectional.^59^ | **OPMD (47)**  OL (37)  OE (10) | 5259 | NR | 20-34 (3)  35-59 (7)  ≥60 (37) | NR | NR | **OPMD= 0.89**  OL = 0.70  OE= 0.19 |
| Goutzanis, 2022, Greece.  Cross-sectional. ¥^60^ | OL (27) | 497 | M (9) F (18) | Mean±SD  55.1±12.8 | NR | ED (19)  HP (5)  psHP (3) | OL= 5.43 |
| Goyal and Goyal, 2021, India. Cross-sectional. ¥^61^ | **OPMD (120)**  OSMF (81)  OL (27)  OE (12) | 14400 | NR | NR | NR | NR | **OPMD= 0.83**  OSMF= 0.56  OL= 0.19  OE = 0.08 |
| Gupta et al., 2022, India. Retrospective.^62^* | **OPMD (4)** | 73 | M (4)  F (0) | Mean  HP+MoEP (55)  PVL (67) |  | HP+MoEP (3)  PVL (1) | **OPMD= 5.48** |
| Gupta et al., 2023, Nepal.  Cross-sectional.^63^ | **OPMD (545)**  OL (353)  - HOL (325)  - NHOL (28)  OSMF (189)  OE (3) | 16572 | M (405)  F (140) | 11-20 (13)  21-30 (49)  31-40 (132)  41-50 (161)  51-60 (139)  >60 (51) | NR | NR | **OPMD= 3.29**  OL= 2.13  - HOL=1.96  - NHOL=0.17  OSMF= 1.14  OE= 0.02 |
| Hóstio et al., 2020, Brazil. Retrospective.^64^ | **OPMD (85)**  AC (59)  OL (23)  OE (3) | 631 | M (49)  F (36) | ≤29 (5)  30-39 (10)  40-49 (10)  50-59 (22)  60-69 (20)  70-79 (12)  ≥80 (3) | Alcohol past (4)  Non-alcohol past (81)  Alcohol present (27)  Non-alcohol present (58)  Ex-smoker (26)  Non—ex-smoker (59)  Smoking (15)  Non-smoker (70) | NR | **OPMD= 13.47**  AC= 9.35  OL= 3.65  OE= 0.48 |
| Iyer et al., 2023, India.  Cross-sectional. ¥^65^ | **OPMD (51)**  OL (38)  - NHOL (4)  - NI (34)  OSMF (13)  PVL (2) | 40852 | NR | NR | OL  Smoking (21) | MiED (21)  MoED (1)  OSMF (13) | **OPMD= 0.12**  OL= 0.09  OSMF= 0.03  PVL= 0.0049 |
| Kalantari and Samani. 2022, Iran. Retrospective.^66^ | **OPMD (56)**  OL (55)  - NHOL (55)  AC (1) | 2092 | M (30) F (26) | Mean±SD  (49.51±12.77)  Range (18-83) | NR | ED (55)  AC (1) | **OPMD= 2.67**  OL= 2.63  AC= 0.04 |
| Kalavathi et., 2023, India. Retrospective.^67^ | OL (4) | 105 | M (2) F (2) | Range (41-59)  Average (47) | NR | HP (3) MiED (1) | OL= 3.81 |
| Kamble et al., 2018, India. Prospective. ¥^68^ | **OPMD (45)**  OSMF (35)  OL (10) | 1500 | M (44)  F (1) | 17-24 (7)  25-34 (16)  35-44 (14)  ≥44 (8) | NR | NR | **OPMD= 3.00**  OSMF= 2.33  OL= 0.67 |
| Klongnoi et al., 2021, Thailand. ¥^69^ | **OPMD (230)** | 88201 | NR | NR | NR | MiED (134)  MoED (40)  SED (18)  HK (11)  HKA (6)  EA (4)  HP (4)  VHP (4)  HP+candidiasis (3)  AC (3)  OSMF (2)  HP/HK (1) | **OPMD= 0.26** |
| Korkmaz et al., 2020, Turkey.  ¥^70^ | **OPMD (20)**  OL (14)  OE (6) | 361 | NR | 45-54 (9)  55-64 (2)  65-74 (4)  >75 (5) | NR | NR | **OPMD= 5.54**  OL= 3.88  OE= 1.66 |
| Kumar et al., 2022, India.  Cross-sectional. ¥^71^ | **OPMD (35)**  OL (27)  OSMF (5)  AC (3) | 392 | NR | NR | NR | NR | **OPMD= 8.93**  OL= 6.89  OSMF= 1.28  AC= 0.77 |
| Kusiak et al., 2020, Poland. Retrospective.^72^ | OL (416) | 5720 | M (220)  F (196) | Average (45.6)  Range (21-86)  21-40 (73)  41-60 (194)  >60 (149) | Smokers (363) | NR | **OPMD= 7.27** |
| Kuzio et al., 2020, Poland. Retrospective.^73^ | OL (8) | 176 | NR | NR | NR | HP (6)  ED (2) | OL= 4.55 |
| Li et al., 2021, China. Retrospective. ¥^74^* | OL (913) | 6859 | NR | NR | NR | ED (477)  HK (436) | OL= 13.31 |
| Linares et al., 2023, Brazil.  Cross-sectional.^75^ | **OPMD (25)**  AC (13)  OL (12) | 756 | NR | NR | NR | NR | **OPMD= 3.31**  AC= 1.72  OL= 1.59 |
| Mala et al., 2024, India. Retrospective.^76^ | **OPMD (16)**  OSMF (10)  OL (6) | 220 | NR | NR | NR | NR | **OPMD= 7.27**  OSMF= 4.55  OL= 2.73 |
| Maleki et al., 2022, Iran.  Cross-sectional. ¥^77^ | OL (28) | 11964 | NR | NR | NR | NR | OL= 0.23 |
| Meenapriya and Rajendran, 2020, India. Retrospective. ¥^78^ | **OPMD (23)**  OSMF (13)  OL (7)  OL+OSMF (2)  AC (1) | 610 | M (11)  F (12) | 20-25 (2)  25-30 (2)  30-35 (2)  35-40 (1)  40-45 (2)  45-50 (2)  50-55 (6)  55-60 (4)  60-65 (1)  65-70 (1) | Both (4)  Smokeless (12)  Smoking (3)  None (23) | NR | **OPMD= 3.77**  OSMF= 2.13  OL= 1.15  OL+OSMF= 0.33  AC= 0.16 |
| Mello et al., 2018, Brazil. Retrospective. ¥^79^ | **OPMD (202)**  OL (193)  - NHOL (21)  - NI (172)  OE (9) | 2633 | NR | NR | NR | HKA (58)  MiED (85)  MoED (40)  SED (19) | **OPMD= 7.67**  OL= 7.33  - NHOL= 0.80  OE= 0.34 |
| Menon et al., 2025, India.  Cross-sectional. ^80^ | **OPMD (139)**  OL (61) OSMF (52)  OL+OSMF (26) | 624 | NR | NR | NR | NR | **OPMD= 22.28**  OL= 9.78 OSMF= 8.33  OL+OSMF= 4.17 |
| Modi et al., 2023, India. Retrospective. ¥^81^ | OL (17) | 259 | NR | NR | NR | NR | OL= 6.56 |
| Monteiro et al., 2017, Portugal. Retrospective. ¥^82^ | **OPMD (102)**  OL (83)  AC (18)  OE (1) | 3212 | OL  M (52) F (31)  AC  M (9) F (9)  OE  F (1) | Mean±SD  OL nED (57.53±15.66)  AC  (64.94±8/2)  OL ED (62.29±10.83)  OE (70±0) | NR | OL  ED (7)  HK/EHP (76) | **OPMD= 3.18**  OL= 2.58  AC= 0.56  OE= 0.03 |
| Nethan et al., 2021, India.  Cross-sectional. ¥^83^ | **OPMD (1902)**  OL (1388)  OL+OSMF (273)  OL+OE (183) OSMF (58) | 71022 | NR | NR | NR | NR | **OPMD= 2.68**  OL= 1.95  OL+OSMF= 0.38  OL+NHOL= 0.26 OSMF= 0.08 |
| Oliveira et al., 2018, Brazil. Cross-sectional. ^84^ | OL (59) | 925 | M (36)  F (23) | Mean±SD  57.8±9.8  10-29 (1)  30-49 (6)  50-69 (48)  70-89 (3) | Alcohol (13)  No alcohol (20)  Tobacco (39)  No Tobacco (3)  Both (8)  No combination (26) | HK (3)  HKA (5)  HK/HP (21)  HP (1)  EA/HP (2)  HP/HKA (1)  ED (26)  nED (33) | OL= 6.38 |
| Onofrei et al., 2024, Romania.^85^ | **OPMD (49)**  AC (27) OL (22) | 1610 | M (32)  F (17) | ≤50 (5)  >50 (44) | NR | NR | **OPMD= 3.04**  AC= 1.68  OL= 1.37 |
| Oreamuno et al., 2019, Costa Rica. Retrospective. ¥^86^ | **OPMD (27)**  OL (15)  AC (12) | 263 | NR | NR | NR | HK (9)  HK/MiED (5)  MiED (6)  MoED (3)  AC (4) | **OPMD= 10.27**  OL= 5.70  AC= 4.56 |
| Paiva e Costa, 2021, Brazil, Mexico and Argentina. Descriptive.^87^ | OL (476) | 114893 | M (227)  F (249) | Mean±SD  (60.27±14.84)  <20 (3)  20-30 (5)  30-40 (31)  40-50 (62)  50-60 (115)  >60 (238)  NR (22) | NR | NR | OL= 0.41 |
| Pandiar et al., 2023, India. Retrospective. ^88^ | OSMF (238) | 7098 | M (216) F (22) | OSMF  Mean±SD  40.94±12.35  Median (39)  Range (18-77)  <19 (2)  20-29 (48)  30-39 (71)  40-49 (58)  50-59 (37)  >60 (22) | Paan chewing (75)  Non-Paan chewing (111)  Paan+Smoking (28)  Non- Paan+Smoking (158)  Areca nut (11) | nED (181)  MiED (21)  MoED (21)  SED (15) | OSMF= 3.35 |
| Radwan-Oczko et al., 2022, Poland. Retrospective.^89^ | OL (176) | 2747 | M (65)  F (111) | Median (54.5) | NR | NR | OL= 6.41 |
| Reddy et al., 2018, USA. Retrospective. ¥^90^* | AC (913) | 2487 | NR | NR | NR | AC (913) | AC= 36.71 |
| Rodrigues et al., 2018, Brazil. Retrospective. ¥^91^ | **OPMD (133)**  AC (68)  OL (61)  OE (4) | 2706 | M (87) F (46) | 0-40 (52)  >40 (81) | Alcohol (43)  Non-alcohol (90)  Smokers (51)  Non-smokers (82)  Sun exposure (58)  Non-sun exposure (75) | SE (1)  SE-MiED (1)  HK (11)  MiED (5)  MoED (3)  Inconclusive (1) | **OPMD= 4.92**  AC= 2.51  OL= 2.25  OE= 0.15 |
| Rodríguez et al., 2019, Cuba. Cross-sectional.^92^ | **OPMD (21)**  OL (16)  OE (5) | 56 | NR | NR | NR | NR | **OPMD= 37.50**  OL= 28.57  OE= 8.93 |
| Saghravanian et al., 2017, Iran. Retrospective. ¥^93^ | **OPMD (117)**  OL (115)  OE (2) | 11126 | M (68)  F (47) | 20-29 (7)  30-39 (11)  40-49 (15)  50-59 (33)  60-69 (19)  70-79 (20)  80-89 (4) | NR | NR | **OPMD= 1.05**  OL= 1.03  OE= 0.02 |
| Sahoo et al., 2021, India. Retrospective. ¥^94^ | **OPMD (11)** | 692 | NR | NR | NR | MiED (7)  MoED (2)  SED (2) | **OPMD= 1.59** |
| Saiegh et al., 2017, Argentina. ¥^95^ | **OPMD (9)**  OL (6)  AC (3) | 503 | NR | NR | NR | NR | **OPMD= 1.79**  OL= 1.19  AC= 0.60 |
| Santos et al., 2024, Brazil. Retrospective.^96^ | **OPMD (571)**  OL (505)  AC (46)  OE+NHOL (20) | 32698 | M (316)  F (275)  NI (3) | <40 (89)  41-50 (143)  >51 (405)  NR (40) | Smoking  No (110)  Yes (190)  Former (65)  NR (302)  Alcohol  No (163)  Yes (123)  Former (29)  NR (352) | nED (446)  ED (125) | **OPMD= 1.75**  OL= 1.54  AC= 0.14  OE+NHOL= 0.06 |
| Shamloo et al., 2022, Iran. Retrospective.^97^* | AC (40) | 512 | NR | NR | NR | NR | AC= 7.81 |
| Shoorgashti et al., 2024, Iran. Observational.^98^ | OL (3) | 200 | NR | NR | Smoking  Yes (3) | NR | OL= 1.50 |
| Silva et al., 2019, Brazil. Retrospective. ¥^99^ | AC (3) | 106 | NR | NR | NR | ED (3) | AC= 2.83 |
| Silva et al., 2020, Brazil. Retrospective. ^100^* | AC (2017) | 198709 | M (1439) F (575)  NR (3) | 0-9 (3)  10-19 (7)  20-29 (57)  30-39 (142)  40-49 (276)  50-59 (475)  60-69 (570)  70-79 (309)  80-89 (67)  90-99 (5)  100-109 (1)  NR (104) | Alcohol (33)  Ex-consumer alcohol (3)  NR alcohol (1981)  Smokers (80)  Non-smokers (3)  Ex- consumer (23)  NR smokers (1911)  Sun exposure (75)  Non-sun exposure (7)  NR sun exposure (1935) | AC (2017) | AC= 1.02 |
| Silva et al., 2024, Brazil.  Cross-sectional.^101^ | **OPMD (34)**  OL (17)  AC (17) | 1388 | M (16)  F (18) | NR | NR | NR | **OPMD = 2.45**  OL= 1.22  AC= 1.22 |
| Vasconcelos et al., 2017, Brazil.^102^ | AC (39) | 1550 | NR | NR | NR | NR | AC= 2.52 |
| Venkat et al., 2022, India. Retrospective.^103^ | **OPMD (106)**  OL (85)  OSMF (21) | 2376 | OL  M (55)  F (30) | OL  21-40 (27)  41-60 (41)  >60 (17) | OL  Tobacco (68)  No habits (17) | HK (43)  MiED (43)  MoED (15)  SED (4) | **OPMD= 4.46**  OL= 3.58  OSMF= 0.88 |
| Venkatesh et al., 2024, India. Retrospective.^104^ | **OPMD (71)**  OL (47)  OSMF+OL (23)  PVL (1) | 2600 | M (61)  F (10) | OL  ≤30 (3)  31-40 (15)  41-50 (23)  51-60 (22)  >60 (6) | NR | nED (8)  MiED (17)  MoED (30)  SED (16) | **OPMD= 2.73**  OL= 1.81  OSMF+OL= 0.88  PVL = 0.04 |
| Verma and Sharma., 2019, India.  Cross-sectional.^105^ | **OPMD (115)**  OSMF (54)  OL (41)  OE (20) | 872 | NR | NR | Tobacco use:  <5 times (16)  5-10 times (31)  >10 times (68) | NR | **OPMD= 13.19**  OSMF= 6.19  OL= 4.70  OE= 2.29 |
| Villa et al., 2024, USA. Retrospective.^106^ | **OPMD (1224)**  OL (1124)  OSMF (78)  OE (22) | 4225251 | M (665)  F (558) | Median (IQR)  OL 61 (46-70)  OE 58 (52-69)  OSMF 51 (33-63) | Smoking  Never (588)  Ever (427)  Alcohol Misuse  No (991)  Yes (39) | NR | **OPMD= 0.029**  OL= 0.03  OSMF= 0.0018  OE= 0.0005 |
| Wongviriya et al., 2018, Thailand. ¥^107^ | OL (6)  - NHOL (2)  - NI (4) | 211 | M (1)  F (5) | 60-64 (1)  65-69 (3)  ≥70 (2) | Alcohol (1)  Non-alcohol (5)  Areca nut chewing (5)  Non-areca nut chewing (1)  Smoking (2)  Non-smoking (4) | NR | OL= 2.84  - NHOL=0.95 |
| Yen et al., 2018, Taiwan. Longitudinal.^108^ | **OPMD (1357)** | 235234 | M (1137)  F (220) | 50-54 (347)  55-59 (361)  60-64 (264)  65-69 (385) | Betel quid chewing (235)  Both (689)  Smoking (433) | NR | **OPMD= 0.58** |

**Legend**: **AC**, actinic cheilitis; **EA**, epithelial acanthosis; **ED**, epithelial dysplasia; **EHP**, epithelial hyperplasia; **F**, females; **HK**, hyperkeratosis; **HKA**, hyperkeratosis and acanthosis; **HOE**, homogeneous oral erythroplakia; **HOL**, homogeneous oral leukoplakia; **HOK**, hyperorthokeratosis; **HOPK**, hyperortho/parakeratosis; **HPK** hyperparakeratosis **HP**, hyperplasia; **IQR**, interquartile range; **LS**, lesions; **M**, males; **MiED**, mild epithelial dysplasia; **MoED**, moderate epithelial dysplasia; **nED**, non-dysplastic; **NHOL**, non-homogeneous leukoplakia; **NI**: cases in which leukoplakia subtype (homogeneous/non-homogeneous) was not specified; **NK**, non-keratinization; **NR**, not reported; **OE**, oral erythroplakia; **OL**, oral leukoplakia; **OPMD**, oral potentially malignant disorders; **OSMF**, oral submucous fibrosis; **psHP**, pseudoepitheliomatous hyperplasia; **SE**, solar elastosis; **SED**, severe epithelial dysplasia; **SD**, standard deviation; **USA**, United States of America; **PVL**, proliferative verrucous leucoplakia; **VHP**, verrucous hyperplasia. The type of study, when provided, was reported as stated by the authors of the included studies. **¥** Cases in which oral lichen planus, OSCC, or dysplasia were reported as clinical diagnoses—or classified as unspecified clinical diagnoses—were excluded. Clinicopathological data were extracted only when they could be analyzed independently for the selected sample. Erytroleukoplakia and leukoerythroplakia were described as NHOL. *****Studies excluded in the sensitivity meta-analyses were those restricted to predetermined anatomical sites (lip, palate, tongue, or gingiva).

**Supplemental References:**

1. Amarasinghe HK, Usgodaarachchi US, Johnson NW, et al. Betel-quid chewing with or without tobacco is a major risk factor for oral potentially malignant disorders in Sri Lanka: a case-control study. Oral Oncol. 2010;46:297-301. <https://doi.org/10.1016/j.oraloncology.2010.01.017>
2. Campisi G, Margiotta V. Oral mucosal lesions and risk habits among men in an Italian study population. J Oral Pathol Med. 2001;30:22-28. <https://doi.org/10.1034/j.1600-0714.2001.300104.x>
3. Cecotti EL, Yasnig F, Villamonte S, et al. Lesiones cancerizables en patología bucal. Bol Acad Nac Med BAires. 1997;75:537-544.
4. Femopase FL, Binagui MV, Blanc SL, et al. A comparative study of oral lichen planus and leukoplakia in two Argentine populations. Acta Odontol Latinoam. 1997;10:89-99.
5. Vallejo MJGP, Martın JMG, Garcıa MG, et al. Lesiones precancerosas (leucoplasia y liquen plano oral) en el paciente geriatrico. Aten Primaria. 1997; 20:41-44.
6. Haas Junior OL, Rosa FM da, Burzlaff JB, et al. Definição do grupo de risco para leucoplasias bucais: estudo retrospectivo entre os anos de 1999 e 2009. RFO UPF. 2011;16:261-266.
7. Idris A, Vani N, Saleh S, et al. Relative Frequency of Oral Malignancies and Oral Precancer in the Biopsy Service of Jazan Province, 2009-2014. Asian Pac J Cancer Prev. 2016;17:519-525. <https://doi.org/10.7314/APJCP.2016.17.2>
8. Jahanbani J. Prevalence of oral leukoplakia and lichen planus in 1167 Iranian textile workers. Oral Dis. 2003;9:302-304. <https://doi.org/10.1034/j.1601-0825.2003.00967.x>
9. Kaugars GE, Pillion T, Svirsky JA, et al. Actinic cheilitis: A review of 152 cases. Oral Surg Oral Med Oral Pathol Oral Radiol Endod. 1999;88:181-186. <https://doi.org/10.1016/S1079-2104(99)70115-0>
10. Lapthanasupkul P, Poomsawat S, Punyasingh J. A clinicopathologic study of oral leukoplakia and erythroplakia in a Thai population. Quintessence Int. 2007;38:e448-455.
11. Mendez M, Carrard VC, Haas AN, et al. A 10-year study of specimens submitted to oral pathology laboratory analysis: lesion occurrence and demographic features. Braz Oral Res. 2012;26:235-241. <https://doi.org/10.1590/S1806-83242012000300009>
12. Misra V, Singh P, Lal N, et al. Changing pattern of oral cavity lesions and personal habits over a decade: Hospital based record analysis from Allahabad. Indian J Community Med. 2009;34:321-325. <https://doi.org/10.4103/0970-0218.58391>
13. Ntomouchtsis A, Karakinaris G, Poulolpoulos A, et al. Benign lip lesions. A 10-year retrospective study. Oral Maxillofac Surg. 2010;14:115-118. <https://doi.org/10.1007/s10006-009-0196-y>
14. Pentenero M, Broccoletti R, Carbone M, et al. The prevalence of oral mucosal lesions in adults from the Turin area. Oral Dis. 2008;14:356-366. <https://doi.org/10.1111/j.1601-0825.2007.01391.x>
15. Queiroz SIML, Medeiros AMCD, Silva JSPD, et al. Clinical and histopathological evaluation and habits associated with the onset of oral leukoplakia and erythroplakia. Jornal Brasileiro de Patologia e Medicina Laboratorial. 2014;50:144-149. <https://doi.org/10.5935/1676-2444.20140008>
16. Sánchez JOB, Martínez LV, Dalacio DC, et al. Estudio histológico de lesiones de la cavidad bucal en el quinquenio 2001-2005. Arch Méd Camagüey. 2007;11:0-0.
17. Shafer WG, Waldron CA. Erythroplakia of the oral cavity. Cancer. 1975;36:1021-1028. [https://doi.org/10.1002/1097-0142(197509)36:3<1021::AID-CNCR2820360327>3.0.CO;2-W](https://doi.org/10.1002/1097-0142(197509)36:3%3c1021::AID-CNCR2820360327%3e3.0.CO;2-W)
18. Silveira ÉJDD, Lopes MFF, Silva LMM, et al. Lesões orais com potencial de malignização: análise clínica e morfológica de 205 casos. J Bras Patol Med Lab. 2009; 45:233-238. <https://doi.org/10.1590/S1676-24442009000300008>
19. Silverman S, Bhargava K, Mani NJ, et al. Malignant transformation and natural history of oral leukoplakia in 57,518 industrial workers of gujarat, india. Cancer. 1976;38:1790-1795. [https://doi.org/10.1002/1097-0142(197610)38:4<1790::AID-CNCR2820380456>3.0.CO;2-I](https://doi.org/10.1002/1097-0142(197610)38:4%3c1790::AID-CNCR2820380456%3e3.0.CO;2-I)
20. Souza LS de. Análise das patologias bucais e maxilofaciais de uma população brasileira durante um período de 15 anos. 2024. Accessed March 5, 2025. <https://repositorio.uel.br/handle/123456789/15078>
21. Starzyńska A, Pawłowska A, Renkielska D, et al. Oral premalignant lesions: epidemiological and clinical analysis in the northern Polish population. Postepy Dermatol Alergol. 2014;31:341-350. <https://doi.org/10.5114/pdia.2014.40932>
22. Yang YH, Ho PS, Lu HM, et al. Comparing dose-response measurements of oral habits on oral leukoplakia and oral submucous fibrosis from a community screening program. J Oral Pathol Med. 2010;39:306-312. <https://doi.org/10.1111/j.1600-0714.2009.00820.x>
23. Casnati B, Álvarez R, Massa F, et al. Prevalencia y factores de riesgo de las lesiones de la mucosa oral en la población urbana del Uruguay. Odontoestomatología. 2013;15:58-67.
24. Daftary DK, Pitkar VK, Gupta PC, et al. A study of the natural history of oral preleukoplakia. Acta Odontol Scand. 1978;36:327-331. <https://doi.org/10.3109/00016357809029083>
25. Elango KJ, Anandkrishnan N, Suresh A, et al. Mouth self-examination to improve oral cancer awareness and early detection in a high-risk population. Oral Oncol. 2011;47:620-624. <https://doi.org/10.1016/j.oraloncology.2011.05.001>
26. Ferreira A, Lucena EES, De Oliveira TC, et al. Prevalence and factors associated with oral potentially malignant disorders in Brazil’s rural workers. Oral Dis. 2016;22:536-542. <https://doi.org/10.1111/odi.12488>
27. Mehrotra R, Pandya S, Chaudhary AK, et al. Prevalence of Oral Pre-malignant and Malignant Lesions at a Tertiary Level Hospital in Allahabad, India. Asian Pacific Journal of Cancer Prevention. 2008;9:263-265.
28. Moret Y, Rivera H, Cartaya M. Prevalencia de lesiones en la mucosa bucal de pacientes diagnosticados en el Laboratorio Central de Histopatología bucal Dr. Pedro Tinoco de la Facultad de Odontología de la Universidad Central de Venezuela durante el período 1968-1987. Resultados Preliminares. Acta Odontol Venez. 2007;45:240-243.
29. Moret Y, Rivera H, González JM. Correlación clínico - patológica de lesiones diagnosticadas inicialmente como leucoplasia bucal y el diagnóstico histopatológico de displasia epitelial en una muestra de 11.250 pacientes adultos: Facultad de odontología. U.C.V. Acta Odontológica Venezolana. 2008;46:265-268.
30. Moret de González YN. Prevalencia de lesiones benignas y desórdenes potencialmente malignos que afectan la mucosa bucal en pacientes adultos Facultad de Odontoloía UCV. Acta Odontologica Venez. 2014;52.
31. Pindborg JJ, Mehta FS, Gupta PC, et al. Reverse smoking in andhra pradesh, india: a study of palatal lesions among 10,169 villagers. Br J Cancer. 1971;25:10-20. <https://doi.org/10.1038/bjc.1971.2>
32. Tang J, Jian X, Gao M, et al. Epidemiological survey of oral submucous fibrosis in Xiangtan City, Hunan Province, China. Comm Dent Oral Epidemiol. 1997;25:177-180. <https://doi.org/10.1111/j.1600-0528.1997.tb00918.x>
33. Waldron CA, Shafer WG. Leukoplakia revisited.A clinicopathologic study 3256 oral leukoplakias. Cancer. 1975;36:1386-1392. [https://doi.org/10.1002/1097-0142(197510)36:4<1386::AID-CNCR2820360430>3.0.CO;2-7](https://doi.org/10.1002/1097-0142(197510)36:4%3c1386::AID-CNCR2820360430%3e3.0.CO;2-7)
34. Agrawal S, Deshmukh P, Takalkar S, et al. Prevalence of oral sub mucosal fibrosis in pre- anesthesia clinic in india: a cross sectional observational study. J Pharm Research Int. 2021;33:218-222. <https://doi.org/10.9734/jpri/2021/v33i38A32077>
35. Ahern J, Toner M, O’ Regan E, et al. The spectrum of histological findings in oral biopsies. Ir Med J. 2019;112:1017.
36. AlOsaimi MM, AlAmri N, Wahass T, et al. Detection of oral mucosal lesions in tobacco and non-tobacco use patients: a case-control study. Int J Medicine Dev Ctries. 2023;7:1931-1936. <https://doi.org/10.24911/IJMDC.51-1698093851>
37. Anwar S, Tyagi N, Mathias YL, et al. A five year retrospective study of oral potentially malignant disorders (Opmds) and oral squamous cell carcinoma (Oscc) and their associated risk factors. J Datta Meghe Inst Med Scie Univ. 2023;18:460-467. <https://doi.org/10.4103/jdmimsu.jdmimsu_94_23>
38. Armelin AML, Hernandes ACP, Tomo S, et al. Oral lesions detected during a population screening for prevention and early diagnosis of oral cancer and potentially malignant disorders. Rev da Fac de Odontologia. 2019;24:350-354. <https://doi.org/10.5335/rfo.v24i3.9818>
39. Azevedo ABD, Dos Santos TCRB, Lopes MA, et al. Oral leukoplakia, leukoerythroplakia, erythroplakia and actinic cheilitis: Analysis of 953 patients focusing on oral epithelial dysplasia. J Oral Pathol Med. 2021;50:829-840. <https://doi.org/10.1111/jop.13183>
40. Barros CC, Medeiros CK, Rolim LS, et al. A retrospective 11-year study on lip lesions attended at an oral diagnostic service. Med Oral Patol Oral Cir Bucal. 2020;25:e370-e374. <https://doi.org/10.4317/medoral.23390>
41. Bastakoti S, Shrestha G, Gautam DK, et al. Clinico-pathological spectrum of oral cavity lesions at a tertiary care center in central nepal: a descriptive cross-sectional study. J Nepal Med Assoc. 2021;59:124-127. <https://doi.org/10.31729/jnma.5539>
42. Bhattacharjee T, Mukherjee K, Dash KC, et al. Habit-induced oral lesions in different occupations: A comparative study among people between geographical different places of West Bengal. J Family Med Prim Care. 2025;14:218-225. <https://doi.org/10.4103/jfmpc.jfmpc_1051_24>
43. Błochowiak K, Farynowska J, Sokalski J, et al. Benign tumours and tumour-like lesions in the oral cavity: a retrospective analysis. Advances in Dermatology and Allergolog. 2019;36:744-751. <https://doi.org/10.5114/ada.2018.78805>
44. Cesar ALM, Werneck JT, Picciani BLS, et al. Prevalência das lesões orais em idosos atendidos no serviço de estomatologia do Instituto de Saúde de Nova Friburgo ­UFF/RJ. Rev Cient CRO-RJ. 2021;6:57-62.
45. Cigic L, Martinovic D, Martinic J, et al. Increased prevalence of oral potentially malignant lesions among Croatian War invalids, a cross-sectional study. J Clin Exp Dent. 2023;15:e734-e741. <https://doi.org/10.4317/jced.60715>
46. Chiu SF, Ho CH, Chen YC, et al. Malignant transformation of oral potentially malignant disorders in Taiwan: An observational nationwide population database study. Medicine. 2021;100;e24934. <https://doi.org/10.1097/md.0000000000024934>
47. Collins J, Brache M, Ogando G, et al. Prevalence of oral mucosal lesions in an adult population from eight communities in Santo Domingo, Dominican Republic. Acta Odontol Latinoam. 2021;34:249-256. <https://doi.org/10.54589/aol.34/3/249>
48. Cordero-T K, Torres-M C, Anabalón-T P, et al. Screening de lesiones orales malignas y potencialmente malignas en funcionarios de universidad de valparaíso y universidad viña del mar durante los años 2016 - 2017. Int J Odontostomat. 2020;14:172-176. <https://doi.org/10.4067/S0718-381X2020000200172>
49. Cunha JLS, Cavalcante IL, Rodrigues ABR, et al. A retrospective multicenter study of oral and maxillofacial lesions in older people. Braz Oral Res. 2023;37:e098. <https://doi.org/10.1590/1807-3107bor-2023.vol37.0098>
50. Cury PR, Carvalho MJF, Araujo NS, et al. Occupational Exposure to Domestic Waste and oral Mucosal Lesions: a Cross-Sectional Study. J Health Sci. 2024;26:17-21. <https://doi.org/10.17921/2447-8938.2024v26n1p17-21>
51. Arruda EDS, Sombra GAD, Pereira JV, et al. Epidemiological survey of oral lesions diagnosed at a stomatology service. Rev Estomatol Herediana. 2021;31:156-162. <https://doi.org/10.20453/reh.v31i3.4044>
52. Dabla U, Ramalingam K, Tanwar M, et al. Clinico-pathological evaluation of gingival biopsies among Rajasthani population - An 4-year cross-sectional retrospective study in a single academic dental center. Journal of Pharmaceutical Negative Results. 2022;13:239-263.
53. De Almeida Cg, Freitas F, Francisco H, et al. Oral biopsies in a Portuguese population: A 20-year clinicopathological study in a university clinic. J Clin Exp Dent. 2022;14:e1024-e1031. <https://doi.org/10.4317/jced.59688>
54. De Souza Ml, Farias DM, Moura AEO, et al. A survey of lip lesions diagnosed in a single institution: A clinicopathological study. J Clin Exp Dent. 2024;16:e1517-e1522. <https://doi.org/10.4317/jced.62203>
55. Dhanuthai K, Kintarak S, Subarnbhesaj A, et al. A multicenter study of tongue lesions from thailand. Eur J Dent. 2020;14:435-439. <https://doi.org/10.1055/s-0040-1713296>
56. Ferreira IDBP, Werneck JT, Barki MCDLJM, et al. Demographic and stomatological profiles of pacientes at the Instituto de Saúde de Nova Friburgo. RSBO. 2024;21:15-22. <https://doi.org/10.21726/rsbo.v21i1.2306>
57. Gambino A, Carbone M, Broccoletti R, et al. A report on the clinical-pathological correlations of 788 gingival lesion. Med Oral Patol Oral Cir Bucal. 2017;22:e686-e693. <https://doi.org/10.4317/medoral.21845>
58. Ghosh S, Pal S, Ghatak S, et al. A Clinicopathologic and Epidemiologic Study of Chronic White Lesions in the Oral Mucosa. Ear Nose Throat J. 2017;96:13-17. <https://doi.org/10.1177/014556131709600804>
59. Gómez YAG, Silva JW, González ML, et al. Caracterización de lesiones orales premalignas diagnosticadas en un servicio de Estomatología en Pinar del Río. MediSur. 2024;22:94-101.
60. Goutzanis L. Differential retrospective analysis in oral cancerous, pre-cancerous, and benign tissue biopsies. Cureus. 2022;14:e24956. <https://doi.org/10.7759/cureus.24956>
61. Goyal R, Goyal MK. Influence of life style factors on oral potentially malignant and malignant disorders: a cross sectional study. Indian J Otolaryngol Head Neck Surg. 2021;73:443-446. <https://doi.org/10.1007/s12070-020-02084-5>
62. Gupta V, Kaur H, Mishra D, et al. Distribution of biopsied gingival lesions according to the proceedings from the 2017 World Workshop classification: A three-year retrospective study. Dent Med Probl. 2022;59:105-110. <https://doi.org/10.17219/dmp/141555>
63. Gupta A, Shrestha P, Poudyal S, et al. Prevalence and distribution of oral mucosal lesions and normal variants among nepalese population. BioMed Res Int. 2023;2023:9375084. <https://doi.org/10.1155/2023/9375084>
64. Hóstio BM, Bernardino ÍDM, Pereira JV, et al. Oral cancer and potentially malignant disorders: a 12-year epidemiological and clinical analysis in a metropolitan region of northeastern Brazil. J Public Health. 2020;28:131-138. <https://doi.org/10.1007/s10389-019-01054-8>
65. Iyer K, Kumar M, Kannan R, et al. Clinical and histopathological correlation of oral malignancy and potentially malignant disorders based on a screening program at high-risk population in Tamil Nadu, India. Front Oral Health. 2023;4:1286780. <https://doi.org/10.3389/froh.2023.1286780>
66. Kalantari M, Samani AA. A survey of oral and maxillofacial biopsies over a 23-year period in the southeast of iran. Journal of Dentistry. 2022;23:298-306. <https://doi.org/10.30476/dentjods.2021.90355.1487>
67. Kalavathi LC, Chaitanya KV, Venkata VB. A bird’s-eye view of pathologist over diagnostic confusion of oral cavity lesions. Journal of Oral and Maxillofacial Pathology. 2023;27:266-274. <https://doi.org/10.4103/jomfp.jomfp_312_22>
68. Kamble K, Guddad S, Nayak A, et al. Prevalence of oral mucosal lesions in western maharashtra: a prospective study. J Indian Acad Oral Med Radiol. 2017;29:282-287. <https://doi.org/10.4103/jiaomr.JIAOMR_14_17>
69. Klongnoi B, Sresumatchai V, Khovidhunkit SOP, et al. Pilot model for community based oral cancer screening program: outcome from 4 northeastern provinces in thailand. Int J Environ Res Public Health. 2021;18:9390. <https://doi.org/10.3390/ijerph18179390>
70. Korkmaz MO, Dikicier BS, İlhan N, et al. Dermographic properties and correlation of oral mucosa lesions with dermatological preliminary diagnosis. ENT Updates. 2020;10:409-417. <https://doi.org/10.32448/entupdates.825640>
71. Kumar A, Srivastava D, Singh A, et al. A cross-sectional study on clinico pathological evaluation of oral cavity lesions. European Journal of Molecular & Clinical Medicine. 2022;9:810-816.
72. Kusiak A, Maj A, Cichońska D, et al. The analysis of the frequency of leukoplakia in reference of tobacco smoking among northern polish population. Int J Environ Res Public Health. 2020;17:6919. <https://doi.org/10.3390/ijerph17186919>
73. Kuzio M, Wapniarska K, Danilewicz M, et al. Oral epithelial dysplasia and oral cancer prevalence in routine white lesion biopsies – a 6-year retrospective study. J Pre Clin Clin Res. 2020;14:63-68. <https://doi.org/10.26444/jpccr/125393>
74. Li X, Zhang J, Zhang H, Li T. Biopsied non-dental plaque-induced gingival diseases in a Chinese population: a single-institute retrospective study. BMC Oral Health. 2021;21:265. <https://doi.org/10.1186/s12903-021-01614-z>
75. Linares MF, Lopes SMP, Moreira AEB, et al. Active search screening for oral potentially malignant disorders and oral cancer in the city of Piracicaba. Braz Oral Res. 2023;37:e015. <https://doi.org/10.1590/1807-3107bor-2023.vol37.0015>
76. Mala MK, Danda M, Rao PSN, et al. Exploring The Association Of Risk Variables To The Clinicopathological Characteristics Of Oral Lesions – A Retrospective Study. Int J Life Sci, Biotechnol Pharm Res. 2024;13:439-443. <https://doi.org/10.69605/ijlbpr_13.9.2024.77>
77. Maleki L, Khalesi A, Zahedi S. A 30-year retrospective epidemiological study of oral dysplastic lesions in an iranian population. Winter And Spring 2022;34:9-13.
78. Meenapriya M, Rajendran D. Prevalence of oral potentially malignant disorders among adult population: A retrospective study. Int J Pharm Sci Res. 2020;11:671-679. <https://doi.org/10.26452/ijrps.v11iSPL4.4016>
79. Mello FW, Melo G, Meurer MI, et al. Intraoral potentially malignant disorders in a brazilian oral pathology service: epidemiological, clinical, and histopathological findings. J Oncol. 2018;2018:2325808. <https://doi.org/10.1155/2018/2325808>
80. Menon PA, Sahana S, Mhaske S, et al. Prevalence and predictive risk factor analysis of potentially malignant oral mucosal lesions among gond tribes of Bhopal. J Oral Res Rev. 2025;17:9-14. <https://doi.org/10.4103/jorr.jorr_48_24>
81. Modi V, Kulhari S, Singh A, et al. Histopathological Spectrum of Oral Cavity Lesions at a Tertiary Care Center in Western Rajasthan. Int J Toxicol Pharmacol. Res. 2023;14:134-141.
82. Monteiro LS, Albuquerque R, Paiva A, et al. A comparative analysis of oral and maxillofacial pathology over a 16-year period, in the north of Portugal. International Dental Journal. 2017;67:38-45. <https://doi.org/10.1111/idj.12258>
83. Nethan ST, Lakshmi K, Ralhan R, et al. Assessment of the prevalence and relationship of tobacco use and associated oral lesions in an urban population of new delhi: a cross-sectional study. Indian J Surg. 2021;84:720-728. <https://doi.org/10.1007/s12262-021-03055-y>
84. Oliveira GC, Neves M, Moure SP. Prevalência e correlação clínico-patológica dos casos de leucoplasia bucal diagnosticados no Laboratório de Histologia da ULBRA, Canoas/RS. Stomatos. 2018;24:28-40.
85. Onofrei BA, Popa C, Sciuca AM, et al. Potentially malignant lesions in the oral cavity: a retrospective analysis. 2024. Rom J of Oral Rehabil. 2024; 16:326-333. <https://doi.org/10.62610/RJOR.2024.2.16.28>
86. Oreamuno YB, Soto AL. Retrospective analysis of oral mucosal lesions between 2008-2015 in the clinical boarding school of dentistry of the University of Costa Rica. Poblac Salud Mesoam. 2019;16:134-154. <https://doi.org/10.15517/psm.v0i0.34404>
87. Costa Am, Pontes Fs, Souza Ll, et al. What is the frequency of floor of the mouth lesions? A descritive study of 4,016 cases. Med Oral Patol Oral Cir Bucal. 2021;26:e738-e747. <https://doi.org/10.4317/medoral.24537>
88. Pandiar D, G P, Poothakulath Krishnan R, et al. Correlation between clinical and histopathological stagings of oral submucous fibrosis: a clinicopathological cognizance of 238 cases from south india. Cureus. 2023;15:e49107. <https://doi.org/10.7759/cureus.49107>
89. Radwan-Oczko M, Sokół I, Babuśka K, et al. Prevalence and characteristic of oral mucosa lesions. Symmetry. 2022;14:307. <https://doi.org/10.3390/sym14020307>
90. Reddy R, Davidova L, Bhattacharyya I, et al. Dermatologic lesions submitted to an oral and maxillofacial pathology biopsy service: an analysis of 2487 cases. Head Neck Pathol. 2018;12:493-499. <https://doi.org/10.1007/s12105-018-0885-7>
91. Rodrigues KS, Figueiredo VSAD, Abrantes Filho GND. Desordens orais potencialmente malignas: um estudo de prevalência. Rev Cir Traumatol Buco-Maxilo-Fac. 2018;18:6-16.
92. Rodríguez JL, Roldán SON, Rodríguez RGG. Caracterización clinicoepidemiológica de pacientes con cáncer bucal y otras lesiones del complejo bucomaxilofacial. MEDISAN 2019;23:837-846.
93. Saghravanian N, Mohtasham N, Ivani F, et al. The epidemiological pattern of premalignant and malignant epithelial lesions in northeast of iran: a 43-year evaluation. Int J Cancer Manag. 2017;10:e5403. <https://doi.org/10.5812/ijcm.5403>
94. Sahoo PK, Sarkar S, Ghosh D, et al. Premalignant and malignant lesions of oral cavity in eastern India: a hospital-based study. Eur J Cancer Prev. 2021;30:393-399. <https://doi.org/10.1097/cej.0000000000000640>
95. Saiegh J, Giacco C. CA, Adler IL, et al. Frecuencia de lesiones de la mucosa oral en el servicio de urgencias y orientación de pacientes de la facultad de odontología de la Universidad de Buenos Aires. Rev Fac Odontol (B Aires). 2017;32:5-10.
96. Santos NSD, Rodrigues AZ, Ferri CA, et al. Temporal epidemiological profile of oral potentially malignant disorders in southern Brazil. Braz Oral Res. 2024;38:e132. <https://doi.org/10.1590/1807-3107bor-2024.vol38.0132>
97. Shamloo N, Ghannadan A, Safarpour R. Epidemiological study of lip cancer between 2004 and 2016 in public hospitals of Tehran, Iran: Squamous cell carcinoma as the most common cancer. J of Oral Health and Oral Epidemiol. 2022;11:202-206. <https://doi.org/10.34172/johoe.2022.12>
98. Shoorgashti R, Moshiri A, Lesan S. Evaluation of Oral Mucosal Lesions in Iranian Smokers and Non-smokers. Niger J Clin Pract. 2024;27:467-474. <https://doi.org/10.4103/njcp.njcp_702_23>
99. Silva TCG da, Gonnelli FAS, Rocha LA, et al. Estudo epidemiológico de biópsias realizadas em uma clínica odontológica universitária no período entre 2011 e 2018. Rev Odontol Araçatuba. 2019;40:52-55.
100. Silva LVDO, De Arruda JAA, Abreu LG, et al. Demographic and clinicopathologic features of actinic cheilitis and lip squamous cell carcinoma: a brazilian multicentre study. Head Neck Pathol. 2020;14:899-908. <https://doi.org/10.1007/s12105-020-01142-2>
101. Silva BM, Ferreira PHG, Campos FSR, et al. Stomatological profile of elderly patients who received dental care at a reference Hospital of Belo Horizonte, Minas Gerais – Brazil: a cross-sectional study. Arq Odontol. 2024;60:93-101. <https://doi.org/10.35699/2178-1990.2024.51264>
102. Vasconcelos AC, Aburad C, Lima IFP, et al. A scientific survey on 1550 cases of oral lesions diagnosed in a Brazilian referral center. An Acad Bras Ciênc 2017;89:1691-1697. <https://doi.org/10.1590/0001-3765201720170006>
103. Venkat A, Kumar MS, Aravindhan R, et al. Analysis of oral leukoplakia and tobacco-related habits in population of chengalpattu district- an institution-based retrospective study. Cureus. 2022;14: e25936. <https://doi.org/10.7759/cureus.25936>
104. Venkatesh DP, Ramalingam K, Ramani P, et al. Epidemiological Trends and Clinicopathological Characteristics of Oral Leukoplakia: A Retrospective Analysis From a Single Institution in Chennai, Tamil Nadu, India. Cureus. 2024;16:e61590. <https://doi.org/10.7759/cureus.61590>
105. Verma S, Sharma H. Prevalence of Oral mucosal lesions and their association with Pattern of tobacco use among patients visiting a dental institution. Indian J Dent Res. 2019;30:652-655. <https://doi.org/10.4103/ijdr.ijdr_23_18>
106. Villa A, Lodolo M, Ha P. Oncological Outcomes of Patients With Oral Potentially Malignant Disorders. JAMA Otolaryngol Head Neck Surg. 2024;151:65-71. <https://doi.org/10.1001/jamaoto.2024.3719>
107. Wongviriya A, Samnieng P, Intapa C, et al. Oral Mucosal Lesions in Thai Elderly Dental Patients. J Med Assoc Thai. 2018;101:367-373.
108. Yen AM, Wang S, Feng S, et al. The association between fecal hemoglobin concentration and oral potentially malignant disorders. Oral Dis. 2019;25:108-116. <https://doi.org/10.1111/odi.12978>
