# Supplementary figures and images for: Global Prevalence of Oral Potentially Malignant Disorders: An Updated Systematic Review and Meta‐Analysis
Source: J Oral Pathol Med. 2026 Apr 28;55(7):747–54. doi: 10.1111/jop.70146 (PMC13429371; doi:10.1111/jop.70146)

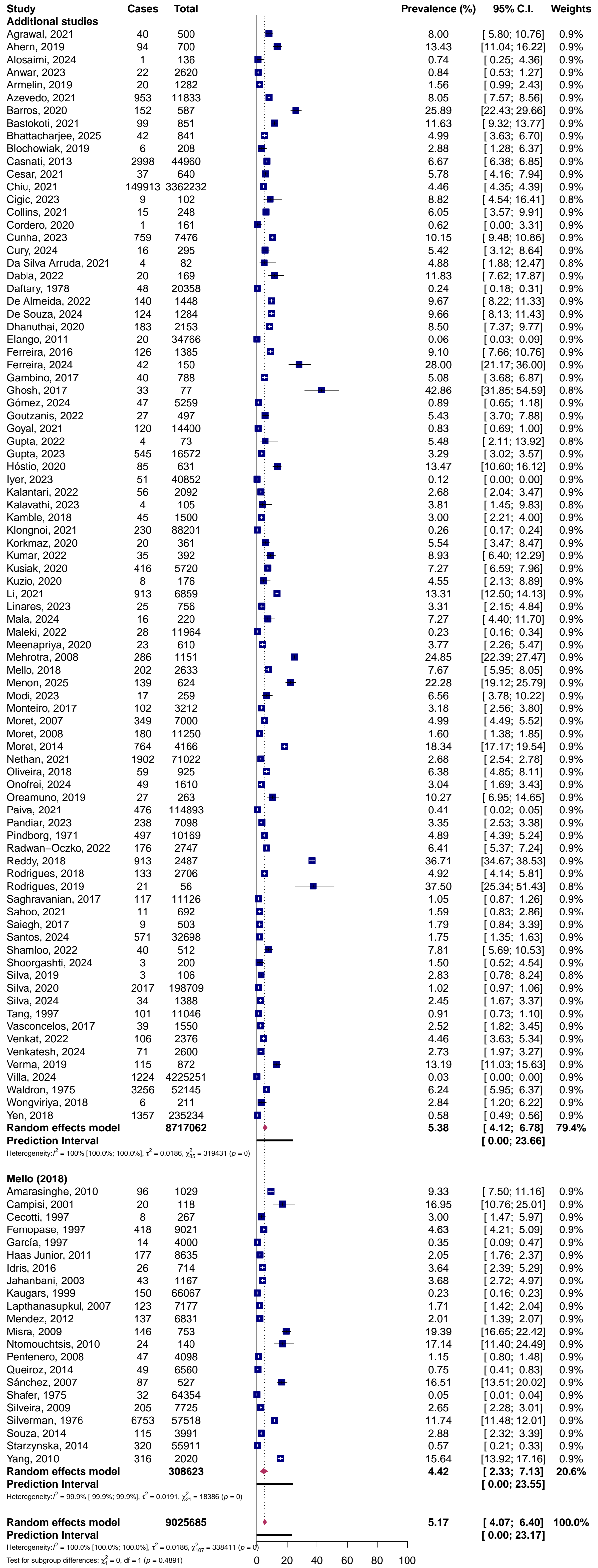

Supplement: Supplementary file 5 — Appendix S5: Meta‐analysis of the global prevalence of oral potentially malignant disorders. [file JOP-55-747-s020.pdf]

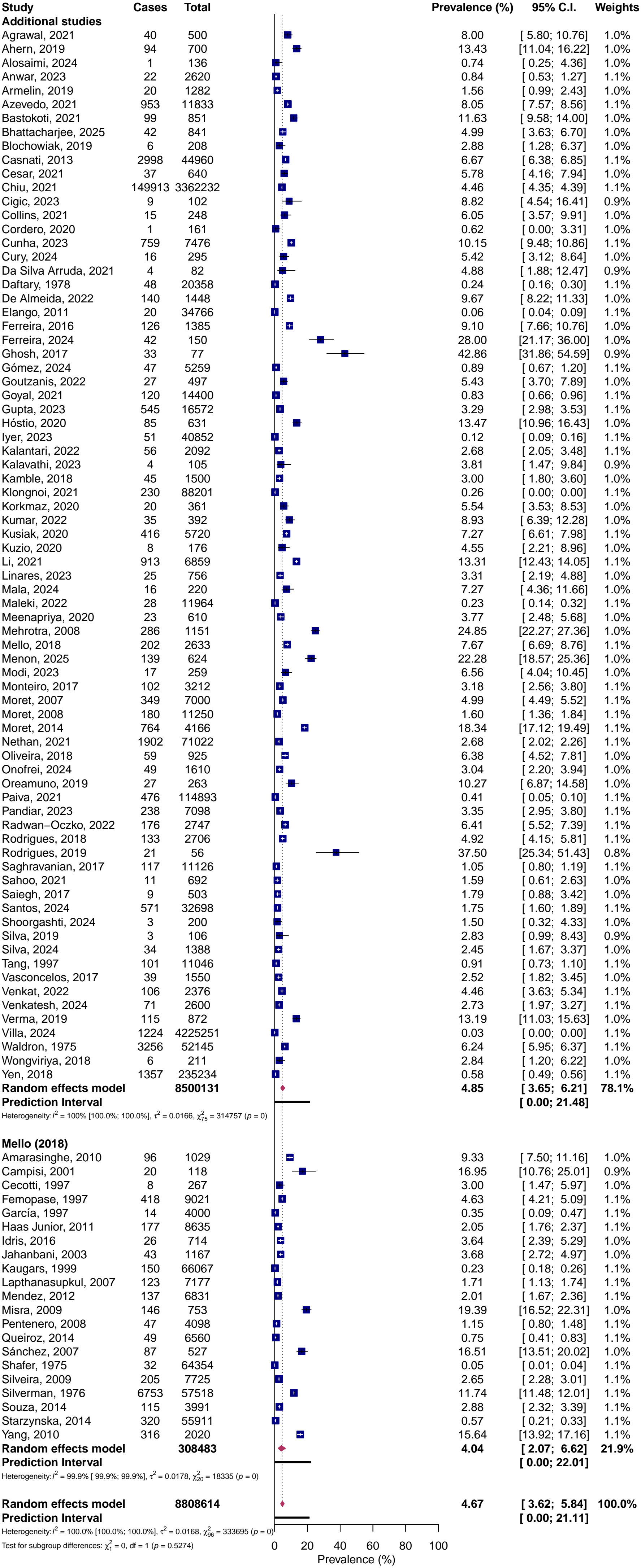

Supplement: Supplementary file 6 — Appendix S6: Sensitivity meta‐analysis of the global prevalence of oral potentially malignant disorders. [file JOP-55-747-s015.pdf]

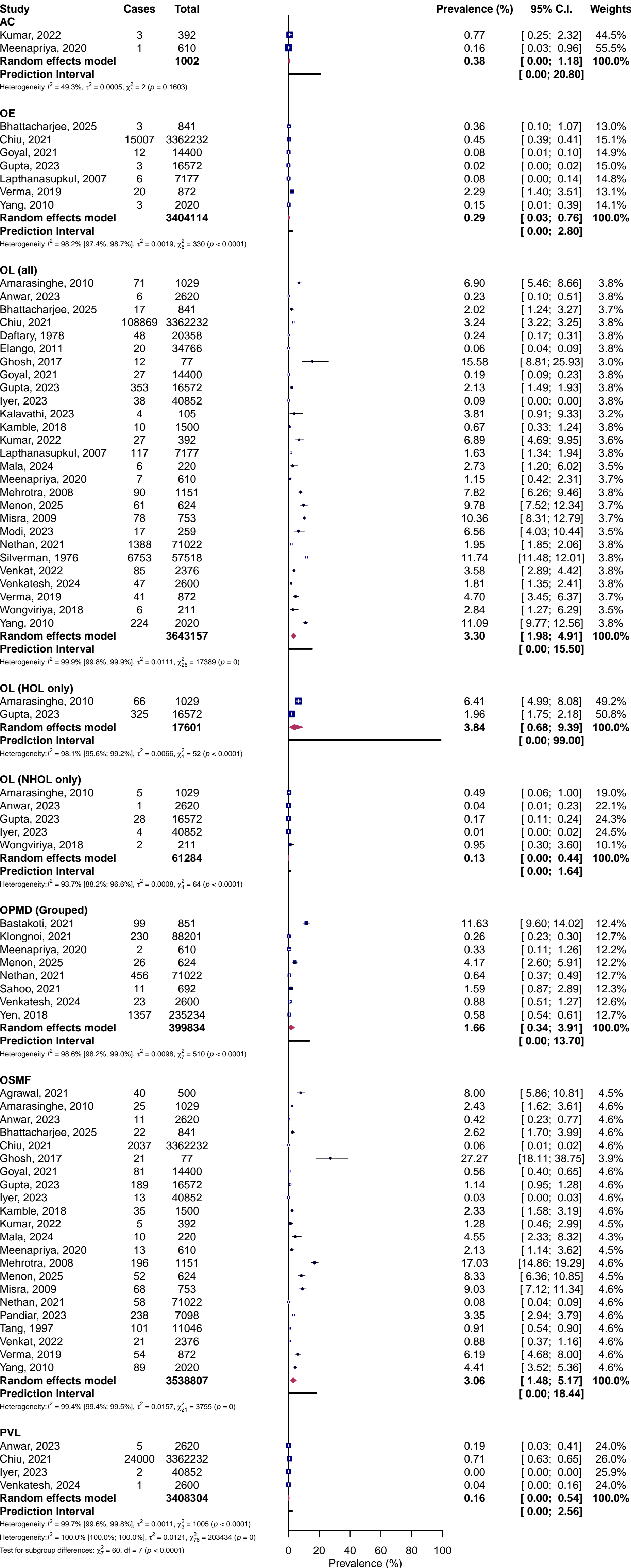

Supplement: Supplementary file 7 — Appendix S7: Sensitivity meta‐analysis of pooled prevalence by diagnosis in Asia. [file JOP-55-747-s004.pdf]

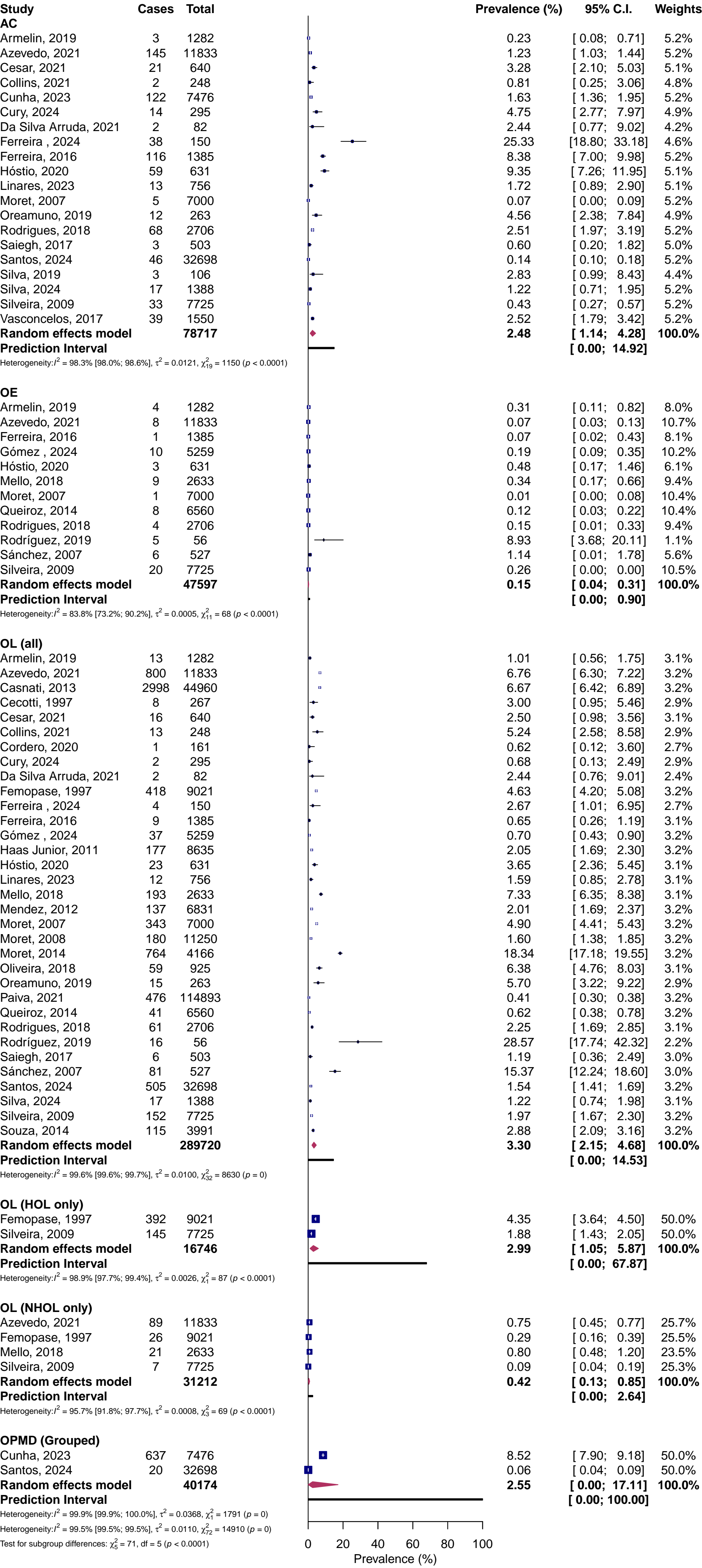

Supplement: Supplementary file 8 — Appendix S8: Sensitivity meta‐analysis of pooled prevalence by diagnosis in South America and the Caribbean. [file JOP-55-747-s002.pdf]

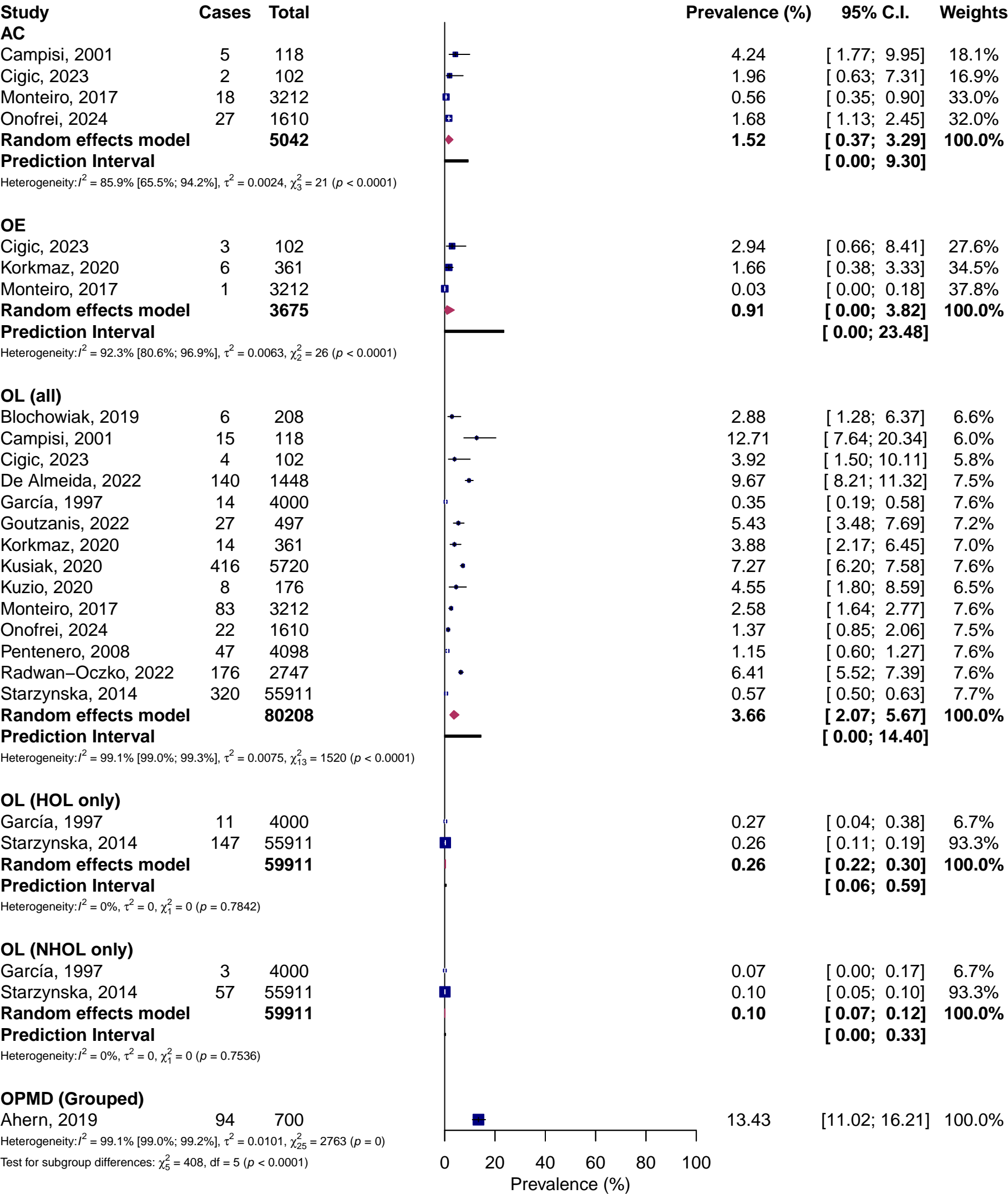

Supplement: Supplementary file 9 — Appendix S9: Sensitivity meta‐analysis of pooled prevalence by diagnosis in Europe. [file JOP-55-747-s016.pdf]

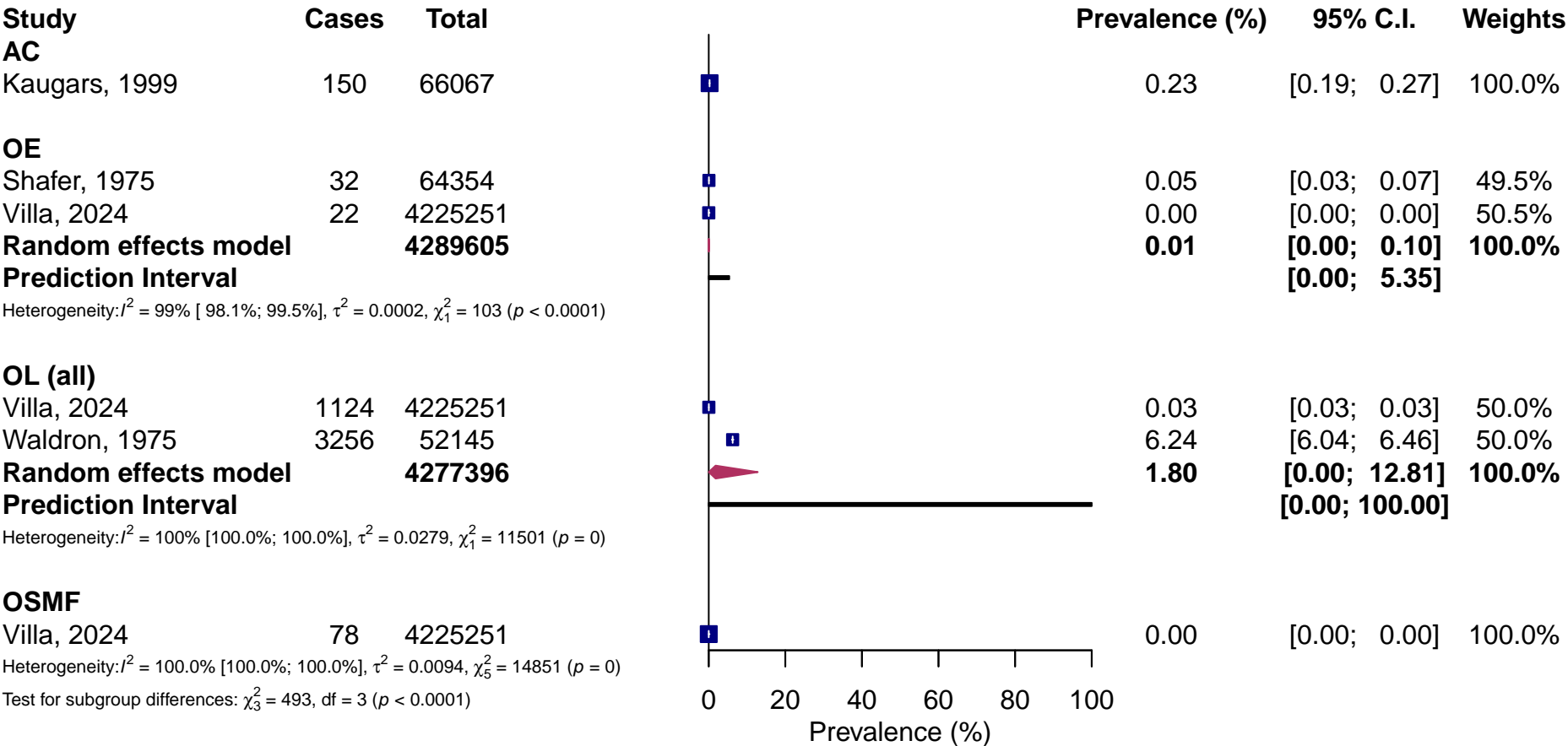

Supplement: Supplementary file 10 — Appendix S10: Sensitivity meta‐analysis of pooled prevalence by diagnosis in North America. [file JOP-55-747-s007.pdf]

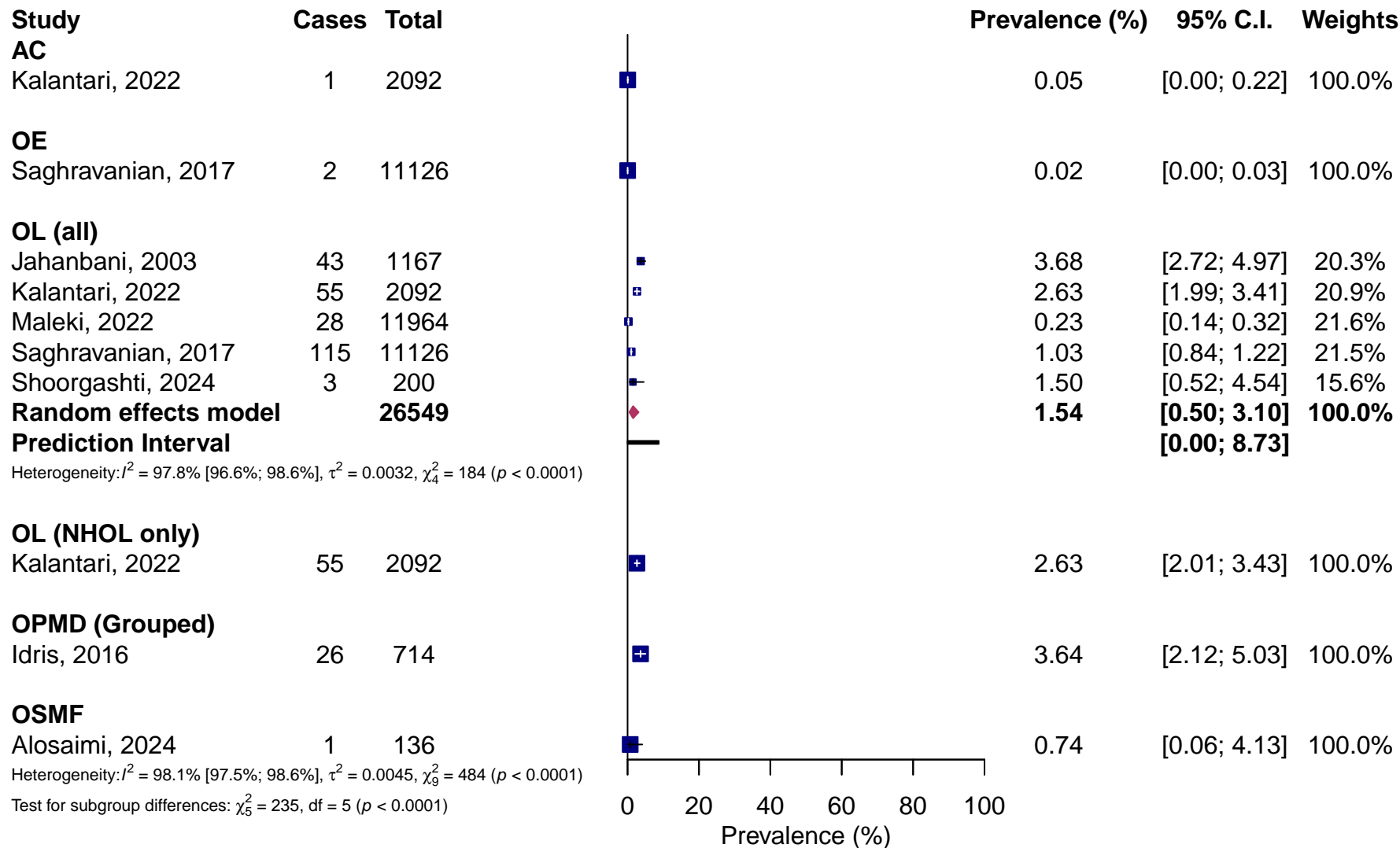

Supplement: Supplementary file 11 — Appendix S11: Sensitivity meta‐analysis of pooled prevalence by diagnosis in the Middle East. [file JOP-55-747-s017.pdf]

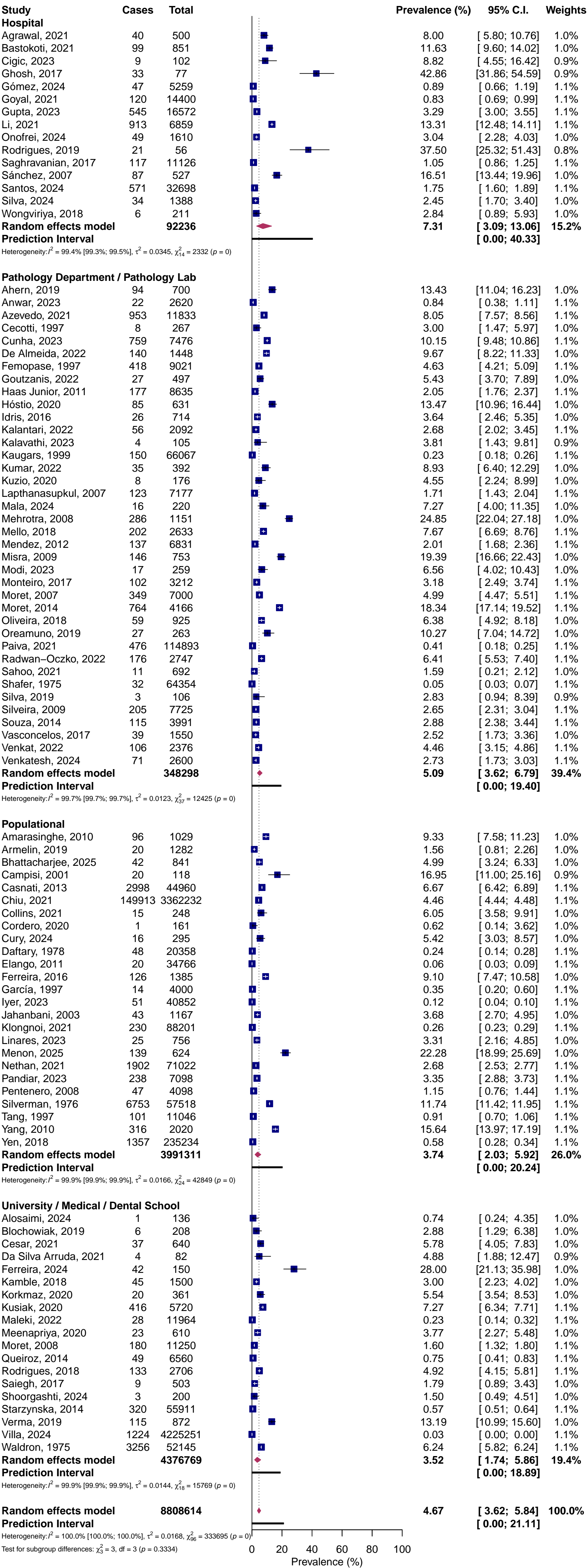

Supplement: Supplementary file 13 — Appendix S13: Sensitivity meta‐analysis of pooled prevalence by sample source. [file JOP-55-747-s006.pdf]

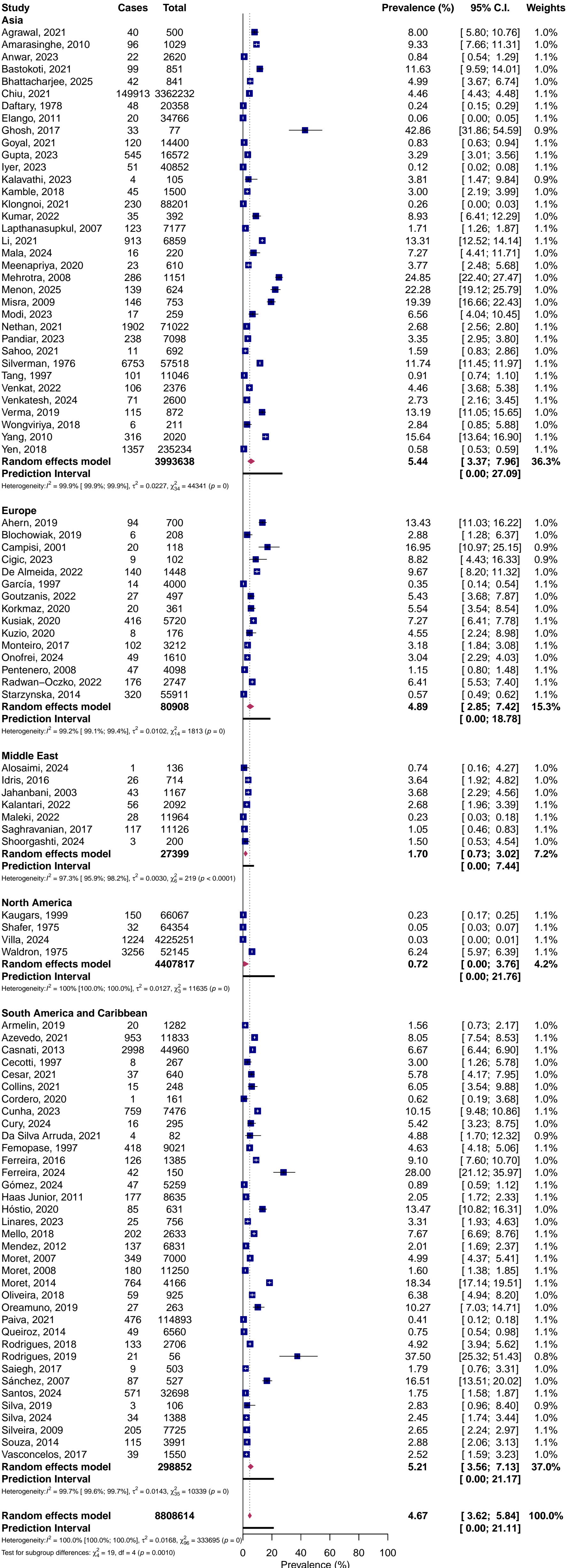

Supplement: Supplementary file 14 — Appendix S14: Sensitivity meta‐analysis of prevalence stratified by geographical region. [file JOP-55-747-s013.pdf]

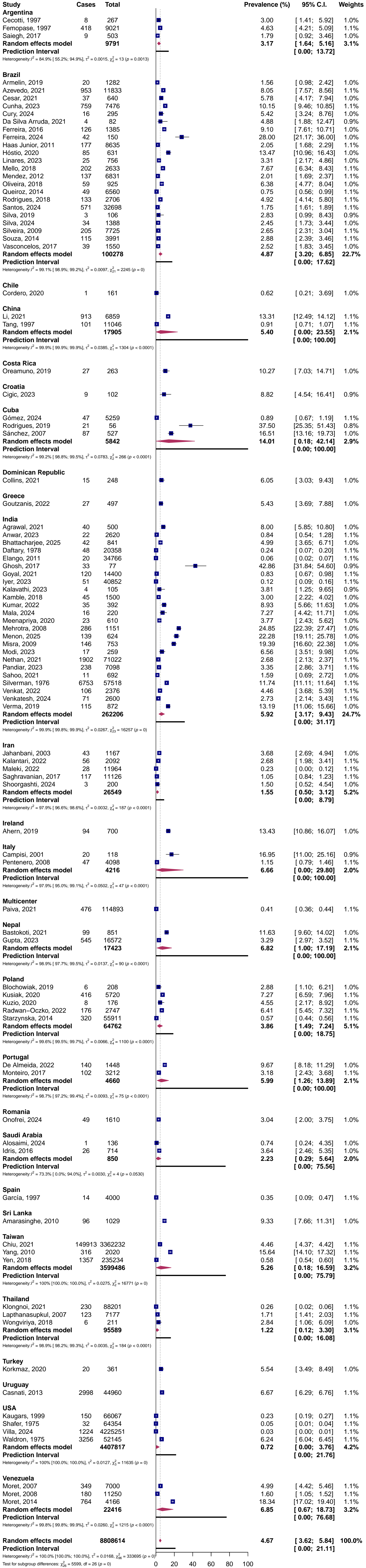

Supplement: Supplementary file 15 — Appendix S15: Sensitivity meta‐analysis of pooled prevalence by country. [file JOP-55-747-s009.pdf]

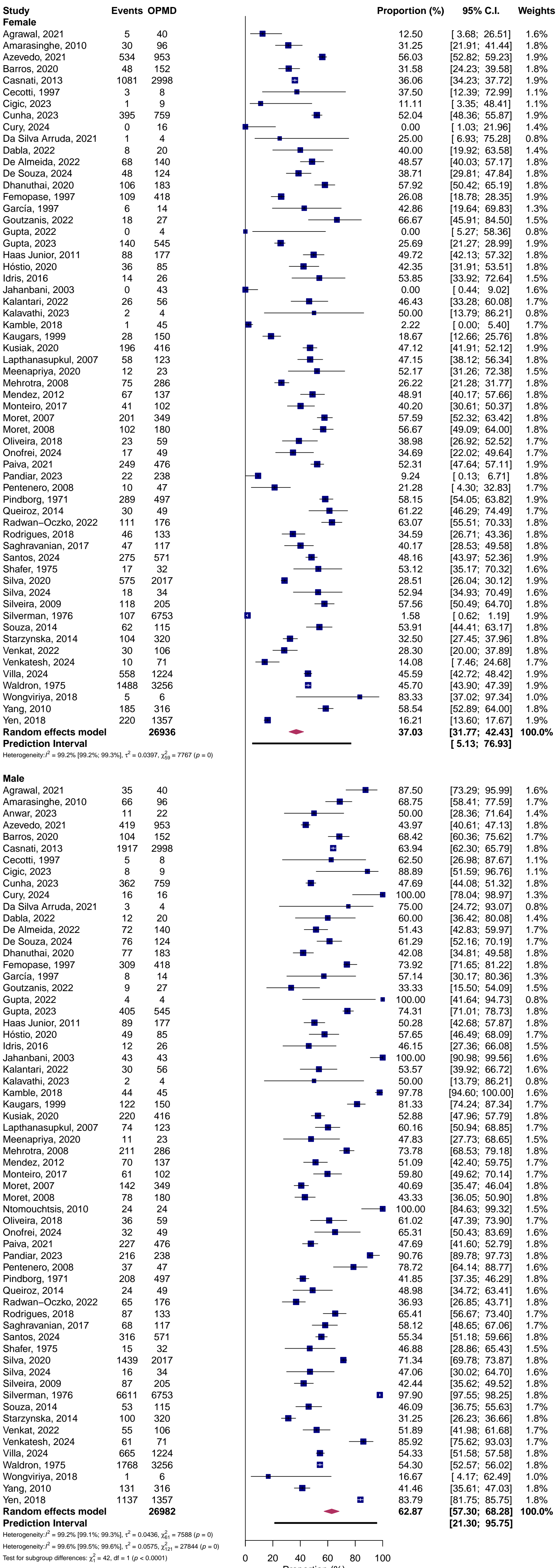

Supplement: Supplementary file 17 — Appendix S17: Meta‐analysis of pooled proportion by patient sex. [file JOP-55-747-s014.pdf]

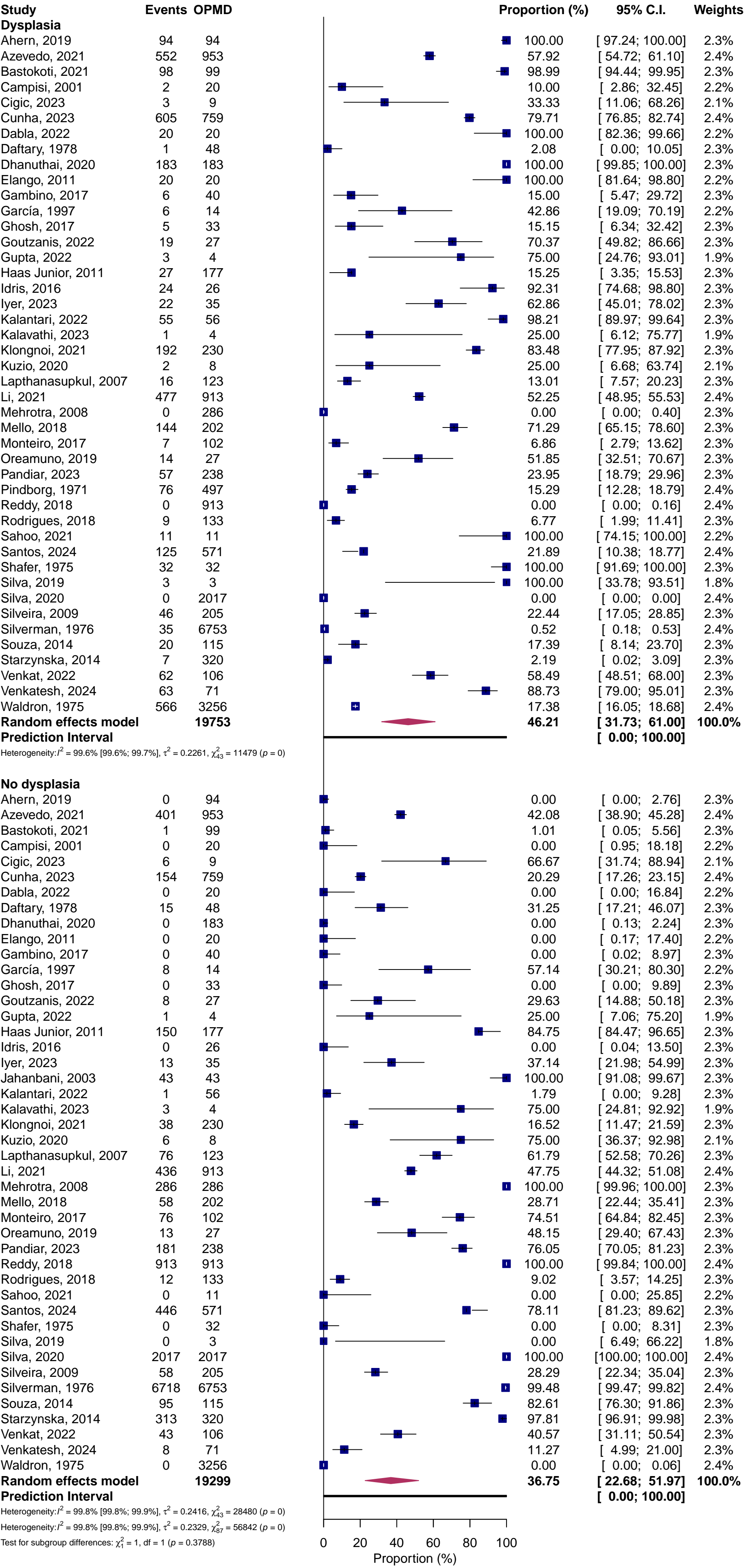

Supplement: Supplementary file 18 — Appendix S18: Meta‐analysis of pooled proportion by epithelial dysplasia. [file JOP-55-747-s018.pdf]
