# Supplementary material for: Global Prevalence of Oral Potentially Malignant Disorders: An Updated Systematic Review and Meta‐Analysis
Source: J Oral Pathol Med. 2026 Apr 28;55(7):747–54. doi: 10.1111/jop.70146 (PMC13429371; doi:10.1111/jop.70146)
Supplement: Supplementary file 12 — Appendix S12: Summary of clinical diagnosis by region. [file JOP-55-747-s008.docx]

**Appendix S12a – Population size (N), number of cases (n), and prevalence (%) of oral potentially malignant disorders per clinical diagnosis and region.** **Data are from studies included in the sensitivity meta-analysis (n=97).**

| **ASIA** | | | | | | | | |
| --- | --- | --- | --- | --- | --- | --- | --- | --- |
| **Author/year** | **Country** | **Population size (N)** | **AC n (%)** | **OE n (%)** | **OL n (%)** | **OSMF n (%)** | **PVL n (%)** | **NS n (%)** |
| **Previous Systematic Review Studies** | | | | | | | | |
| Amarasinghe et al., 2010 | Sri Lanka | 1029 |  |  | 71 (6.90%) | 25 (2.43%) |  |  |
| Lapthanasupkul et al., 2007 | Thailand | 7177 |  | 6 (0.08%) | 117 (1.63%) |  |  |  |
| Misra et al., 2009 | India | 753 |  |  | 78 (10.36%) | 68 (9.03%) |  |  |
| Silverman et al., 1976 | India | 57518 |  |  | 6753 (11.74%) |  |  |  |
| Yang et al., 2010 | Taiwan | 2020 |  | 3 (0.15%) | 224 (11.09%) | 89 (4.41%) |  |  |
| **Current Systematic Review Studies** | | | | | | | | |
| Agrawal et al., 2021 | India | 500 |  |  |  | 40 (8.00%) |  |  |
| Anwar et al., 2023 | India | 2620 |  |  | 6 (0.23%) | 11 (0.42%) | 5 (0.19%) |  |
| Bastakoti et al., 2021 | Nepal | 851 |  |  |  |  |  | 99 (11.63%) |
| Bhattacharjee et al., 2025 | India | 841 |  | 3 (0.36%) | 17 (2.02%) | 22 (2.62%) |  |  |
| Daftary et al., 1978 | India | 20358 |  |  | 48 (0.24%) |  |  |  |
| Elango et al., 2011 | India | 34766 |  |  | 20 (0.06%) |  |  |  |
| Ghosh et al., 2017 | India | 77 |  |  | 12 (15.58%) | 21 (27.27%) |  |  |
| Goyal and Goyal, 2021 | India | 14400 |  | 12 (0.08%) | 27 (0.19%) | 81 (0.56%) |  |  |
| Gupta et al., 2023 | Nepal | 16572 |  | 3 (0.02%) | 353 (2.13%) | 189 (1.14%) |  |  |
| Iyer et al., 2023 | India | 40852 |  |  | 38 (0.09%) | 13 (0.03%) | 2 (0.004%) |  |
| Kalavathi et., 2023 | India | 105 |  |  | 4 (3.81) |  |  |  |
| Kamble et al., 2018 | India | 1500 |  |  | 10 (0.67%) | 35 (2.33%) |  |  |
| Klongnoi et al., 2021 | Thailand | 88201 |  |  |  |  |  | 230 (0.26%) |
| Kumar et al., 2022 | India | 392 | 3 (0.77%) |  | 27 (6.89%) | 5 (1.28%) |  |  |
| Mala et al., 2024 | India | 220 |  |  | 6 (2.73%) | 10 (4.55%) |  |  |
| Meenapriya et al., 2020 | India | 610 | 1 (0.16%) |  | 7 (1.15%) | 13 (2.13%) |  | 2 (0.33%) |
| Mehrotra et al., 2008 | India | 1151 |  |  | 90 (7.82%) | 196 (17.03%) |  |  |
| Menon et al., 2025 | India | 624 |  |  | 61 (9.78%) | 52 (8.33%) |  | 26 (4.17%) |
| Modi et al., 2023 | India | 259 |  |  | 17 (6.56%) |  |  |  |
| Nethan et al., 2021 | India | 71022 |  |  | 1388 (1.95%) | 58 (0.08%) |  | 456 (0.64%) |
| Pandiar et al., 2023 | India | 7098 |  |  |  | 238 (3.35%) |  |  |
| Sahoo et al., 2021 | India | 692 |  |  |  |  |  | 11 (1.59%) |
| Sheng-Fu et al., 2021 | Taiwan | 3362232 |  | 15007 (0.45%) | 108869 (3.24%) | 2037 (0.06%) | 24000 (0.71%) |  |
| Tang et al., 1997 | China | 11046 |  |  |  | 101 (0.91%) |  |  |
| Venkat et al., 2022 | India | 2376 |  |  | 85 (3.58%) | 21 (0.88%) |  |  |
| Venkatesh et al., 2024 | India | 2600 |  |  | 47 (1.81%) |  | 1 (0.04%) | 23 (0.88%) |
| Verma and Sharma, 2019 | India | 872 |  | 20 (2.29%) | 41 (4.70%) | 54 (6.19%) |  |  |
| Wongviriya et al., 2018 | Thailand | 211 |  |  | 6 (2.84%) |  |  |  |
| Yen et al., 2018 | Taiwan | 235234 |  |  |  |  |  | 1357 (0.58%) |

| **SOUTH AMERICA AND CARIBBEAN** | | | | | | | | |
| --- | --- | --- | --- | --- | --- | --- | --- | --- |
| **Author/year** | **Country** | **Population size (N)** | **AC n (%)** | **OE n (%)** | **OL n (%)** | **OSMF n (%)** | **PVL n (%)** | **NS n (%)** |
| **Previous Systematic Review Studies** | | | | | | | | |
| Cecotti et al., 1997 | Argentina | 267 |  |  | 8 (3.00%) |  |  |  |
| Femopase et al., 1997 | Argentina | 9021 |  |  | 418 (4.63%) |  |  |  |
| Haas Junior et al., 2011 | Brazil | 8635 |  |  | 177 (2.05%) |  |  |  |
| Mendez et al., 2012 | Brazil | 6831 |  |  | 137 (2.01%) |  |  |  |
| Queiroz et al., 2014 | Brazil | 6560 |  | 8 (0.12%) | 41 (0.62%) |  |  |  |
| Sánchez et al., 2007 | Cuba | 527 |  | 6 (1.14%) | 81 (15.37%) |  |  |  |
| Silveira et al., 2009 | Brazil | 7725 | 33 (0.43%) | 20 (0.26%) | 152 (1.97%) |  |  |  |
| Souza et al., 2014 | Brazil | 3991 |  |  | 115 (2.88%) |  |  |  |
| **Current Systematic Review Studies** | | | | | | | | |
| Armelin et al., 2019 | Brazil | 1282 | 3 (0.23%) | 4 (0.31%) | 13 (1.01%) |  |  |  |
| Azevedo et al., 2021 | Brazil | 11833 | 145 (1.23%) | 8 (0.07%) | 800 (6.76%) |  |  |  |
| Casnati et al., 2013 | Uruguay | 44960 |  |  | 2998 (6.67%) |  |  |  |
| Cesar et al., 2021 | Brazil | 640 | 21 (3.28%) |  | 16 (2.5%) |  |  |  |
| Collins et al., 2021 | Dominican Republic | 248 | 2 (0.81%) |  | 13 (5.24%) |  |  |  |
| Cordero et al., 2020 | Chile | 161 |  |  | 1 (0.62%) |  |  |  |
| Cunha et al., 2023 | Brazil | 7476 | 122 (1.63%) |  |  |  |  | 637 (8.52%) |
| Cury et al., 2024 | Brazil | 295 | 14 (4.75%) |  | 2 (0.68%) |  |  |  |
| da Silva Arruda et al., 2021 | Brazil | 82 | 2 (2.44%) |  | 2 (2.44%) |  |  |  |
| Ferreira et al., 2016 | Brazil | 1385 | 116 (8.38%) | 1 (0.07%) | 9 (0.65%) |  |  |  |
| Ferreira et al., 2024 | Brazil | 150 | 38 (25.33%) |  | 4 (2.67%) |  |  |  |
| Gómez et al., 2024 | Cuba | 5259 |  | 10 (0.19%) | 37 (0.70%) |  |  |  |
| Hóstio et al., 2020 | Brazil | 631 | 59 (9.35%) | 3 (0.48%) | 23 (3.65%) |  |  |  |
| Linares et al., 2023 | Brazil | 756 | 13 (1.72%) |  | 12 (1.59%) |  |  |  |
| Mello et al., 2018 | Brazil | 2633 |  | 9 (0.34%) | 193 (7.33%) |  |  |  |
| Moret et al., 2007 | Venezuela | 7000 | 5 (0.07%) | 1 (0.01%) | 343 (4.90%) |  |  |  |
| Moret et al, 2008 | Venezuela | 11250 |  |  | 180 (1.60%) |  |  |  |
| Moret, 2014 | Venezuela | 4166 |  |  | 764 (18.34%) |  |  |  |
| Oliveira et al., 2018 | Brazil | 925 |  |  | 59 (6.38%) |  |  |  |
| Oreamuno et al., 2019 | Costa Rica | 263 | 12 (4.56%) |  | 15 (5.70%) |  |  |  |
| Paiva e Costa, 2021 | Brazil/Mexico/Argentina | 114893 |  |  | 476 (0.41%) |  |  |  |
| Rodrigues et al., 2018 | Brazil | 2706 | 68 (2.51%) | 4 (0.15%) | 61 (2.25%) |  |  |  |
| Rodríguez et al., 2019 | Cuba | 56 |  | 5 (8.93%) | 16 (28.57%) |  |  |  |
| Saiegh et al., 2017 | Argentina | 503 | 3 (0.60%) |  | 6 (1.19%) |  |  |  |
| Santos et al., 2024 | Brazil | 32698 | 46 (0.14%) |  | 505 (1.54%) |  |  | 20 (0.06%) |
| Silva et al., 2019 | Brazil | 106 | 3 (2.83%) |  |  |  |  |  |
| Silva et al., 2024 | Brazil | 1388 | 17 (1.22%) |  | 17 (1.22%) |  |  |  |
| Vasconcelos et al., 2017 | Brazil | 1550 | 39 (2.52%) |  |  |  |  |  |

| **EUROPE** | | | | | | | | |
| --- | --- | --- | --- | --- | --- | --- | --- | --- |
| **Author/year** | **Country** | **Population size (N)** | **AC n (%)** | **OE n (%)** | **OL n (%)** | **OSMF n (%)** | **PVL n (%)** | **NS n (%)** |
| **Previous Systematic Review Studies** | | | | | | | | |
| Campisi and Margiotta., 2001 | Italy | 118 | 5 (4.24%) |  | 15 (12.71%) |  |  |  |
| Garcia et al., 1997 | Spain | 4000 |  |  | 14 (0.35%) |  |  |  |
| Pentenero et al., 2008 | Italy | 4098 |  |  | 47 (1.15%) |  |  |  |
| Starzynska et al., 2014 | Poland | 55911 |  |  | 320 (0.57%) |  |  |  |
| **Current Systematic Review Studies** | | | | | | | | |
| Ahern et al., 2019 | Ireland | 700 |  |  |  |  |  | 94 (13.43%) |
| Blochowiak et al., 2019 | Poland | 208 |  |  | 6 (2.88%) |  |  |  |
| Cigic et al., 2023 | Croatia | 102 | 2 (1.96%) | 3 (2.94) | 4 (3.92%) |  |  |  |
| De Almeida et al., 2022 | Portugal | 1448 |  |  | 140 (9.67%) |  |  |  |
| Goutzanis et al., 2022 | Greece | 497 |  |  | 27 (5.43%) |  |  |  |
| Korkmaz et al., 2020 | Turkey | 361 |  | 6 (1.66%) | 14 (3.88%) |  |  |  |
| Kusiak et al., 2020 | Poland | 5720 |  |  | 416 (7.27%) |  |  |  |
| Kuzio et al., 2020 | Poland | 176 |  |  | 8 (4.55%) |  |  |  |
| Monteiro et al., 2017 | Portugal | 3212 | 18 (0.56%) | 1 (0.03%) | 83 (2.58%) |  |  |  |
| Onofrei et al., 2024 | Romania | 1610 | 27 (1.68%) |  | 22 (1.37%) |  |  |  |
| Radwan et al., 2022 | Poland | 2747 |  |  | 176 (6.41%) |  |  |  |

| **NORTH AMERICA** | | | | | | | | |
| --- | --- | --- | --- | --- | --- | --- | --- | --- |
| **Author/year** | **Country** | **Population size (N)** | **AC n (%)** | **OE n (%)** | **OL n (%)** | **OSMF n (%)** | **PVL n (%)** | **NS n (%)** |
| **Previous Systematic Review Studies** | | | | | | | | |
| Kaugars et al., 1999 | USA | 66067 | 150 (0.23%) |  |  |  |  |  |
| Shafer and Waldron, 1975 | USA | 64354 |  | 32 (0.05%) |  |  |  |  |
| **Current Systematic Review Studies** | | | | | | | | |
| Villa et al., 2024 | USA | 4225251 |  | 22 (0.0005%) | 1124 (0.03%) | 78 (0.0018%) |  |  |
| Waldron et al., 1975 | USA | 52145 |  |  | 3256 (6.24%) |  |  |  |

| **MIDDLE EAST** | | | | | | | | |
| --- | --- | --- | --- | --- | --- | --- | --- | --- |
| **Author/year** | **Country** | **Population size (N)** | **AC n (%)** | **OE n (%)** | **OL n (%)** | **OSMF n (%)** | **PVL n (%)** | **NS n (%)** |
| **Previous Systematic Review Studies** | | | | | | | | |
| Idris et al., 2016 | Saudi Arabia | 714 |  |  |  |  |  | 26 (3.64%) |
| Jahanbani et al., 2003 | Iran | 1167 |  |  | 43 (3.68%) |  |  |  |
| **Current Systematic Review Studies** | | | | | | | | |
| Alosaimi et al., 2024 | Saudi Arabia | 136 |  |  |  | 1 (0.74%) |  |  |
| Kalantari et al., 2022 | Iran | 2092 | 1 (0.04%) |  | 55 (2.62%) |  |  |  |
| Maleki et al., 2022 | Iran | 11964 |  |  | 28 (0.23%) |  |  |  |
| Saghravanian et al., 2017 | Iran | 11126 |  | 2 (0.02%) | 115 (1.03%) |  |  |  |
| Shoorgashti et al., 2024 | Iran | 200 |  |  | 3 (1.50%) |  |  |  |

**Legend:** AC: actinic cheilitis; PVL: proliferative verrucous leukoplakia; OE: oral erythroplakia; OL: oral leukoplakia; OSMF: oral submucous fibrosis; NS: non-specific (grouped lesions).

**Appendix S12b.** Summary of the number of cases of oral potentially malignant disorders and total population across geographic regions. Data are from studies included in the sensitivity meta-analysis (n=97*). The percentage was calculated per row, reflecting the distribution per geographic location.

| Clinical diagnosis** | Asia  (35 studies) | South America and Caribbean  (36 studies) | Europe  (15 studies) | North America  (4 studies) | Middle East  (7 studies) | All regions  (97 studies) |
| --- | --- | --- | --- | --- | --- | --- |
| Oral leukoplakia | 118418 (89.69%) | 7694 (5.83%) | 1292 (0.98%) | 4380 (3.32%) | 244 (0.18%) | 132028 (100.00%) |
| Actinic cheilitis | 4 (0.41%) | 759 (77.69%) | 52 (5.32%) | 150 (15.35%) | 12 (1.23%) | 977 (100.00%) |
| Oral submucous fibrosis | 3379 (97.72%) | 0 (0.00%) | 0 (0.00%) | 78 (2.26%) | 1 (0.03%) | 3458 (100.00%) |
| Oral erythroplakia | 15054 (99.10%) | 71 (0.47%) | 10 (0.07%) | 54 (0.36%) | 2 (0.01%) | 15191 (100.00%) |
| OPMD grouped  (non-specified) | 2004 (72.06%) | 657 (23.62%) | 94 (3.38%) | 0 (0.00%) | 26 (0.93%) | 2781 (100.00%) |
| Proliferative verrucous leukoplakia | 24008 (100.00%) | 0 (0.00%) | 0 (0.00%) | 0 (0.00%) | 0 (0.00%) | 24008 (100.00%) |
| Total (OPMD cases) | 162867 (91.30%) | 9181 (5.10%) | 1448 (0.80%) | 4662 (2.60%) | 285 (0.20%) | 178443 (100.00%) |
| Total (population) | 3993638 (45.34%) | 298852 (3.39%) | 80908 (0.92%) | 4407817 (50.04%) | 27399 (0.31%) | 8808614 (100.00%) |

**Legend:** OPMD: Oral potentially malignant disorder. *Studies presented here exclude those limited to predetermined anatomical sites (lip, palate, tongue, or gingiva). **Erythroleukoplakia and leukoerythroplakia were included under the category of oral leukoplakia.
