# Supplementary material for: Global Prevalence of Oral Potentially Malignant Disorders: An Updated Systematic Review and Meta‐Analysis
Source: J Oral Pathol Med. 2026 Apr 28;55(7):747–54. doi: 10.1111/jop.70146 (PMC13429371; doi:10.1111/jop.70146)

Study	Events	OPMD	Proportion (%)	95% C.I.	Weights
**Over 50y**					
Cesar, 2021	37	37	100.00	[90.92; 100.00]	4.2%
Cunha, 2023	759	759	100.00	[99.60; 100.00]	4.5%
Dabla, 2022	2	20	10.00	[2.78; 32.42]	4.0%
Gupta, 2023	190	545	34.86	[30.79; 38.99]	4.5%
Haas Junior, 2011	97	177	54.80	[47.15; 62.31]	4.5%
Hóstio, 2020	57	85	67.06	[55.95; 76.57]	4.4%
Idris, 2016	17	26	65.38	[44.52; 81.70]	4.1%
Lapthanasupkul, 2007	76	123	61.79	[52.70; 71.22]	4.4%
Meenapriya, 2020	12	23	52.17	[31.30; 72.33]	4.1%
Mendez, 2012	117	137	85.40	[78.17; 90.58]	4.4%
Moret, 2007	195	349	55.87	[50.49; 61.26]	4.5%
Moret, 2008	54	180	30.00	[23.51; 37.31]	4.5%
Oliveira, 2018	51	59	86.44	[75.65; 94.98]	4.3%
Onofrei, 2024	44	49	89.80	[77.89; 96.78]	4.3%
Paiva, 2021	353	476	74.16	[70.27; 78.45]	4.5%
Pandiar, 2023	59	238	24.79	[19.31; 30.70]	4.5%
Saghravanian, 2017	76	117	64.96	[55.56; 73.40]	4.4%
Santos, 2024	405	571	70.93	[67.33; 75.08]	4.5%
Silva, 2020	1427	2017	70.75	[68.73; 72.76]	4.5%
Starzynska, 2014	86	320	26.88	[21.14; 31.47]	4.5%
Waldron, 1975	1862	3256	57.19	[55.48; 58.92]	4.5%
Wongviriya, 2018	6	6	100.00	[53.55; 96.62]	3.3%
Yen, 2018	1357	1357	100.00	[99.91; 100.00]	4.5%
**Random effects model**		**10927**	**68.10**	**[54.90; 80.01]**	**100.0%**
**Prediction Interval**				**[7.29; 100.00]**	
Heterogeneity: I^2 = 99.4% [99.4%; 99.5%], τ^2 = 0.1027, χ^2_{22} = 3954 (p = 0)					
**Under 50y**					
Agrawal, 2021	40	40	100.00	[90.54; 99.72]	5.1%
Dabla, 2022	18	20	90.00	[67.55; 97.14]	4.8%
Gupta, 2023	355	545	65.14	[60.95; 69.11]	5.4%
Haas Junior, 2011	76	177	42.94	[35.58; 50.58]	5.4%
Hóstio, 2020	20	85	23.53	[15.36; 34.14]	5.3%
Idris, 2016	5	26	19.23	[6.14; 39.24]	4.9%
Lapthanasupkul, 2007	52	123	42.28	[33.49; 51.50]	5.3%
Meenapriya, 2020	11	23	47.83	[27.75; 68.63]	4.8%
Mendez, 2012	20	137	14.60	[8.97; 21.52]	5.3%
Moret, 2007	148	349	42.41	[35.23; 47.45]	5.4%
Oliveira, 2018	7	59	11.86	[5.55; 23.33]	5.2%
Onofrei, 2024	5	49	10.20	[2.54; 21.63]	5.2%
Paiva, 2021	101	476	21.22	[16.84; 24.56]	5.4%
Pandiar, 2023	108	238	45.38	[38.80; 51.96]	5.4%
Saghravanian, 2017	33	117	28.21	[20.50; 37.37]	5.3%
Santos, 2024	232	571	40.63	[36.48; 44.75]	5.4%
Silva, 2020	485	2017	24.05	[22.15; 25.93]	5.4%
Starzynska, 2014	18	320	5.62	[2.63; 8.06]	5.4%
Waldron, 1975	1272	3256	39.07	[37.37; 40.75]	5.5%
**Random effects model**		**8628**	**36.94**	**[24.53; 50.28]**	**100.0%**
**Prediction Interval**				**[0.00; 92.44]**	
Heterogeneity: I^2 = 97.9% [97.4%; 98.3%], τ^2 = 0.0845, χ^2_{18} = 843 (p < 0.0001)					

Heterogeneity: I^2 = 99.5% [99.4%; 99.5%], τ^2 = 0.1175, χ^2_{41} = 7683 (p = 0)

Test for subgroup differences: χ^2_1 = 11, df = 1 (p = 0.0012)

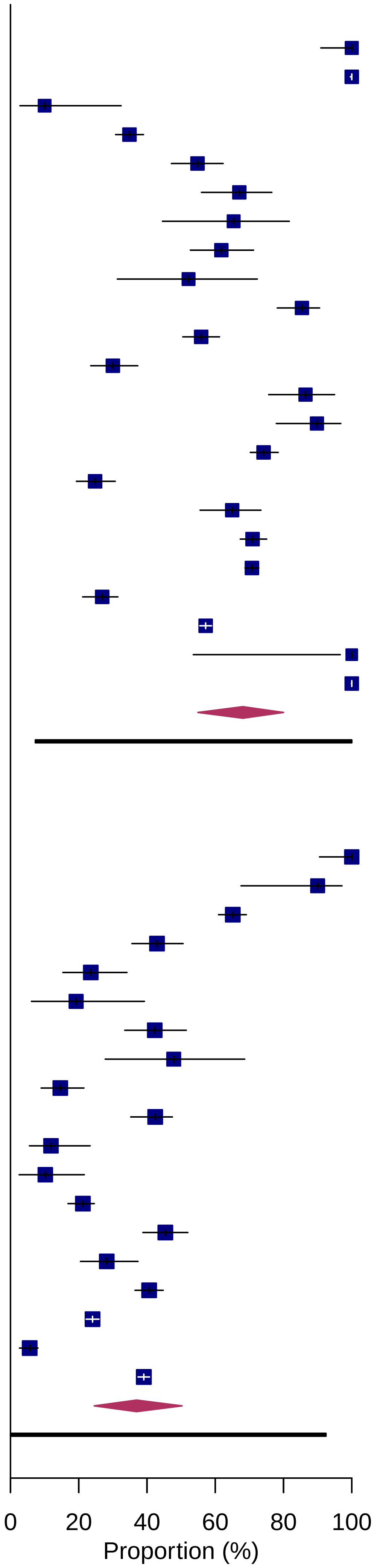

Supplement: Supplementary file 16 — Appendix S16: Meta‐analysis of pooled proportion by patient age. [file JOP-55-747-s003.pdf]
