# Supplementary material for: Global Prevalence of Oral Potentially Malignant Disorders: An Updated Systematic Review and Meta‐Analysis
Source: J Oral Pathol Med. 2026 Apr 28;55(7):747–54. doi: 10.1111/jop.70146 (PMC13429371; doi:10.1111/jop.70146)
Supplement: Supplementary file 19 — Data S1: Supporting Information. [file JOP-55-747-s011.docx]

**Journal:** Journal of Oral Pathology & Medicine

**Manuscript ID:** 1664963

**Title:** Global prevalence of oral potentially malignant disorders: An updated systematic review and meta-analysis.

**Authors:** Nicole Lonni, Tulio Silva Rosa, Camilla Kammer Pereira, Gilberto Melo, Saman Warnakulasuriya, Eliete Neves Silva Guerra, Elena Riet Correa Rivero

**Corresponding author, affiliation, and e-mail:** Elena Riet Correa Rivero, Universidade Federal de Santa Catarina, riet.elena@gmail.com.

**Supplementary Materials – Captions and legends**

**Supplementary Material 1**

**[Title] Appendix S1. Database search strategy**

**[Legend]**

*All searches were conducted with a filter set to the timeframe 2017-2025, taking into account that this is an update from previous work.

**Supplementary Material 2**

**[Title] Appendix S2. Reasons for exclusion of studies following full-text screening (n=330).**

**[Legend]**

Exclusion criteria applied: (1) OPMD data could not be extracted due to grouping with other conditions; (2) OPMDs were only linked to specific etiological factors (e.g., HPV, betel quid, tobacco); (3) diagnosis of OPMD was not confirmed by histopathological analysis; (4) prevalence of OPMD was not clearly reported or could not be calculated; (5) reviews, case reports, protocols, short communications, personal opinions, letters, posters, conference abstracts, thesis, dissertations, and laboratory research; (6) full texts were not available; (7) only oral lichen planus or other lichenoid lesions were included in the analysis, and (8) published in languages other than the Latin (Roman) alphabet

**Supplementary Material 3**

**[Title] Appendix S3. Summary of the included studies (n=108)**

**[Legend]**

**AC**, actinic cheilitis; **EA**, epithelial acanthosis; **ED**, epithelial dysplasia; **EHP**, epithelial hyperplasia; **F**, females; **HK**, hyperkeratosis; **HKA**, hyperkeratosis and acanthosis; **HOE**, homogeneous oral erythroplakia; **HOL**, homogeneous oral leukoplakia; **HOK**, hyperorthokeratosis; **HOPK**, hyperortho/parakeratosis; **HPK** hyperparakeratosis; **HP**, hyperplasia; **IQR**, interquartile range; **LS**, lesions; **M**, males; **MiED**, mild epithelial dysplasia; **MoED**, moderate epithelial dysplasia; **nED**, non-dysplastic; **NHOL**, non-homogeneous leukoplakia; **NI**: cases in which leukoplakia subtype (homogeneous/non-homogeneous) was not specified; **NK**, non-keratinization; **NR**, not reported; **OE**, oral erythroplakia; **OL**, oral leukoplakia; **OPMD**, oral potentially malignant disorders; **OSMF**, oral submucous fibrosis; **psHP**, pseudoepitheliomatous hyperplasia; **SE**, solar elastosis; **SED**, severe epithelial dysplasia; **SD**, standard deviation; **USA**, United States of America; **PVL**, proliferative verrucous leucoplakia; **VHP**, verrucous hyperplasia. The type of study, when provided, was reported as stated by the authors of the included studies. **¥** Cases in which oral lichen planus, OSCC, or dysplasia were reported as clinical diagnoses—or classified as unspecified clinical diagnoses—were excluded. Clinicopathological data were extracted only when they could be analyzed independently for the selected sample. Erytroleukoplakia and leukoerythroplakia were described as NHOL. *****Studies excluded in the sensitivity meta-analyses were those restricted to predetermined anatomical sites (lip, palate, tongue, or gingiva).

**Supplementary Material 4**

**[Title] Appendix S4. Methodological quality in individual studies**

**[Legend]**

Yes

Unclear

No

**N/A** Not applicable

**Questions:**

**1** Was the sample frame appropriate to address the target population?

**2** Were study participants sampled in an appropriate way?

**3** Was the sample size adequate?

**4** Were the study subjects and the setting described in detail?

**5** Was the data analysis conducted with sufficient coverage of the identified sample?

**6** Were valid methods used for the identification of the condition?

**7** Was the condition measured in a standard, reliable way for all participants?

**8** Was there appropriate statistical analysis?

**9** Was the response rate adequate, and if not, was the low response rate managed appropriately?

**Supplementary Material 5**

**[Title] Appendix S5. Meta-analysis: Global prevalence of oral potentially malignant disorders.**

**[Legend]**

**Abbreviations:** CI, confidence interval; I², heterogeneity index; τ², between-study variance; χ², chi-square test for heterogeneity

**Supplementary Material 6**

**[Title] Appendix S6. Sensitivity meta-analysis: Global prevalence of oral potentially malignant disorders.**

**[Legend]**

**Abbreviations:** CI, confidence interval; I², heterogeneity index; τ², between-study variance; χ², chi-square test for heterogeneity

**Supplementary Material 7**

**[Title] Appendix S7. Sensitivity meta-analysis: Pooled prevalence by diagnosis in Asia.**

**[Legend]**

**Abbreviations:** AC, actinic cheilitis; OE, oral erythroplakia; OL, oral leukoplakia; HOL, homogeneous oral leukoplakia; NHOL, non-homogeneous leukoplakia; OPMD, oral potentially malignant disorders; OSMF, oral submucous fibrosis; PVL, proliferative verrucous leucoplakia; CI, confidence interval; I², heterogeneity index; τ², between-study variance; χ², chi-square test for heterogeneity

**Supplementary Material 8**

**[Title] Appendix S8. Sensitivity meta-analysis: Pooled prevalence by diagnosis in South America and Caribbean.**

**[Legend]**

**Abbreviations:** AC, actinic cheilitis; OE, oral erythroplakia; OL, oral leukoplakia; HOL, homogeneous oral leukoplakia; NHOL, non-homogeneous leukoplakia; OPMD, oral potentially malignant disorders; CI, confidence interval; I², heterogeneity index; τ², between-study variance; χ², chi-square test for heterogeneity

**Supplementary Material 9**

**[Title] Appendix S9. Sensitivity meta-analysis: Pooled prevalence by diagnosis in Europe.**

**[Legend]**

**Abbreviations:** AC, actinic cheilitis; OE, oral erythroplakia; OL, oral leukoplakia; HOL, homogeneous oral leukoplakia; NHOL, non-homogeneous leukoplakia; OPMD, oral potentially malignant disorders; CI, confidence interval; I², heterogeneity index; τ², between-study variance; χ², chi-square test for heterogeneity

**Supplementary Material 10**

**[Title] Appendix S10. Sensitivity meta-analysis: Pooled prevalence by diagnosis in North America.**

**[Legend]**

**Abbreviations:** AC, actinic cheilitis; OE, oral erythroplakia; OL, oral leukoplakia; OSMF, oral submucous fibrosis; CI, confidence interval; I², heterogeneity index; τ², between-study variance; χ², chi-square test for heterogeneity

**Supplementary Material 11**

**[Title] Appendix S11. Sensitivity meta-analysis: Pooled prevalence by diagnosis in Middle East.**

**[Legend]**

**Abbreviations:** AC, actinic cheilitis; OE, oral erythroplakia; OL, oral leukoplakia; NHOL, non-homogeneous leukoplakia; OPMD, oral potentially malignant disorders; OSMF, oral submucous fibrosis; CI, confidence interval; I², heterogeneity index; τ², between-study variance; χ², chi-square test for heterogeneity

**Supplementary Material 12**

**[Title] Appendix S12a – Population size (N), number of cases (n), and prevalence (%) of oral potentially malignant disorders per clinical diagnosis and region. Data are from studies included in the sensitivity meta-analysis (n=97).**

**[Legend]**

AC: actinic cheilitis; PVL: proliferative verrucous leukoplakia; OE: oral erythroplakia; OL: oral leukoplakia; OSMF: oral submucous fibrosis; NS: non-specific (grouped lesions).

**[Title] Appendix S12b. Summary of the number of cases of oral potentially malignant disorders and total population across geographic regions. Data are from studies included in the sensitivity meta-analysis (n=97*). The percentage was calculated per row, reflecting the distribution per geographic location.**

**[Legend]**

OPMD: Oral potentially malignant disorder. *Studies presented here exclude those limited to predetermined anatomical sites (lip, palate, tongue, or gingiva). **Erythroleukoplakia and leukoerythroplakia were included under the category of oral leukoplakia.

**Supplementary Material 13**

**[Title] Appendix S13. Sensitivity meta-analysis: Pooled prevalence by sample source.**

**[Legend]**

**Abbreviations:** CI, confidence interval; I², heterogeneity index; τ², between-study variance; χ², chi-square test for heterogeneity

**Supplementary Material 14**

**[Title] Appendix S14. Sensitivity meta-analysis of prevalence stratified by geographical region.**

**[Legend]**

**Abbreviations:** CI, confidence interval; I², heterogeneity index; τ², between-study variance; χ², chi-square test for heterogeneity

**Supplementary Material 15**

**[Title] Appendix S15. Sensitivity meta-analysis: Pooled prevalence by country.**

**[Legend]**

**Abbreviations:** CI, confidence interval; I², heterogeneity index; τ², between-study variance; χ², chi-square test for heterogeneity

**Supplementary Material 16**

**[Title] Appendix S16. Meta-analysis: Pooled proportion by patient age (<50 and ≥50 years).**

**[Legend]**

**Abbreviations:** CI, confidence interval; I², heterogeneity index; τ², between-study variance; χ², chi-square test for heterogeneity; OPMD: oral potentially malignant disorder.

**Supplementary Material 17**

**[Title] Appendix S17. Meta-analysis: Pooled proportion by patient sex.**

**[Legend]**

**Abbreviations:** CI, confidence interval; I², heterogeneity index; τ², between-study variance; χ², chi-square test for heterogeneity; OPMD: oral potentially malignant disorder.

**Supplementary Material 18**

**[Title] Appendix S18. Meta-analysis: Pooled proportion by epithelial dysplasia.**

**[Legend]**

**Abbreviations:** CI, confidence interval; I², heterogeneity index; τ², between-study variance; χ², chi-square test for heterogeneity; OPMD: oral potentially malignant disorder.
